# Assessment of virulence potential of uncharacterized *Enterococcus faecalis* strains using pan genomic approach – Identification of pathogen–specific and habitat-specific genes

**DOI:** 10.1038/srep38648

**Published:** 2016-12-07

**Authors:** Utpal Bakshi, Munmun Sarkar, Sandip Paul, Chitra Dutta

**Affiliations:** 1 Structural Biology & Bioinformatics Division, CSIR- Indian Institute of Chemical Biology, 4, Raja S. C. Mullick Road, Kolkata 700032, India; 2 Academy of Scientific and Innovative Research (AcSIR), CSIR- Indian Institute of Chemical Biology (CSIR-IICB), 4, Raja S. C. Mullick Road, Kolkata 700032, India

## Abstract

*Enterococcus faecalis*, a leading nosocomial pathogen and yet a prominent member of gut microbiome, lacks clear demarcation between pathogenic and non-pathogenic strains at genome level. Here we present the comparative genome analysis of 36 *E. faecalis* strains with different pathogenic features and from different body-habitats. This study begins by addressing the genome dynamics, which shows that the pan-genome of *E. faecalis* is still open, though the core genome is nearly saturated. We identified eight uncharacterized strains as potential pathogens on the basis of their co-segregation with reported pathogens in gene presence-absence matrix and Pathogenicity Island (PAI) distribution. A ~7.4 kb genomic-cassette, which is itself a part of PAI, is found to exist in all reported and potential pathogens, but not in commensals and other uncharacterized strains. This region encodes four genes and among them, products of two hypothetical genes are predicted to be intrinsically disordered that may serve as novel targets for therapeutic measures. Exclusive existence of 215, 129, 4 and 1 genes in the blood, gastrointestinal tract, urogenital tract, oral cavity and lymph node derived *E. faecalis* genomes respectively suggests possible employment of distinct habitat-specific genetic strategies in the adaptation of *E. faecalis* in human host.

*Enterococci*, a member of the human microbiome community, have gained notoriety over the past few decades as the leading nosocomial pathogens^1^. They have been recognized as the second-most common cause of urinary tract infections and the third-most common cause of nosocomial bacteraemia^2^. Although more than a dozen different enterococcal species are known to be associated with human disease, *Enterococcus faecalis* is the most frequently recovered representing more than 90% of clinical isolates^3^, followed by *Enterococcus faecium.* A conspicuous feature of *E. faecalis* is remarkable cross-strain diversity in the gene repertoire, especially in the antibiotic resistance genes, virulence determinants and pathogenicity island (PAI) contents^4^. Though *E. faecium* is better known for its multi-drug resistance characteristics^5^, *E. faecalis* seems to harbor, in addition to an array of antibiotic resistance traits^6^, a greater repertoire of potential virulence determinants^7^. The major factors contributing to the genetic diversity of *E. faecalis* include a high rate of recombination^8^, extremely high abundance of transposable elements and the advanced pheromone system facilitating transfer of large genomic regions including virulence and resistance genes^9^,^10^.

Molecular epidemiological studies revealed existence of diverse sequence types (STs) of *E. faecalis* belonging to distinct clonal complexes (CCs) that were associated with nosocomial outbreaks and infections in different continents at different times. Analysis of 110 *E. faecalis* isolates by Multi Locus Sequence Typing (MLST)^8^ identified 55 STs and 4 major CCs, two of which (CC2 and CC9) were significantly enriched among nosocomial isolates. Several other *E. faecalis* CCs or STs, such as CC87, CC21, ST6, ST9, ST40 etc., are also known to be associated with clinical isolates in restricted geographic region^8^. But no clear demarcation could be made yet between the pathogenic and non-pathogenic *E. faecalis* in terms of their gene repertoire. Most *E. faecalis* CCs contain both clinical and commensal isolates, suggesting that the clinical isolates may not be evolutionarily distinct from the commensal ones, as observed in *E. faecium*^11^. Molecular studies showed frequent occurrences of the known virulence genes like cytolysin, gelatinase or the surface protein Esp in strains isolated from food or healthy infants^12^,^13^, suggesting that existence of mere presence of these virulence factors might not call for pathogenic phenotypes in an *E. faecalis* strain. There may exist other virulence factors that are yet to be characterized.

One of the major reasons behind the ambiguous virulent phenotypes of *E. faecalis* strains could be the highly evolving nature of its Pathogenicity Island (PAI). Most of the virulence factors of *E. faecalis* are known to be harbored by an island of modular structure, where each module has probably been acquired through HGT events^14^. Comparative studies of the PAIs in virulent *E. faecalis* strains revealed substantial cross-strain variations in PAI gene content, even within a clonal lineage, suggesting that different segments of the island can vary independently^15^. Identification of novel gene modules in virulent strain type ST6 that were previously not thought to be associated with the *E. faecalis* PAI also pointed towards the dynamic character of the island^16^. It has been reported that the modular integrity of PAIs does not correlate with the evolutionary relatedness of the respective strains as determined by MLST analysis^10^,^11^ and a clear idea about the genesis of PAI is yet to be emerged.

In an attempt to identify the key factors of *E. faecalis* pathogenesis, we have carried out a pan-genomic analysis of 36 *E. faecalis* strains −33 draft genomes from the initial catalog of Human Microbiome Project (HMP) consortium^17^ and 3 other complete genome sequences available in the NCBI database at the time of initiation of this study. Among these, 11 strains are well characterized pathogens and 3 are experimentally characterized non-virulent strains, but the pathogenic potential of other 22 strains are not known yet. The primary objectives of this analysis were to elucidate the genetic divergences between the known pathogenic and commensal strains, to identify the genes/PAI modules that exist exclusively in established/predicted pathogenic *E. faecalis*, but not in their benign relatives and to assess the virulence potential of uncharacterized strains on the basis of such distinct pathogen-specific gene repertoire. An attempt has also been made to identify the genes present exclusively in established/predicted pathogenic strains, not in the commensals.

Another objective of this study was to explore the habitat-specific variations in the gene repertoire of 33 microbiome-derived *E. faecalis* genomes under study. Earlier studies indicated that within a human host, different body habitats create unique niches for the resident microbiota^18^ and to adapt at different body niches, the microbiome components may undergo niche-specific variations in their gene repertoire^19^,^20^. With a view to identify the niche-specific genes, if any, in *E. faecalis*, we have analyzed the gene repertoire of the strains derived from blood (16 strains), gastrointestinal tract (GIT) (12 strains), urogenital tract (UGT) (5 strains), oral cavity (2 strains) and lymph node (1 strain) of human^17^ (Table 1).

## Results and Discussion

### Accessory genes repertoire reflects higher genetic diversity among *E. faecalis* genomes

With a view to comprehend the complete genetic landscape of *E. faecalis,* the pan-genome of the species was constructed on the basis of 36 strains under study. The strains vary in their genome sizes (2.8–3.3 Mb) and the number of predicted protein-coding sequences (from 2759 to 3510), corroborating the notion of frequent occurrences of lateral gene transfer in this organism. In this study, the pathogenic and commensal strains are designated as PA and CO respectively, whereas those strains, whose virulence potential are yet to be elucidated, are considered as uncharacterized (UC) (Table 1).

A total of 108520 annotated CDSs of 36 *E. faecalis* genomes, when clustered by OrthoMCL, yielded a pan-genome of 7131 distinct gene families, which is around 2.4-fold of the average number of annotated CDSs per genome. Among 7131 families, only 2071 (29.04%) are shared by all strains under study, constructing the core genome of *E. faecalis*, another 2049 (28.7%) genes of the pan-genome have been identified as singletons and the rest 3011 (42.2%) are mosaic genes (i.e., genes present in two or more genomes but not in all). Although the core genome of the *E. faecalis* strains represents 68.7% of the average number of genes per genome, when we consider it as a fraction of pan-genome size of the strains considered, it is found to be quite small (29.04%). The small size of the core genome and large number of accessories both concords well with the earlier reports on high level of genome diversity in *E. faecalis*^18^,^19^,^20^. Supplementary Figure 1 shows the distribution of these gene families among *E. faecalis* strains.

**Figure 1:**
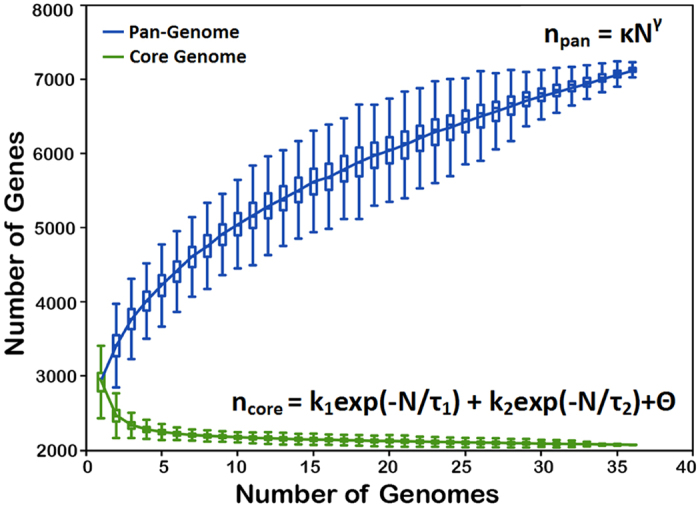
The Core and Pan-Genomes of *E. faecalis.* Number of shared or core genes (green) and total number of genes or pan-genome (blue) curve for 36 different strains of *E. faecalis*. The upper and lower edges of the boxes indicate the first quartile (25th percentile of the data) and third quartile (75th percentile), respectively, of 1000 random different input orders of the genomes. The central horizontal line indicates the sample median (50th percentile). The central vertical lines extend from each box as far as the data extend, to a distance of at most 1.5 interquartile ranges (i.e., the distance between the first and third quartile values). At 36 sequenced genomes, the core-genome had 2071 genes, whereas the pan-genome had 7131 total genes. For Pan-genome equation, n_pan_ is the expected number of genes for a given number of genomes, N is the number of genomes, k and γ are free parameters defined to fit the specific curve. Best fit obtained as variables k and ɣ were determined to be 2,769.13 and 0.26, respectively. Using the Heap’s Law (α = 1 − ɣ), the α value is thus 0.74, which clearly indicates an open Pan-genome. For core genome equation n_core_ is the average of core gene distributions, N is the number of genomes, k_1_, **τ**_1_, k_2_, **τ**_2_ and Θ are free parameters and the inverse square of the interquartile ranges of core genome distributions as weights. The best fit was obtained for asymptotic core genome size Θ as 2065 ±10 (at 95% level of confidence), which fits well with our calculated value of core genome, i.e., 2071 genes.

Figure 1 depicts gradual expansion of the pan-genome and contraction of the core genome of *E. faecalis* with sequential addition of genomes from the dataset. Any possible bias due to the sequential addition of genomes was nullified by random permutations in the order of genome addition. The median were calculated on the size of pan and core genomes after each step and used to generate the pan and core genome curve. The pan-genome size increases steadily without reaching a plateau although 7131 non-redundant gene-families from 36 *E. faecalis* strains were added. Extrapolation of the pan-genome using the power-law regression model based on Heap’s Law^21^,^22^ suggested that *E. faecalis* has an “open” pan-genome expanding at a moderately high rate with α = 0.74.

On the other hand, the core genome appears to have arrived at a “closed” state and the regression analysis for the core genome was performed by fitting a double exponential decay^23^. The best fit was obtained for asymptotic core genome size Θ as 2065 ± 10 (at 95% level of confidence), which fits well with our calculated value of core genome, i.e., 2071 genes, suggesting that further sampling of *E. faecalis* genomes is unlikely to change the estimate of the core genome to a significant extent.

### Phylogenetic analysis of *E. faecalis* strains under study

**Figure 2:**
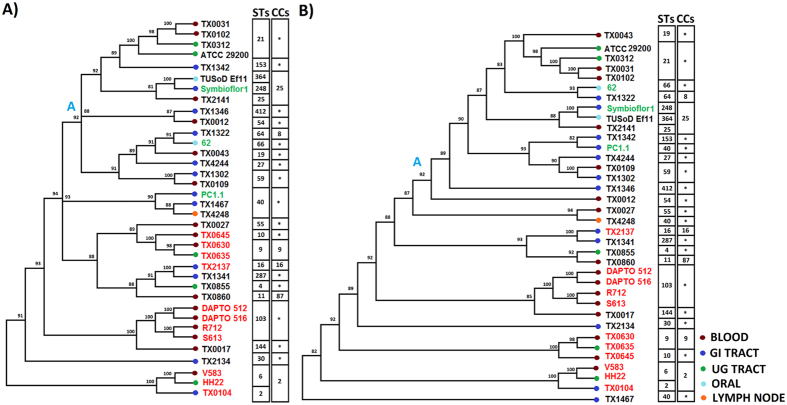
Phylogenetic analysis of *E. faecalis* strains. (**A**) Concatenated Core genome phylogeny. Pathogenic (PA) and Commensal (CO) strains are highlighted in red and green fonts, respectively. (**B**) Pan-genomic tree of binary gene presence/absence data matrix of orthologous gene families. Different color legends are used to separate different strains according to their niches (red: blood, purple: gastrointestinal tract, green: urogenital tract, sky blue: oral and orange: lymph node). Bootstrap support values are also indicated in the tree. Strain types (STs) and Clonal complexes (CCs) determined by *in-silico* MLST analysis are also shown in the figures.

We next wanted to elucidate the evolutionary relationships between the PA, CO and UC strains and for that we constructed two trees: one based on the core genome SNPs (Fig. 2A) and another based on a seven-locus MLST scheme (Supplementary Figure 2). Among 2071 number of core gene clusters, 334 (16%) number of genes were detected to have the recombination signal and these genes were excluded from further analysis. Though in both trees strains from the same STs were clustered together, the PA strains did not segregate under any specific node. There is also substantial incongruence between two trees. For instance, TX0645 co-segregated with TX0630 and TX0635 in the core genome tree (Fig. 2A), but clustered with two different strains in the MLST tree (Supplementary Figure 2). In both cases, the PA strains clustered with some UC strains in the lower parts of the trees, but the sets of UC strains segregating with PA strains are different in two trees. Between two trees, the core genome tree seemed to be more reliable for evolutionary analysis, as three (*gyd, pstS* and *xpt*) of the seven MLST genes showed recombination signatures, which might affect the robustness of the tree.

We further constructed a tree of 36 *E. faecalis* genomes using the pan-matrix (Fig. 2B). This tree should segregate the strains on the basis of their mosaic and unique genes and hence, it might enable identification of the UC strains that were similar to the PA strains in their accessory gene repertoire. Comparison of Fig. 2A and B led to an intriguing observation. If we consider the core genome tree to have two parts – an upper part comprised of the strains under the node A and the lower part comprised of the rest, then among 20 strains of the lower part, 19 strains (11 PA and 8 UC strains) appeared adjacent to one another in the pan-matrix tree also, though the relative positions of these strains differ in two trees. This observation indicated that these strains were close to one another not only from evolutionary point of view, but also in their overall genetic architectures. PC1.1 is the only exception, which shares its sequence type (ST40) with two of these 8 UC strains, TX1467 and TX4248 and co-clustered with them in the lower portion of Fig. 2A, but has dispersed away in Fig. 2B. This is due to a difference in the accessory gene repertoire of PC1.1 and other two strains, as shown later in this report.

Our analysis indicated that 42.2% genes within *E. faecalis* pan genome are mosaic in nature and that might have played a key role in genomic evolution of *E. faecalis*. So we also constructed a tree based only on the mosaic genome binary data matrix, which yielded a phylogenetic tree that is highly congruent with the pan-matrix tree (Fig. 2B) and have higher bootstrap support values (Supplementary Figure 3). The eight UC strains that appeared close to the PA strains in both Fig. 2A and B are presented in orange fonts in this mosaic genome presence/absence matrix tree. As can be seen in Supplementary Figure 3, most of the 19 strains that segregated together in the lower parts of both Fig. 2A and B, co-clustered in this tree also, TX1341 and TX2137 being two exceptions.

As mentioned above, three members of ST40 (PC1.1 – a symbiotic strain, TX1467 and TX4248) have dispersed away from one another in both the pan-matrix and accessory matrix based trees, while the members of all other clonal complexes and sequence types such as CC2, CC25, ST59 and ST103 remained co-clustered. This observation inspired us to delineate the cross-strain divergences in gene repertoire in ST40 that appears to be an ideal candidate for studying evolution of accessory genomes. As depicted in Supplementary Figure 4, three members of ST40 members share a core genome composed of 2431 gene families, but there exists a remarkable difference between the mosaic gene content of the CO strain PC1.1 and those of two UC strains TX1467 and TX4248. The total mosaic genome of these three strains is composed of 394 gene families, most of which (87.6%) are shared only between TX1467 and TX4248. When we looked carefully, we found that this shared region between TX1467 and TX4248 contains the *E. faecalis* pathogenicity island (PAI)^14^,^15^, a prophage region (20 genes) and some uncharacterized genes (Supplementary Figure 4). It appears that it might be due to the presence of PAI and the other commonly shared accessory genes that TX1467 and TX4248 were segregated away from PC1.1 and came closer to PA strains in Fig. 2B and Supplementary Figure 3. Strains from ST40 group is known to be associated with endemic to a restricted geographic region^24^, thus this event of HGT might play a major role for their pathogenicity. Here raises the question: what is the status of PAI in other UC strains?

### Assessment of the pathogenic potential of UC strains on the basis of Pathogenic Island (PAI) gene contents - Identification of eight strains as potential pathogens

**Figure 3:**
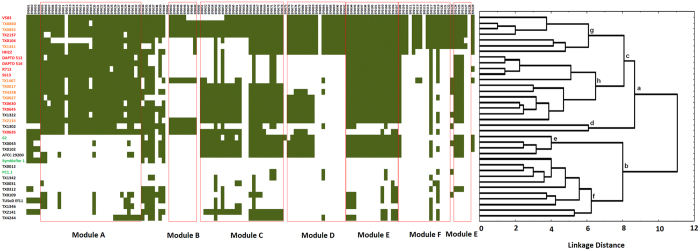
Distribution of pathogenic island (PAI) genes in *E. faecalis* strains. PAI of *E. faecalis* comprises of 129 genes, which are clubbed into six island modules (module A–F, indicated by red boxes). Presence and/or absence of PAI genes are shown in green and white, respectively. Strains are hierarchically clustered according to their PAI module completion. Different nodes in this cluster are shown in lower alphabets (a–h). PA and CO strains are highlighted in red and green fonts, respectively. Putative PA strains are highlighted in orange fonts.

In order to determine the repertoire of PAI genes in *E. faecalis* strains under study, a binary gene presence-absence matrix has been developed based upon BLASTP hit results (Fig. 3). A cluster analysis based on the matrix shows segregation of 36 strains into two distinct groups - the upper cluster under the node “a” contains 21 strains - the same 19 strains that co-segregated in the lower half of Fig. 2A and B along with two other UC strains TX1322 and TX1302. The lower cluster under the node “b” contains three established commensals and 12 remaining UC strains of the dataset. Most of the strains in the upper cluster (under node “a”) are enriched in PAI genes, while the strains in the lower cluster, especially those under node “f”, are devoid of majority of the PAI genes. There are a total 129 number of genes present in the PAI and the upper cluster genomes on average contain 62.0 ± 17.9% of those genes whereas the lower cluster genomes carry on average 17.8 ± 16.1% of those genes.

Co-segregation of eight UC strains – TX0017, TX0027, TX0855, TX0860, TX1341, TX1467, TX2134 and TX4248- in PAI-based clustering as well as in pan-matrix phylogeny suggested that these strains resemble the established PA strains both in their PAI module occurrences and in total (core plus accessory) gene content. These strains, therefore, may be pathogenic in nature and henceforth we will refer to them as putative pathogens (putative PA). Assemblage of established and putative PA strains in the lower part of core genome based phylogeny (Fig. 2A) advocates for possible existence of two distinct evolutionary trajectories for pathogenic and non-pathogenic *E. faecalis*, in general.

Among 15 PAI-deficient *E. faecalis* strains converged under node “b” in Fig. 3, three strains - *E. faecalis* 62, PC1.1 and Symbioflor 1 - have already been characterized as commensal^25^,^26^ and the virulent/benign phenotype of other 12 strains are yet to be characterized. All these 12 UC strains have converged together along with three commensals under a node A of the pan-matrix tree (Fig. 2B), indicating that the overall gene contents of these 12 UC strains are similar to those of the commensals. These strains with PAI-deficient genomes are, therefore, likely to be benign in nature, provided they do not contain any plasmid-borne PAI modules.

There are only two exceptions, TX1302 and TX1322. These two strains bunch with the PAI-enriched strains (i.e., established and putative pathogens) under the node ‘a’ in Fig. 3, but with PAI-deficient strains (i.e., established and putative commensals) under the node “A” in the pan-matrix based tree (Fig. 2B). It is not possible to predict the pathogenic nature of these two strains. It is worth noting that the strain TX1302, though co-segregated with PAI-enriched strains, lacks in certain genes of module A and most of the genes of modules C, E and F (Fig. 3).

### Module A of PAI may be the major discriminating factors between PAI-enriched and PAI-deficient strains

Distribution of different PAI modules among *E. faecalis* strains in two clusters (Fig. 3) suggested that the module A may be the prime discriminating factor between the two clusters. Among six modules A to F, A is the only module that is almost fully present in most of the strains in the upper cluster (average module completion 88.2 ± 9.2%), but hardly occurs in strains of the lower cluster (average module completion 8.9 ± 10.4%). The known commensal strain *E. faecalis* 62 shows close resemblance to the established pathogens like TX0630, TX0635 or TX0640 in the content of modules B-F, but differs substantially in the content of module A. Content of the other modules also vary significantly across different strains, but these differences do not correlate with the distribution of the strains in two clusters under nodes ‘a’ and ‘b’. For instance, a major fraction of the PAI-enriched strains including the established PA strains like H22, DAPTO 512, DAPTO 516, R712 and S673 lack in most of the genes of the modules B, C, D and F, suggesting that the presence of these modules are not essential for *E. faecalis* virulence. On the contrary, most of the genes of the module E exist not only in all strains of the upper cluster, but also in the four strains under the node “e”, which include the established commensal *E. faecalis* 62, indicating that the mere presence of the module E does not ensure the pathogenic phenotypes of an *E. faecalis* strain. All these observations suggest that among six different PAI modules, the module A might play a crucial role in *E. faecalis* pathogenesis.

### Four genes of the module A are present exclusively in reported and putative PA strains – novel drug targets

In an attempt to identify hitherto unknown pathogen-specific genes in *E. faecalis*, if any, we have carried out a comparative study of genes that are present in the PA and putative PA strains, but not in the known symbionts of the current dataset. Nineteen genes are found to be present in all of the eleven pathogenic strains, but not in any of the three known commensals (Supplementary Table 1). Protein function characterization revealed that most of the pathogen-specific gene-products are hypothetical. COG analysis of these gene-products reveals that two of the proteins, EF0500 and EF0503, belong to Intracellular trafficking, secretion, and vesicular transport, some (EF0499 and EF0511) in Replication, recombination and repair etc, but COGs classifier could not describe most of these proteins.

Interestingly enough, we have found a stretch of 7.4 kb region composed of four genes (highlighted in Supplementary Table 1) that is present in all established and putative PA strains and also in two other PAI-enriched strains TX1302 and TX1322, but absent in 3 established CO strains and in other putatively benign UC strains. Three of these proteins are annotated as hypothetical proteins and the fourth one is a lipoprotein. In order to characterize their functions we have used BLASTp^27^ search against entire non-redundant database with a moderately stringent criteria (>50% identity and coverage). Two of them (GI: 29375128 and 29375131) found to have homologous parts with the type VI secretion system, - a well known mechanism in gram negative bacteria for injecting effectors proteins and virulent factors^28^. One protein (GI: 29375130) is homologous with the Plasmodium parasites MAEBL (Merozoite apical erythrocyte-binding ligand) family membrane protein which is believed to facilitate merozoite entry into the host erythrocyte^29^. The fourth protein (GI: 29375129) is a conserved lipoprotein in *E. faecalis* strains. None of these proteins are found to be present in any known symbiotic *E. faecalis* strains when compared by sequenced homology (BLASTp search). This genomic region containing these proteins is a part of the module A and possibly integrated plasmid region of PAI^30^. The sequences of these four proteins are well conserved in all established and putative pathogenic genomes (Supplementary Figure 5).

This region is found to be homologous to parts of other virulent plasmids such as sex pheromone plasmids (pAD1, pTEF1 and pAM373), which have been long studied due to its pheromone-inducible conjugation behavior as well as its contribution to virulence^21^,^22^,^23^,^24^,^25^,^26^,^27^,^28^,^29^,^30^,^31^,^32^,^33^. It is tempting to speculate that some, if not all, of these genes might have a contribution to *E. faecalis* pathogenesis. However, validation of this hypothesis would require functional characterization of these genes through experimental studies.

Since *E. faecalis* is a member of normal human flora and only some of its strains, not all, are pathogenic, therapeutic measures against this organism should be designed in a way to target specifically the pathogenic strains only. This stretch of four genes, being present only in established and putative pathogenic *E. faecalis*, but not in their commensal counterparts, their products could serve as potential drug targets.

It is well known that presence of intrinsically disordered regions (IDR) in a protein may enhance its potency as a drug target^34^. Intrinsically Disordered Proteins (IDPs) or proteins with IDR can modulate various biological activities owing to lack of specific structures and high accessibility of their polypeptide chains through different post-translational modifications, such as phosphorylation, acetylation, ubiquitination etc^35^,^36^. IDPs are frequently found in pathogenic microbes^37^. To find out whether any of the four pathogen-specific proteins in *E. faecalis* contain IDRs, we have carried out IDR prediction analysis in those sequences.

**Figure 4:**
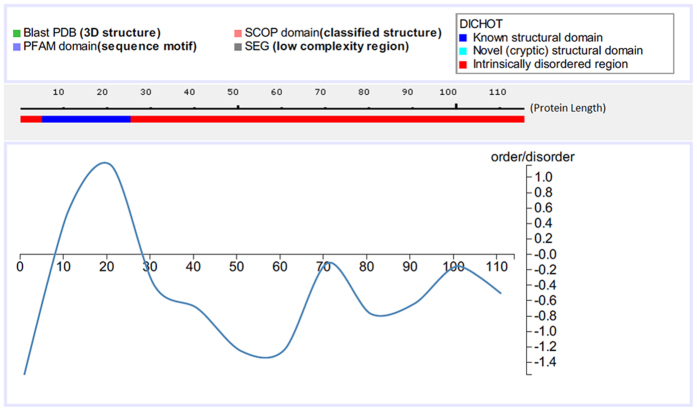
Identification of Intrinsically Disordered Regions (IDRs) in pathogen specific protein of *E. faecalis.* IDRs are identified by sequence comparison with various structural features in DICHOT tool of IDEAL (Intrinsically Disordered proteins with Extensive Annotations and Literature) database. The selected features are i) PDB Blast for 3D structure ii) PFAM domain search for sequence motif iii) SCOP domain search for classified structure and iv) SEG analysis for low complexity region identification. Final order-disorder regions are shown in line diagram along the length of protein.

The extents of IDRs vary across these four proteins. For example, Protein EF0501, that code for lipoprotein, has long C-terminal id regions in their sequence (Fig. 4). Among other three pathogen specific proteins, protein EF0502 and EF0503 have ID regions throughout their sequence length whereas protein EF0500 has no observable ID regions (Supplementary Figure 6). These observations indicate that EF0502 and EF0503 have strong potential as drug targets. It would have been great to explore the role of the predicted IDRs in *E. faecalis* pathogenesis, if any, but this is beyond the scope of the present study.

### Trends in occurrences of virulence factors in *E. faecalis* plasmids

At this point one may argue that occurrences of PAI genes and other virulence factors within the chromosomal sequences could be the sufficient, but not necessary criterion for an *E. faecalis* strain to be pathogenic. One cannot rule out the possibility that an *E. faecalis* genome, which appeared to be deficient in PAI modules and other virulence factors and hence, predicted to be benign, may turn virulent, owing to plasmid-borne virulence factors. Hence, for complete assessment of pathogenic potential, presence/absence of virulence factors in the plasmid sequences of the respective strains also need to be considered. Unfortunately, no information on plasmid sequences is available for most of the *E. faecalis* strains under study. To date, twenty seven plasmid sequences of fourteen *E. faecalis* strains are available in the public domain. A BLASTP search (with threshold identity >50%, length coverage >50% coverage and e-value < 1e-05) of eleven major *Enterococcal* virulent factors found in Virulence Factor Database (VFDB)^38^, identified very few of them to be present in all the plasmid sequences (Supplementary Table 2). Aggression substance (AS) that contributes to pathogenicity by enhancing cell adhesion and internalization into enterocytes^39^,^40^ are found in ten plasmid sequences. Beside these, other virulent factors are markedly absent in all of the plasmid sequences. These findings indicated seldom occurrences of virulence factors in the plasmid sequences of *E. faecalis*. However, the possibility that the fourteen PAI-deficient UC strains, which have been predicted here as putative commensal strains, may have plasmid-borne virulence factors cannot be ruled out, till sequences of all plasmids from these strains are determined and analyzed.

### Exclusive genes in *E. faecalis* genomes derived from specific body-sites of human hosts

Neither the core genome based phylogeny (Fig. 2A) nor the pan-matrix based clustering (Fig. 2B) exhibited any general trend of host body-site-specific segregation of *E. faecalis* isolates, though there were some examples of co-clustering of two or more genomes isolated from specific body niches. For instance, three GIT isolates PC1.1, TX1342 and TX4244 were quite away from one another in concatenated core genome tree (Fig. 2A), implying their evolutionary remoteness. But in the pan-matrix based clustering (Fig. 2B), the three GIT isolates co-segregated indicating a similarity in their accessory gene complements. These observations pointed towards niche-specific convergence of distant strains to similar gene repertoire. A question arises at this point: could there be any host body-site specific genes in these human microbiome components?

To address this issue, we have searched for the genes present exclusively in the *E. faecalis* genomes derived from a particular host body site and found 215, 129, 4 and 1 genes to be exclusive to the blood, GIT, UGT, oral and lymph node isolates, respectively (Supplementary Table 3). The G+C-content of these genes, in most cases, differ substantially from the average G+C-content of the respective genomes (Supplementary Figure 7), which suggests that a major portion, if not all, of the niche-specific genes might have been acquired by through HGT.

**Figure 5:**
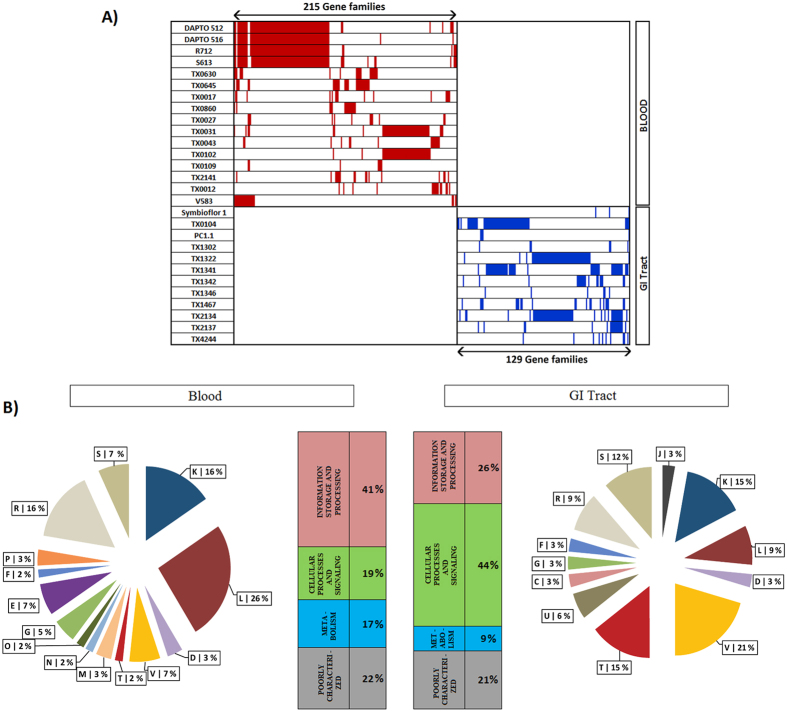
Specific genes among Blood and GI Tract niches in *E. faecalis* strains. (**A**) 215 and 129 gene families specific to Blood and GI Tract niches are shown in red and purple color, respectively. (**B**) Comparative functional analysis of Cluster of Orthologous Groups (COGs) frequencies between Blood and GI Tract niches. COG functional categories represented by one letter code are- J: Translation, ribosomal structure and biogenesis, K: Transcription, L: Replication, recombination and repair, D: Cell cycle control, cell division, chromosome partitioning, V: Defense mechanisms, T: Signal transduction mechanisms, M: Cell wall/membrane/envelope biogenesis, N: Cell motility, U: Intracellular trafficking, secretion, and vesicular transport, O: Posttranslational modification, protein turnover, chaperones, C: Energy production and conversion, G: Carbohydrate transport and metabolism, E: Amino acid transport and metabolism, F: Nucleotide transport and metabolism, H: Coenzyme transport and metabolism, I: Lipid transport and metabolism, P: Inorganic ion transport and metabolism, Q: Secondary metabolites biosynthesis, transport and catabolism, R: General function prediction only, S: Function unknown. Frequencies of four major COG groups are also shown in these two niches.

Further analyses revealed significantly different (p < 0.05) niche-specific trends in distribution of certain COG categories among these genes. For instance, among the GIT-specific genes (i.e., genes specific to GIT-derived *E. faecalis* genomes), 44% belong to the Cellular processes and signaling category and 26% to Information processing, while among the blood-specific genes, 41% pertain to Information processing and only 19% belong to Cellular processes and signaling (Fig. 5). An examination to the further details revealed that genes belong to Replication, recombination & repair category are significantly abundant in the blood-specific isolates (26%) as compared to that in GIT-specific ones (9%) (p = 0.001). While the percentage of exclusive genes involved in defense and signal transduction are significantly higher among the GIT-derived *E. faecalis* (21% and 15% for defense and signal transduction respectively) than among their blood-derived counterparts (7% and 2% for defense and signal transduction respectively) (p = 0.007) (Fig. 5). These observations indicate that *E. faecalis* might have generated distinct genetic strategies for adaptation to different body-sites of the human hosts through selective, niche-specific acquisition of genes, as observed earlier in case of *Prevotella*^41^.

## Conclusion

The pan-genome of *E. faecalis*, constructed on the basis of 36 genomes, is still open, though the core genome seems to have reached almost a closed state. The small size of the core genome and a large number of accessory genes support the notion of genomic fluidity of *E. faecalis*^42^. In previous studies, routinely used MLST analysis could not properly distinguish pathogenic and non-pathogenic strains of *E. faecalis*, but here we have shown that recombination free core genome and pan-genome phylogeny reveal evolutionary segregation of these strains. On the basis of the core genome SNP based phylogeny, pan-matrix based clustering and PAI modules content, 8 uncharacterized *E. faecalis* strains have shown potential of being pathogenic in nature. Among six modules of PAI, module A appears to be the major discriminating factors between PAI-enriched and PAI-deficient strains. We have identified four genes in the module A of *E. faecalis* PAI, which exist in all established and putative pathogenic strains, but are absent in three reported commensals and in most of the other PAI-deficient strains. These genes may be probed in future to explore their role, if any, in *E. faecalis* pathogenesis. Existence of disordered regions in these four proteins suggests that these proteins may serve as potential drug targets for pathogenic *E. faecalis*. Presence of exclusive, habitat-specific genes in the blood, GIT, UGT, oral and lymph node derived *E. faecalis* genomes and distinct trends in distribution of broad COG categories among these genes in GIT and blood indicate that these microbiome components might have developed distinct, habitat-specific genetic strategies for host adaptation.

## Materials and Methods

### Sequence retrieval

Genome and proteome sequences of all *E. faecalis* strains, for which the host body sites are documented in the HMP catalogue were retrieved from HMP database (hmpdacc.org). 33 such strains were available at the time of initiation of this study. Sequences of 3 more completely sequenced strains were also downloaded from the NCBI database.

### Orthologs retrieval

All annotated protein sequences from the 36 *E. faecalis* genomes under study were subjected to orthology prediction using OrthoMCL^43^. This program first makes an all-against-all BLASTp^27^, and then defines putative clusters of orthologs or recent paralogs based on reciprocal BLAST hits using a Markov Cluster algorithm (MCL). In excess, only the reciprocal BLAST hits showing >50% identity and 50% coverage with each other were considered as orthologs. The paralogs were removed from all gene families. This resulted in a binary pan-matrix, where, rows represent specific gene clusters (also called gene families) and columns represent genomes in ‘1’/‘0’ format (indicating presence/absence of a specific gene family in the respective genome).

### Pan-genome extrapolation

This binary pan-matrix was then processed by a perl script, written in-house, for extraction of the core and dispensable genome of 36 *E. faecalis* strains under study. This script fetches the gene identifiers in each cluster and matches it with the Pan-genomic sequences of *E. faecalis* strain which is already being generated as a FASTA database. The core and dispensable clusters are fetched separately and the protein FASTA sequences are extracted in different files.

### Phylogenetic tree construction

Both sequence based and gene content based phylogenetic trees for the organisms under study were constructed for understanding the interrelation between them. Sequence based phylogenetic approaches which are used are MLST and core genome phylogeny. *In-silico* Multi locus sequence typing (MLST) was used to determine the sequence types (STs) and clonal complex (CC) structures of each strain against *E. faecalis* MLST scheme (efaecalis.mlst.net/). For this study, allelic profiles of seven housekeeping genes*: gdh*, gyd, pstS, gki, aroE, xpt, and yiqL (total 3,297 bp) downloaded from MLST database. To assess the evolutionary relationship of *E. faecalis* strains, these gene sequences were concatenated for each strain and used to construct a maximum likelihood (ML) phylogenetic tree using MEGA 5.0^44^. Initially, the best fit nucleotide substitution model of ML analysis was assessed. The Hasegawa-Kishino-Yano (HKY) model with a discrete gamma distribution (+G) for prediction of the evolutionary rate differences among sites and a presumption on evolutionary invariance of certain fraction of sites (+I) was found to be the best fit with the lowest Bayesian Information Criterion (BIC) scores. The statistical significance of the tree was assessed with 100 bootstrap replicates.

For core genome phylogeny, all the core genes were further analyzed to determine their sequencing quality. All the genes containing paralogous sequences are excluded from this analysis. Nucleotide and amino acid sequences were aligned using MUSCLE software^45^. The protein alignment of each gene sequence was reverse aligned with the corresponding codon alignment and genes having internal stop codon and/or alignment inconsistency are excluded from this study. We also use Maxchi^46^ and Phylpro^47^ recombination tests to determine recombination signature in those genes and those showing evidence of recombination are excluded from our analysis. Finally, after all those refinement, we got 1737 core genome sequences. The amino acid sequences of each of these genes are concatenated and aligned, and sites with gaps were deleted using trimAl^48^. A maximum likelihood phylogenetic tree was constructed using RAxML 8^49^ with 100 bootstrap replicate using the PROTGAMMA algorithm.

The pan-matrix i.e., the binary matrix of presence/absence of genes was used to construct Pan-genomic tree using PAST^50^. Single linkage Euclidean clustering was used for the same and the statistical significance of the branches was assessed with 100 bootstrap replicates.

### Analysis of Clusters of Orthologous Groups (COG) of proteins

The core, mosaic and unique genes of 36 *E. faecalis* strains were assigned to 20 different COG categories. COGs were annotated by performing RPSBLAST of each protein sequence against NCBI’s COG database^51^ with an E-value cutoff <0.001 in WEBMGA server^52^. Statistical significance of the distribution of COG categories was measured by nonparametric chi-square analysis (in a 2 × 2 contingency table) using STATISTICA (Version 6.0)^53^.

### Determining pathogenicity islands (PAI)

To study the distribution of PAI modules in different *E. faecalis* strains, the 143 kb long PAI sequence of the virulent strain MMH34 was downloaded from PAIDB^54^. Pathogenicity island of *E. faecalis* is a 143 kb long stretch of bases encoding 129 proteins. The homology conservation of this 143 kb sequence with different strains were analyzed with a suffix array based heuristic dot plot computation tool Gepard^55^, with a default word length of 10 nucleotides. The aligned region were then extracted and further analyzed for the presence of 129 PAI-encoded proteins with a BLASTP cutoff of <1e^−10^. For strains bearing plasmids in their genomes, PAI proteins were also searched in them.

### Identification of Intrinsically Disordered Regions (IDRs)

IDRs in proteins were predicted by comparing them against databases of known disordered proteins in IDEAL^56^ database. Here, we use DICHOT prediction tool for predicting disordered regions in our proteins in IDEAL protein repertoire^57^. We considered a protein as IDP if it has a stretch of at least 30 consecutive disordered residues following by the recommendations of the Dunker *et al*.^58^.

## Additional Information

**How to cite this article**: Bakshi, U. *et al*. Assessment of virulence potential of uncharacterized *Enterococcus faecalis* strains using pan genomic approach – Identification of pathogen–specific and habitat-specific genes. *Sci. Rep.* **6**, 38648; doi: 10.1038/srep38648 (2016).

**Publisher's note:** Springer Nature remains neutral with regard to jurisdictional claims in published maps and institutional affiliations.

## Supplementary Material

Supplementary Files

## Figures and Tables

**Table 1:** Details of *Enterococcus faecalis* strains under study.

No.	Organism Name	Isolation site	Accession	Size (Mb)	GC%	CDS count	Pathogenic or commensal^*^	STs
1	*E. faecalis V583*	Blood	PRJNA54927	3.3	37.3	3112	PA	6
2	*E. faecalis 62*	Oral	PRJNA159663	3.1	37.4	2897	CO	66
3	*E. faecalis str. Symbioflor 1*	GI Tract	PRJNA183342	2.8	37.7	2733	CO	248
4	*E. faecalis DAPTO 512*	Blood	PRJNA60591	3.1	37.3	3104	PA	103
5	*E. faecalis DAPTO 516*	Blood	PRJNA60619	3.1	37.3	3100	PA	103
6	*E. faecalis R712*	Blood	PRJNA46983	3.0	37.3	3086	PA	103
7	*E. faecalis S613*	Blood	PRJNA46987	3.0	37.3	3096	PA	103
8	*E. faecalis TX0012*	Blood	PRJNA47145	2.8	37.4	2743	UC	NA
9	*E. faecalis TX0017*	Blood	PRJNA47147	3.0	37.3	3074	UC	144
10	*E. faecalis TX0027*	Blood	PRJNA47151	3.1	37.2	3124	UC	55
11	*E. faecalis TX0031*	Blood	PRJNA47153	2.8	37.5	2759	UC	21
12	*E. faecalis TX0043*	Blood	PRJNA47155	2.8	37.5	2838	UC	19
13	*E. faecalis TX0102*	Blood	PRJNA60609	2.9	37.3	2831	UC	21
14	*E. faecalis TX0109*	Blood	PRJNA52591	3.0	37.4	2987	UC	59
15	*E. faecalis TX0630*	Blood	PRJNA47171	3.2	37	3411	PA	9
16	*E. faecalis TX0645*	Blood	PRJNA47175	3.2	37.1	3278	PA	10
17	*E. faecalis TX0860*	Blood	PRJNA52589	3.1	37.1	3114	UC	11
18	*E. faecalis TX2141*	Blood	PRJNA47131	2.9	37.5	2970	UC	25
19	*E. faecalis PC1.1*	GI Tract	PRJNA47777	2.8	37.5	2619	CO	40
20	*E. faecalis TX0104*	GI Tract	PRJNA55351	3.1	37.3	3273	PA	2
21	*E. faecalis TX1302*	GI Tract	PRJNA47181	2.9	37.5	2853	UC	59
22	*E. faecalis TX1322*	GI Tract	PRJNA55479	2.9	37.3	3006	UC	64
23	*E. faecalis TX1341*	GI Tract	PRJNA47183	3.0	37.2	3035	UC	287
24	*E. faecalis TX1342*	GI Tract	PRJNA47185	2.8	37.5	2793	UC	NA
25	*E. faecalis TX1346*	GI Tract	PRJNA47187	2.8	37.6	2827	UC	412
26	*E. faecalis TX1467*	GI Tract	PRJNA47189	3.0	37.1	3510	UC	40
27	*E. faecalis TX2134*	GI Tract	PRJNA52587	3.1	37.1	3196	UC	30
28	*E. faecalis TX2137*	GI Tract	PRJNA47129	3.0	37.2	3000	PA	16
29	*E. faecalis TX4244*	GI Tract	PRJNA47141	2.9	37.3	2902	UC	27
30	*E. faecalis ATCC 29200*	UG Tract	PRJNA55477	2.9	37.5	2955	UC	21
31	*E. faecalis HH22*	UG Tract	PRJNA55475	3.0	37.2	3227	PA	6
32	*E. faecalis TX0312*	UG Tract	PRJNA47165	2.8	37.6	2789	UC	21
33	*E. faecalis TX0635*	UG Tract	PRJNA60589	3.2	37.2	3263	PA	9
34	*E. faecalis TX0855*	UG Tract	PRJNA52585	3.0	37.2	3032	UC	4
35	*E. faecalis TX4248*	Lymph Node	PRJNA52583	3.2	37.1	3278	UC	40
36	*E. faecalis TUSoD Ef11*	Oral	PRJNA55455	2.8	37.7	2705	UC	364

*PA = Pathogenic CO = Commensal UC = Uncharacterized.

NOTE.—References indicating their Pathogenic and Commensal nature are listed in Supplementary Table 4. The first three genomes are completely sequenced.
