# Supplementary material for: Assessment of virulence potential of uncharacterized Enterococcus faecalis strains using pan genomic approach – Identification of pathogen–specific and habitat-specific genes
Source: Sci Rep. 2016 Dec 7;6:38648. doi: 10.1038/srep38648 (PMC5141418; doi:10.1038/srep38648)
Supplement: Supplementary Files [file srep38648-s1.pdf]

Assessment of virulence potential of uncharacterized Enterococcus faecalis strains using pan genomic approach – Identification of pathogen–specific and habitat-specific genes

Utpal Bakshi[1, 2], Munmun Sarkar[1, 3], Sandip Paul[1] & Chitra Dutta[1, 2,*]

[1]Structural Biology & Bioinformatics Division, CSIR- Indian Institute of Chemical Biology, 4, Raja S. C. Mullick Road, Kolkata 700032, India.

[2]Academy of Scientific and Innovative Research (AcSIR), CSIR- Indian Institute of Chemical Biology (CSIR-IICB), 4, Raja S. C. Mullick Road, Kolkata 700032, India

[3]Present address: MedGenome Labs Pvt. Ltd., Narayana Health City, Bommasandra, Hosur Road, Bangalore - 560 099, India.

[*]Corresponding Author, C.D. (cdutta@iicb.res.in).

	Core genes	Dispensable genes
Number of Gene families	2071	5060

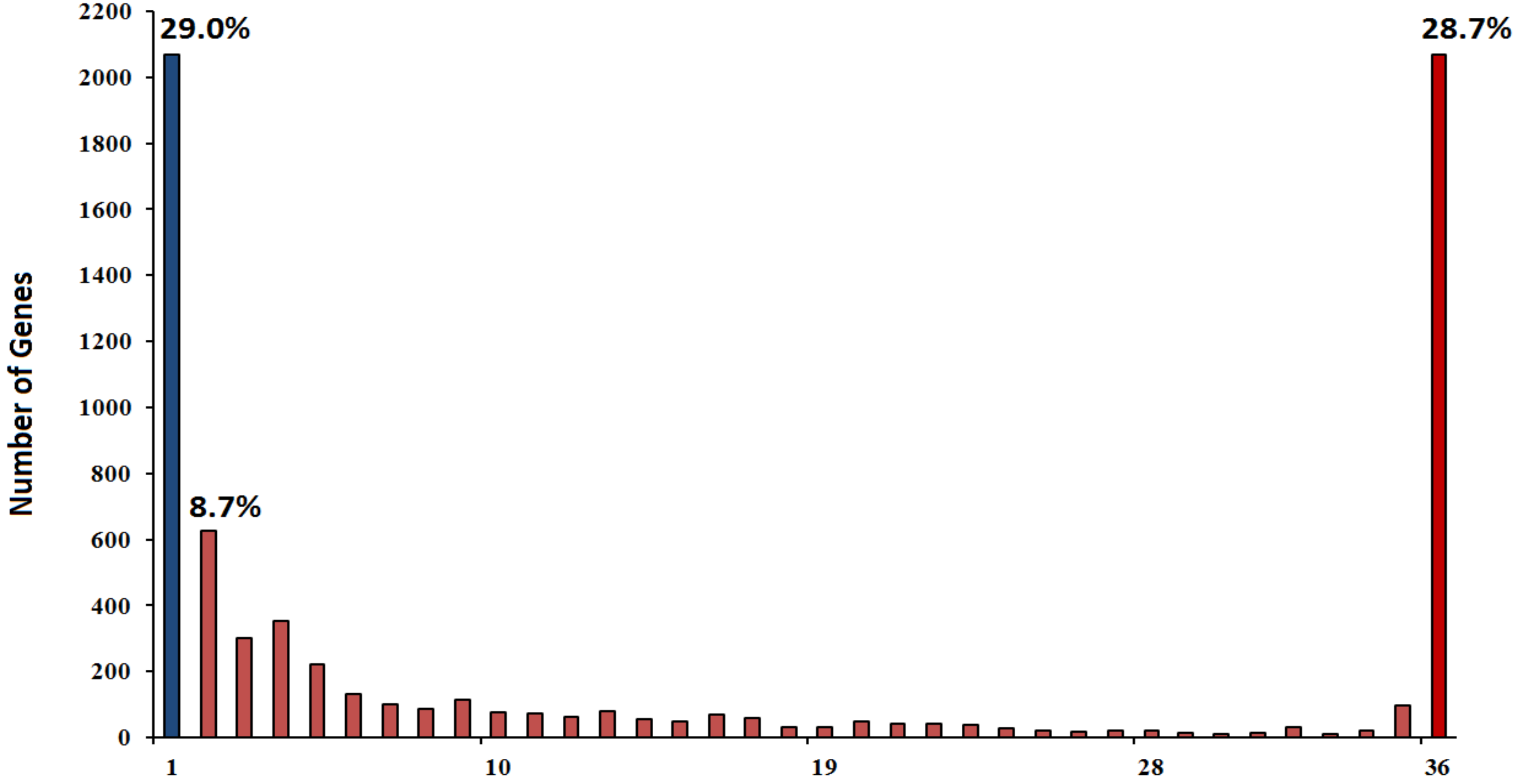

Figure S1: Gene family distribution within 36 genomes of *E.faecalis.*

Core and Dispensable genes are colored as blue and red respectively. Among Dispensable genome, genes shared between two genomes (8.7%) and shared among none (singletons, which are 28.7%) are shown in the plot.

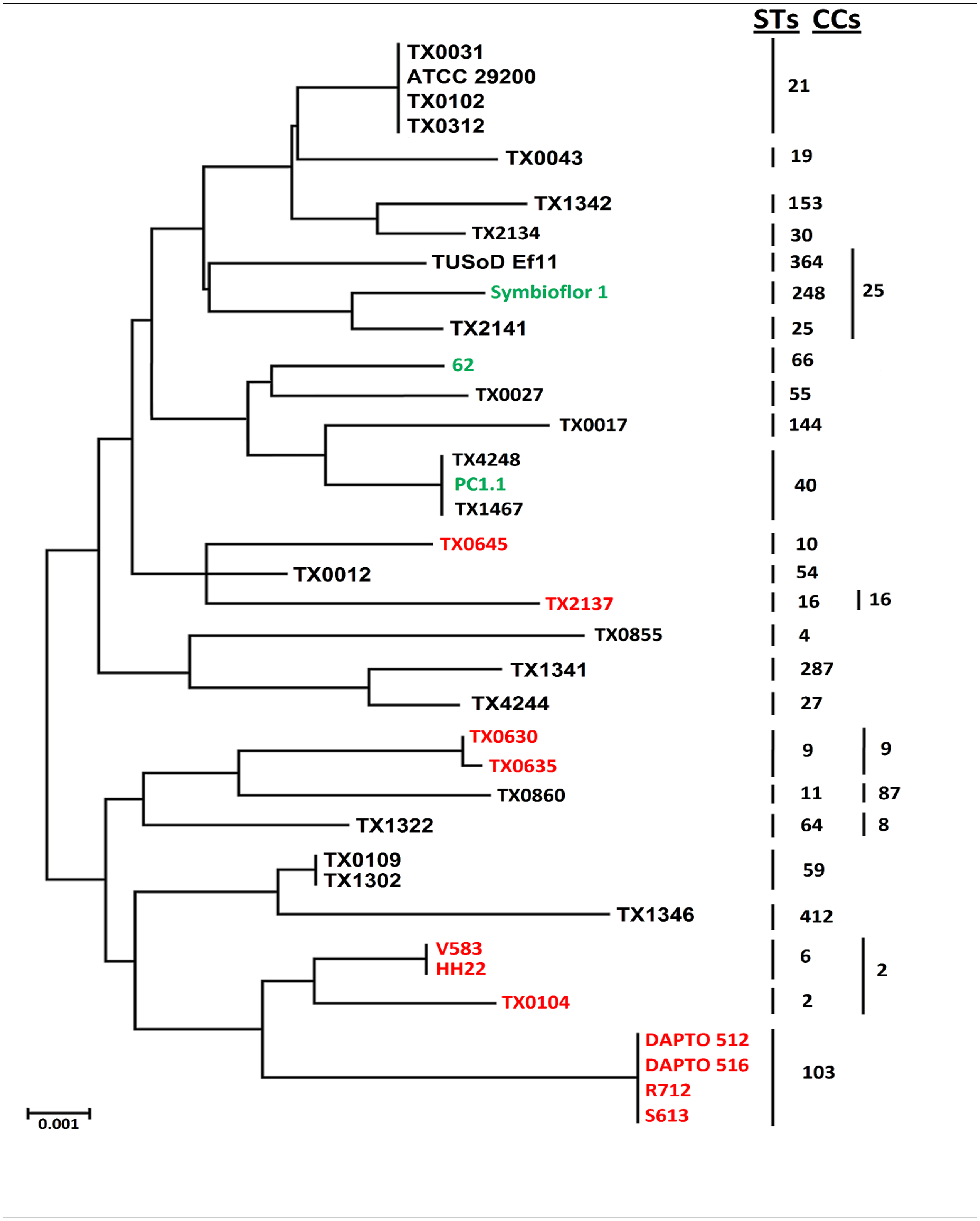

Figure S2 – Maximum Likelihood Phylogenetic tree of concatenated sequences of seven housekeeping genes of *E.faecalis* for in-silico Multilocus sequence typing (MLST) analysis. The housekeeping genes used in MLST selected for analysis were: *gdh* (glucose-6-phosphate dehydrogenase), *gyd* (glyceraldehyde-3-phosphate dehydrogenase), *pstS* (phosphate ATP binding cassette transporter), *gki* (putative glucokinase), *aroE* (shikimate 5-dehydrogenase), *xpt* (shikimate 5-dehydrogenase), and *yiqL* (acetyl-coenzyme A acetyltransferase). Strain types (STs) and Clonal complexes (CCs) are also shown in the figure. Pathogenic strains are highlighted in red fonts and Commensals are highlighted in green fonts.

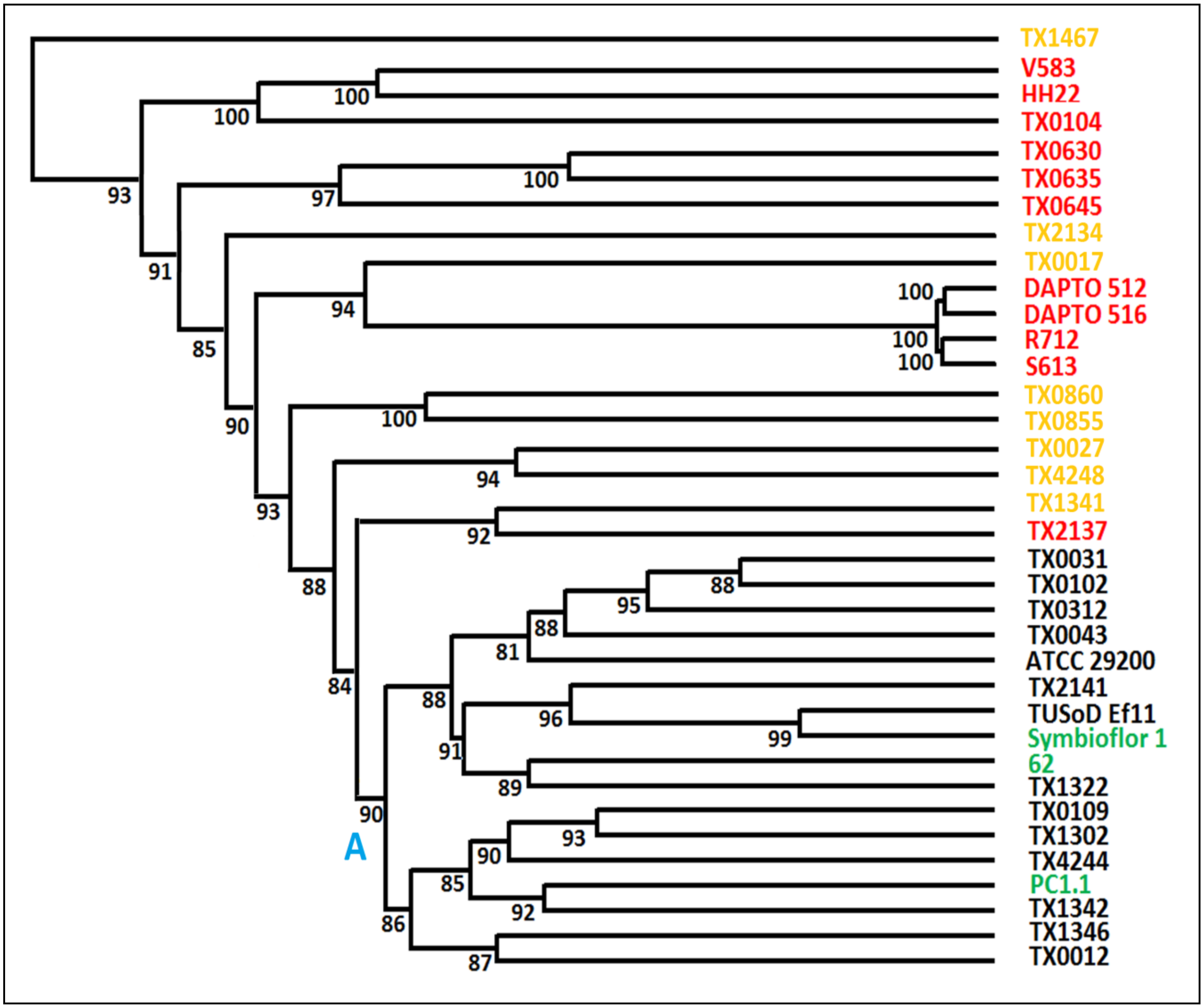

Figure S3 – Phylogenetic tree based on mosaic genome presence/absence data matrix. Pathogens (PA) and Commensal (CO) strains are represented in red and green fonts, respectively. Uncharacterized (UC) strains that appeared with PA strains previously in both core genome and pan-matrix phylogeny are presented in orange fonts. Bootstrap values are also shown in the tree.

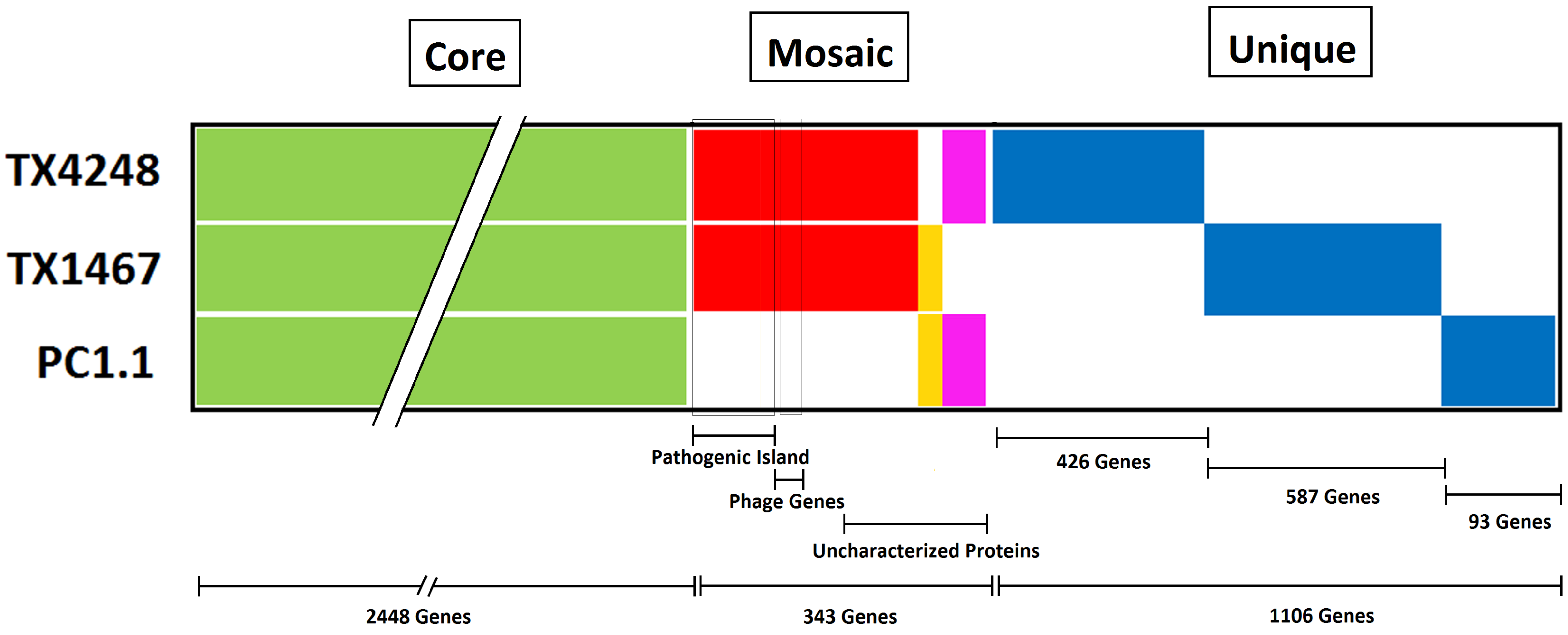

Figure S4 – Distribution of the core and accessory genomes of three ST40 strains. Core (green), unique (blue) and mosaic (red, yellow and pink) are shown for each of the strains. Pathogenic island (PAI) and phage regions in the mosaic genome are marked.

1) Hypothetical protein (EF502) of *E.faecalis* V583:

```
DAPTO_516      ------------------------------------------------------------
TX1467         MNKKLLIKTALLFLVGCFFVVTQTTSVNAAPKPDENKVIVTEPTTNVDTIYDTYKDNAFE
TX0104         MNKKLLIKTALLFLVGCFFVVTQTTSVNAAPKPDENKVIVTEPTTNVDTIYDTYKDNAFE
TX0027         MNKKSLVKAVLLFLVGCFFVFTQTTAVNAAPKPDENKVIVTEPTTNVDTIYDTYKDNAFE
TX0630         MNKKSLVKAVLLFLVGCFFVFTQTAAVNAAPKPDENKVIVTEP-----------------
TX1341         MNKKSLVKAVLLFLVGCFFVFTQTAAVNAAPKPDENKVIVTEPTTNVDTIYDTYKDNAFE
TX0017         MNKKLLIKTALLFLVGCFFVVTQTTSVNAAPKPDENKVIVTEPTTNVDTIYDTYKDNAFE
TX0645         MNKKLLIKTALLFLVGCFFVVTQTTSVNAAPKPDENKVIVTEPTTNVDTIYDTYKDNAFE
TX0635         ------------------------------------------------------------
TX0860         MNKKLLIKTALLFLVGCFFVVTQTTSVNAAPKPDENKVIVTEPTTNVDTIYDTYKDNAFE
TX0855         ------------------------------------------------------------
TX4248         MNKKLLIKTALLFLVGCFFVVTQTTSVNAAPKPDENKVIVTEPTTNVDTIYDTYKDNAFE
TX2134         MDKKSLIKAALLLLVGCFFVFTQTTSVNAAPKPDENKVIVTEPTTNVDTIYDTYKDNAFE
V583           MDKKSLIKAALLLLVGCFFVFTQTTSVNAAPKPDENKVIVTEPTTNVDTIYDTYKDNAFE
TX2137         ------------------------------------------------------------
HH22           MNKKLLIKTALLFLVGCFFVVTQTTSVNAAPKPDENKVIVTEPTTNVDTIYDTYKDNAFE
R712           MNKKSLVKAVLLFLVGCFFVFTQTTAVNAEPKPDENKVIVTEPTTNVDTIYDTYKDNAFE
DAPTO_512      -------------------MFTQTTAVNAEPKPDENKVIVTEPTTNVDTIYDTYKDNAFE
S613           -------------------MFTQTTAVNAEPKPDENKVIVTEPTTNVDTIYDTYKDNAFE


DAPTO_516      ------------------------------------------------------------
TX1467         LMTKEKEKE--SVTGFNEAITNASATIKTFVWSMVKGLGEFNAVMVKTLFSMDIITAIKQ
TX0104         LMTKEKEKE-------SEAITNASATIKTFVWSMVKGLGEFNAVMVKTLFSMDIITAIKQ
TX0027         LMTKEKEKDKPWSTGISEAVVNASAAVKTFVWAGVKGLGEFNAVMVKTLFSMDIITAIKQ
TX0630         ---------------------------------------EFNAVMVKTLFSMDIITAIKQ
TX1341         LMTKEKEKDKPWSTGISEAVVNASAAVKTFVWAGVKGLGEFNAVMVKTLFSMDIITAIKQ
TX0017         LMTKEKEKE--SVTGFNEAITNASATIKTFVWSMVKGLGEFNAVMVKTLFSMDIITAIKQ
TX0645         LMTKEKEKE--SVTGFNEAITNASATIKTFVWSMVKGLGEFNAVMVKTLFSMDIITAIKQ
TX0635         ----------------------------------------FNAVMVKTLFSMDIITAIKQ
TX0860         LMTKEKEKE--SVTGFNEAITNASATIKTFVWSMVKGLGEFNAVMVKTLFSMDIITAIKQ
TX0855         ----------------------------------------FNAVMVKTLFSMDIITAIKQ
TX4248         LMTKEKEKE--SVTGFNEAITNASATIKTFVWSMVKGLGEFNAVMVKTLFSMDIITAIKQ
TX2134         LMTKEKEKD--SAFGVKEAITNASAAVKTFVWAGVKGLGEFNAVMVKTLFSMDIITAIKQ
V583           LMTKEKEKD--SAFGVKEAITNASAAVKTFVWAGVKGLGEFNAVMVKTLFSMDIITAIKQ
TX2137         ------------------------------------------------------------
HH22           LMTKEKEKE--SVTGFNEAITNASATIKTFVWSMVKGLGEFNAVMVKTLFSMDIITAIKQ
R712           LMTKEKKQD--SWTGVEEAITNASATIKTFVWSMVKGLGAFNAVMVKTLFSMDIITAIKQ
DAPTO_512      LMTKEKKQD--SWTGVEEAITNASATIKTFVWSMVKGLGAFNAVMVKTLFSMDIITAIKQ
S613           LMTKEKKQD--SWTGVEEAITNASATIKTFVWSMVKGLGAFNAVMVKTLFSMDIITAIKQ


DAPTO_516      ------------------------------------------------------------
TX1467         PIMNLTSSIATNMLGIAGTQVMLIKSV---------------------------------
TX0104         PIMNLTSSIATNMLGIAGTIGIAFVFVILGVKFIGQQRYKR-------------------
TX0027         PIMNLTSSIATNMLGIAGTIGIAFVFVILGVKFIGQQRYKRFFGIFLMTILIFTGLSVLK
TX0630         PIMNLTSSIATNMLGIAGTIGIAFVFVILGVKFIGQQRYKRFFGIFLMTILIFTGLSVLK
TX1341         PIMNLTSSIATNMLGIAGTIGIAFVFVILGVKFIGQQRYKRFFGIFLMTILIFTGLSVLK
TX0017         PIMNLTSSIATNMLGIAGTIGIAFVFVILGVKFIGQQRYKRFFGIFLMTILIFTGLSVLK
TX0645         PIMNLTSSIATNMLGIAGTIGIAFVFVILGVKFIGQQRYKRFFGIFLMTILIFTGLSVLK
TX0635         PIMNLTSSIATNMLGIAGTIGIAFVFVILGVKFIGQQRYKRFFGIFLMTILIFTGLSVLK
TX0860         PIMNLTSSIATNMLGIAGTIGIAFVFVILGVKFIGQQRYKRFFGIFLMTILIFTGLSVLK
TX0855         PIMNLTSSIATNMLGIAGTIGIAFVFVILGVKFIGQQRYKRFFGIFLMTILIFTGLSVLK
TX4248         PIMNLTSSIATNMLGIAGTIGIAFVFVILGVKFIGQQRYKRFFGIFLMTILIFTGLSVLK
```

```
TX2134        PIMNLTSSIATNMLGIAGTIGIAFVFVILGVKFIGQQRYKRFFGIFLMTILIFTGLSVLK
V583          PIMNLTSSIATNMLGIAGTIGIAFVFVILGVKFIGQQRYKRFFGIFLMTILIFTGLSVLK
TX2137        -----------------GTIGIAFVFVILGVKFIGQQRYKRFFGIFLMTILIFTGLSVLK
HH22          PIMNLTSSIATNMLGIAGTIGIAFVFVILGVKFIGQQRYKRFFGIFLMTILIFTGLSVLK
R712          PIMNLTSSIATNMLGIAGTIGIAFVFVILGVKFIGQQRYKRFFGIFLMTILIFTGLSVLK
DAPTO_512     PIMNLTSSIATNMLGIAGTIGIAFVFVILGVKFIGQQRYKRFFGIFLMTILIFTGLSVLK
S613          PIMNLTSSIATNMLGIAGTIGIAFVFVILGVKFIGQQRYKRFFGIFLMTILIFTGLSVLK

DAPTO_516     ------------------------------------------------------------
TX1467        ------------------------------------------------------------
TX0104        ----------------------------------------PQHKAPNAIEKAQEGLRQAH
TX0027        DANTSNSLFDMMFSVDKEVETAFVNINPVLGDVSVPMTEKGKDKNGNEVEQKLSADQRAK
TX0630        DANTSNSLFDMMFSVDKEVETAFVNINPVLGDVSVPMTEKGKDKNGNEVEQKLSADQRAK
TX1341        DANTSNSLFDMMFSVDKEVETAFVNINPVLGDVSVPMTEKGKDKNGNEVEQKLSADQRAK
TX0017        DANTSNSLFDMMFSVDKEVETAFVNINPVLGDVSVPMTEKGKDKNGNEVEQKLSADQRAK
TX0645        DANTSNSLFDMMFSVDKEVETAFVNINPVLGDVSVPMTEKGKDKNGNEVEQKLSADQRAK
TX0635        DANTSNSLFDMMFSVDKEVETAFVNINPVLGDVSVPMTEKGKDKNGNEVEQKLSADQRAK
TX0860        DANTSNSLFDMMFSVDKEVETAFVNINPVLGDVSVPMTEKGKDKNGNEVEQKLSADQRAK
TX0855        DANTSNSLFDMMFSVDKEVETAFVNINPVLGDVSVPMTEKGKDKNGNEVEQKLSADQRAK
TX4248        DANTSNSLFDMMFSVDKEVETAFVNINPVLGDVSVPMTEKGKDKNGNEVEQKLSADQRAK
TX2134        DANTSNSLFDMMFSVDKEVETAFVNINPVLGDVSVPMTEKGKDKNGNEVEQKLSADQRAK
V583          DANTSNSLFDMMFSVDKEVETAFVNINPVLGDVSVPMTEKGKDKNGNEVEQKLSADQRAK
TX2137        DANTSNSLFDMMFSVDKEVETAFVNINPVLGDVSVPMTEKGKDKNGNEVEQKLSADQRAK
HH22          DANTSNSLFDMMFSVDKEVETAFVNINPVLGDVSVPMTEKGKDKNGNEVEQKLSADQRAK
R712          DANTSNSLFDMMFSVDKEVETAFVNINPVLGDVSVPMTEKGKDKNGNEVEQKLSADQRAK
DAPTO_512     DANTSNSLFDMMFSVDKEVETAFVNINPVLGDVSVPMTEKGKDKNGNEVEQKLSADQRAK
S613          DANTSNSLFDMMFSVDKEVETAFVNINPVLGDVSVPMTEKGKDKNGNEVEQKLSADQRAK

DAPTO_516     ------------------------------------------------------------
TX1467        ---------------------------------RKHGSIKIKNMIRINLLLDNDMNSEEN
TX0104        EKGR-----------------MQEQKSEEQLKRQQQESARQKEYEKDRLKKQESLKK-IN
TX0027        SAGNLIASRVFYTNVYEPYLLMNYGTSDVNKIRKKTVKYKDKEYDRINLLLDNDMNSEEN
TX0630        SAGNLIASRVFYTNVYEPYLLMNYGTSDVNKIRKKTVKYKDKEYDRINLLLDNDMNSEEN
TX1341        SAGNLIASRVFYTNVYEPYLLMNYGTSDVNKIRKKTVKYKDKEYDRINLLLDNDMNSEEN
TX0017        SAGNLIASRVFYTNVYEPYLLMNYGTSDVNKIRKKTVKYKDKEYDRINLLLDNDMNSEEN
TX0645        SAGNLIASRVFYTNVYEPYLLMNYGTSDVNKIRKKTVKYKDKEYDRINLLLDNDMNSEEN
TX0635        SAGNLIASRVFYTNVYEPYLLMNYGTSDVNKIRKKTVKYKDKEYDRINLLLDNDMNSEEN
TX0860        SAGNLIASRVFYTNVYEPYLLMNYGTSDVNKIRKKTVKYKDKEYDRINLLLDNDMNSEEN
TX0855        SAGNLIASRVFYTNVYEPYLLMNYGTSDVNKIRKKTVKYKDKEYDRINLLLDNDMNSEEN
TX4248        SAGNLIASRVFYTNVYEPYLLMNYGTSDVNKIRKKTVKYKDKEYDRINLLLDNDMNSEEN
TX2134        SAGNLIASRVFYTNVYEPYLLMNYGTSDVNKIRKKTVKYKDKEYDRINLLLDNDMNSEEN
V583          SAGNLIASRVFYTNVYEPYLLMNYGTSDVNKIRKKTVKYKDKEYDRINLLLDNDMNSEEN
TX2137        SAGNLIASRVFYTNVYEPYLLMNYGTSDVNKIRKKTVKYKDKEYDRINLLLDNDMNSEEN
HH22          SAGNLIASRVFYTNVYEPYLLMNYGTSDVNKIRKKTVKYKDKEYDRINLLLDNDMNSEEN
R712          SAGNLIASRVFYTNVYEPYLLMNYGTSDVNKIRKKTVKYKDKEYDRINLLLDNDMNSEEN
DAPTO_512     SAGNLIASRVFYTNVYEPYLLMNYGTSDVNKIRKKTVKYKDKEYDRINLLLDNDMNSEEN
S613          SAGNLIASRVFYTNVYEPYLLMNYGTSDVNKIRKKTVKYKDKEYDRINLLLDNDMNSEEN

DAPTO_516     ------------------------------------------------------------
TX1467        NKLMEEVVNYESKDLKNRSIMYYNNWTNTFYGLFYLVVNLIQTVVYFLLSFLRLIIAVIQ
TX0104        DQSDESTNLTALRNQNRRTGQRSKKRETGHQTLMKRQGEKVLQPTNKPKDMXNKWLLVV-
TX0027        DKLMEEVVNYESKDLKNRSIMYYNNWTNTFYGLFYLVVNFIQTVVYFLLSFLRLIIAVIQ
TX0630        DKLMEEVVNYESKDLKNRSIMYYNNWTNTFYGLFYLVVNFIQTVVYFLLSFLRLIIAVIQ
TX1341        DKLMEEVVNYESKDLKNRSIMYYNNWTNTFYGLFYLVVNFIQTVVYFLLSFLRLIIAVIQ
TX0017        DKLMEEVVNYESKDLKNRSIMYYNNWTNTFYGLFYLVVNFIQTVVYFLLSFLRLIIAVIQ
TX0645        DKLMEEVVNYESKDLKNRSIMYYNNWTNTFYGLFYLVVNFIQTVVYFLLSFLRLIIAVIQ
TX0635        NKLMEEVVNYESKDLKNRSIMYYNNWTNTFYGLFYLVVNLIQTVVYFLLSFLRLIIAVIQ
TX0860        NKLMEEVVNYESKDLKNRSIMYYNNWTNTFYGLFYLVVNFIQTVVYFLLSFLRLIIAVIQ
TX0855        NKLMEEVVNYESKDLKNRSIMYYNNWTNTFYGLFYLVVNFIQTVVYFLLSFLRLIIAVI-
TX4248        NKLMEEVVNYESKDLKNRSIMYYNNWTNTFYGLFYLVVNLIQTVVYFLLSFLRLIIAVIQ
```

```
TX2134          DKLMEEVVNYESKDLKNRSIMYYNNWTNTFYGLFYLVVNFIQTVVYFLLSFLRLIIAVIQ
V583            NKLMEEVVNYESKDLKNRSIMYYNNWTNTFYGLFYLVVNFIQTVVYFLLSFLRLIIAVIQ
TX2137          DKLMEEVVNYESKDLKNRSIMYYNNWTNTFYGLFYLVVNFIQTVVYFLLSFLRLIIAVIQ
HH22            DKLMEEVVNYESKDLKNRSIMYYNNWTNTFYGLFYLVVNFIQTVVYFLLSFLRLIIAVIQ
R712            DKLMEEVVNYESKDLKNRSIMYYNNWTNTFYGLFYLVVNFIQTVVYFLLSFLRLIIAVIQ
DAPTO_512       DKLMEEVVNYESKDLKNRSIMYYNNWTNTFYGLFYLVVNFIQTVVYFLLSFLRLIIAVIQ
S613            DKLMEEVVNYESKDLKNRSIMYYNNWTNTFYGLFYLVVNFIQTVVYFLLSFLRLIIAVIQ

DAPTO_516       -------------------------MFTTFGMTIFMKGMVGFATIFFASFLSLGFQLSNQ
TX1467          LFLLPLLPLLLFAGLFLTETNVFANYFKTFGMTIFMKGMVGFATIFFASFLSLGFQLSNQ
TX0104          -----------------QDEQDNQRLVKISNQKIFMKGMVGFATIFFASFLSLGFQLSNQ
TX0027          LFLLPLLPLLLFAGLFLTETNVFANYFKTFGMTIFMKGMVGFATIFFASFLSLGFQLSNQ
TX0630          LFLLPLLPLLLFAGLFLTETNVFANYFKTFGMTIFMKGMVGFATIFFASFLSLGFQLSNQ
TX1341          LFLLPLLPLLLFAGLFLTETNVFANYFKTFGMTIFMKGMVGFATIFFASFLSLGFQLSNQ
TX0017          LFLLPLLPLLLFAGLFLTETNVFANYFKTFGMTIFMKGMVGFATIFFASFLSLGFQLSNQ
TX0645          LFLLPLLPLLLFAGLFLTETNVFANYFKTFGMTIFMKGMVGFATIFFASFLSLGFQLSNQ
TX0635          LFLLPLLPLLLFAGLFLTETNVFANYFKTFGMTIFMKGMVGFATIFFASFLSLGFQLSNQ
TX0860          LFLLPLLPLLLFAGLFLTETNVFANYFKTFGMTIFMKGMVGFATIFFASFLSLGFQLSNQ
TX0855          ------------AGLFLTETNVFANYFKTFGMTIFMKGMVGFATIFFASFLSLGFQLSNQ
TX4248          LFLLPLLPLLLFAGLFLTETNVFANYFKTFGMTIFMKGMVGFATIFFASFLSLGFQLSNQ
TX2134          LFLLPLLPLLLFAGLFLTETNVFANYFKTFGMTIFMKGMVGFATIFFASFLSLGFQLSNQ
V583            LFLLPLLPLLLFAGLFLTETNVFANYFKTFGMTIFMKGMVGFATIFFASFLSLGFQLSNQ
TX2137          LFLLPLLPLLLFAGLFLTETNVFANYFKTFGMTIFMKGMVGFATIFFASFLSLGFQLSNQ
HH22            LFLLPLLPLLLFAGLFLTETNVFANYFKTFGMTIFMKGMVGFATIFFASFLSLGFQLSNQ
R712            LFLLPLLPLLLFAGLFLTETNVFANYFKTFGMTIFMKG----------------------
DAPTO_512       LFLLPLLPLLLFAGLFLTETNVFANYFKTFGMTIFMKGM---------------------
S613            LFLLPLLPLLLFAGLFLTETNVFANYFKTFGMTIFMKG----------------------
                                          ..    .*****

DAPTO_516       TENVWQKILTILIYLLTPLGLYVFRKFFANLVTGRVSLSDGVGFIGNPFGTEANMRRAAK
TX1467          TENVWQKILTILIYLLTPLGLYVFRKFFANLVTGRVSLSDGVGFIGNPFGTEANMRRAAK
TX0104          TENVWQKILTILIYLLTPLGLYVFRKFFANLVTGRVSLSDGVGFIGNPFGTEANMRRAAK
TX0027          TENVWQKILTILIYLLTPLGLYVFRKFFANLVTGRVSLSDGVGFIGNPFGTEANMRRAAK
TX0630          TENVWQKILTILIYLLTPLGLYVFRKFFANLVTGRVSLSDGVGFIGNPFGTEANMRRAAK
TX1341          TENVWQKILTILIYLLTPLGLYVFRKFFANLVTGRVSLSDGVGFIGNPFGTEANMRRASK
TX0017          TENVWQKILTILIYLLTPLGLYVFRKFFANLVTGRVSLSDGVGFIGNPFGTEANMRRAAK
TX0645          TENVWQKILTILIYLLTPLGLYVFRKFFANLVTGRVSLSDGVGFIGNPFGTEANMRRAAR
TX0635          TENVWQKILTILIYLLTPLGLYVFRKFFANLVTGRVSLSDGVGFIGNPFGTEANMRRAAK
TX0860          TENVWQKILTILIYLLTPLGLYVFRKFFANLVTGRVSLSDGVGFIGNPFGTEANMRRAAK
TX0855          TENVWQKILTILIYLLTPLGLYVFRKFFANLVTGRVSLSDGVGFIGNPFGTEANMRRAAK
TX4248          TENVWQKILTILIYLLTPLGLYVFRKFFANLVTGRVSLSDGVGFIGNPFGTEANMRRAAK
TX2134          TENVWQKILTILIYLLTPLGLYVFRKFFANLVTGRVSLSDGVGFIGNPFGTEANMRRAAK
V583            TENVWQKILTILIYLLTPLGLYVFRKFFANLVTGRVSLSDGVGFIGNPFGTEANMRRAAK
TX2137          TENVWQKILTILIYLLTPLGLYVFRKFFANLVTGRVSLSDGVGFIGNPFGTEANMRRAAK
HH22            TENVWQKILTILIYLLTPLGLYVFRKFFANLVTGVFHFLMVSGL-----------SEILL
R712            ------------------------------------------------------------
DAPTO_512       ------------------------------------------------------------
S613            ------------------------------------------------------------

DAPTO_516       EQKQENKERRKQAQEERKAAIKKRQEEARKHGKAETGLKQRPLNKEQEKRSALRRELKPR
TX1467          EQKQENKERRKQAQEERKAAI---------------------------------------
TX0104          EQKQENKERRKQAQEERKAAIKKRQEEARKHGKAETGLKQRPLNKEQEKRSALRRELKPR
TX0027          EQKQENKERRKQAQEERKAAIKKRQEEARKHGKAETGLKQRPLNKEQEKRSALRRELKPR
TX0630          EQKQENKERRKQAQEERKAAIKKRQEEARKHGKAETGLKQRPLNKEQEKRSALRRELKPR
TX1341          EQKQENKERRKQAQEERKAAIKKRQEEARKHGKAETGLKQRPLNKEQEKRSALRRELKPR
TX0017          EQKQENKERRKQAQEERKAAIKKRQEEARKHGKAETGLKQRPLNKEQEKRSALRRELKPR
TX0645          EQKQENKERRKQAQEERKAAIKKRQEEARKHGKAETGLKQRPLNKEQEKRSALRRELKPR
TX0635          EQKQENKERRKQAQEERKAAIKKRQEEARKHGKAETGLKQRPLNKEQEKRSALRRELKPR
TX0860          EQKQENKERRKQAQEERKAAIKKRQEEARKHGKAETGLKQRPLNKEQEKRSALRRELKPR
TX0855          EQKQENKERRKQAQEERKAAIKKRQEEARKHGKAETGLKQRPLNKEQEKRSALRRELKPR
TX4248          EQKQENKERRKQAQEERKAAIKKRQEEARKHGKAETGLKQRPLNKEQEKRSALRRELKPR
```

```
TX2134         EQKQENKERRKQAQEERKAAIKKRQEEARKHGKAETGLKQRPLNKEQEKRSALRRELKPR
V583           EQKQENKERRKQAQEERKAAIKKRQEEARKHGKAETGLKQRPLNKEQEKRSALRRELKPR
TX2137         EQKQENKERRKQAQEERKAAIKKRQEEARKHGKAETGLKQRPLNKEQEKRSALRRELKPR
HH22           AQKLICVERQKNKNKRIRSAGNR----------HKKNVKQLLKNVKKKQGNMVKQKLA--
R712           ------------------------------------------------------------
DAPTO_512      ------------------------------------------------------------
S613           ------------------------------------------------------------

DAPTO_516      PQHKAPNAIEKAQEGLRQAHEKGRMQEQKSEEQLKRQQQESARQKEYEKDRLKKQESLKK
TX1467         ------------------------------------------------------------
TX0104         PQHKAPNAIEKAQEGLRQAHEKGRMQEQKSEEQLKR------------------------
TX0027         PQHKAPNAIEKAQEGLRQAHEKGRMQEQKSEEQLKRKQHESARQKEYEKDRLKKQESLKK
TX0630         PQHKAPNAIEKAQEGLRQAHEKGRMQEQKSEEQLKRQQHESARQKEYEKDRLKKQESLKK
TX1341         PQHKAPNAIEKAQEGLRQAHEKGRMQEQKSEEQLKRQQHESARQKEYEKDRLKKQESLKK
TX0017         PQHKAPNAIEKAQEGLRQVHEKGRMQEQKSEEQLKRQQHESARQKEYEKDRLKKQESLKK
TX0645         PQHKAPNAIEKAQEGLRQAHEKGRMQEQKSEEQLKRQQHESARQKEYEKDRLKKQESLKK
TX0635         PQHKAPNAIEKAQEGLRQAHEKGRMQEQKSEEQLKRQQHESARQKEYEKDRLKKQESLKK
TX0860         PQHKAPNAIEKAQEGLRQAHEKGRMQEQKSEEQLKRQQHESARQKEYEKDRLKKQESLKK
TX0855         PQHKAPNAIEKAQEGLRQAHEKGRMQEQKSEEQLKRQQHESARQKEYEKDRLKKQESLKK
TX4248         PQHKAPNAIEKAQEGLRQAHEKGRMQEQKSEEQLKRQQQESARQKEYEKDRLKKQKSLKK
TX2134         PQHKAPNAIEKAQEGLRQAHEKGRMQEQKSEEQLKRQQQESARQKEYEKDRLKKQESLKK
V583           PQHKAPNAIEKAQEGLRQAHEKGRMQEQKSEEQLKRQQHESARQKEYEKDRLKKQESLKK
TX2137         PQHKAPNAIEKAQEGLRQAHEKGRMQEQKSEEQLKRQQHESARQKEYEKDRLKKQESLKK
HH22           ------------------------------------------------------------
R712           ------------------------------------------------------------
DAPTO_512      ------------------------------------------------------------
S613           ------------------------------------------------------------

DAPTO_516      INDQSDESTNLTALRNQNRRTGQRSKKRETGHQTLMKRQGEKVLQPTNKPKDMKKQVVAR
TX1467         -------------------------------------------KQPTNKPKDMKKQVVAR
TX0104         ------------------------------------------------------------
TX0027         INDQSDESTNLTALRNQNRRTGQRSKKRETGHQTLMKRQGEKVLQPTNKPKDMKKQVVAR
TX0630         I-----------------------------------------------------------
TX1341         INDQSDESTNLTALRNQNRRTGQRSKKRETGHQTLMKRQGEKVLQPTNKPKDMKKQVVAR
TX0017         INDQSDESTNLTALRNQNRRTGQRSKKRETGHQTLMKRQGEKVLQPTNKPKDMKKQVVAR
TX0645         INDQSDESTNLTALRNQNRRTGQRSKKRETGHQTLMKRQGEKVLQPTNKPKDMKKQVVAR
TX0635         INDQSDESTNLTALRNQNRRTGQRSKKRETGHQTLMKRQGEKVLQPTNKPKDMKKQVVAR
TX0860         INDQSDESTNLTALRNQNRRTGQRSKKRETGHQTLMKRQGEKVLQPTNKPKDMKKQVVAR
TX0855         INDQSDESTNLTALRNQNRRTGQRSKKRETGHQTLMKRQGEKVLQPTNKPKDMKKQVVAR
TX4248         INDQSDESTNLTALRNQNRRTGQRSKKRETGHQTLMKRQGEKVLQPTNKPKDMKKQVVAR
TX2134         INDQSDESTNLTALRNQNRRTGQRSKKRETGHQTLMKRQGEKVLQPTNKPKDMKKQVVAR
V583           INDQSDESTNLTALRNQNRRTGQRSKKRETGHQTLMKRQGEKVLQPTNKPKDMKKQVVAR
TX2137         INDQSDESTNLTALRNQNRRTGQRSKKRETGHQTLMKRQGEKVLQPTNKPKDMKKQVVAR
HH22           ------------------------------------------------------------
R712           ------------------------------------------------------------
DAPTO_512      ------------------------------------------------------------
S613           ------------------------------------------------------------

DAPTO_516      SPRRTGQPTTRQNIQPKVAAQTVKRSGGTLGSNPAYRRPEVRKKMDQVKQVTQVDVPQTH
TX1467         SPRRTGQPTTRQNIQPKVAAQTVKRSGGTLGSNPAYRRPEVRKKMDQVKQVTQVDVPQTH
TX0104         ------------------------------------------------------------
TX0027         SPRRTGQPTTRQNIQPKVAAQTVKRSGGTLGSNPAYRRPEVRKKMDQVKQVTQVDVPQTH
TX0630         ------------------------------------------------------------
TX1341         SPRRTGQPTTRQNIQPKVAAQMVKRSGGTLGSNPAYRRPEVRKKMDQVKQVTQVDVPQTH
TX0017         SPRRTGQPTIRQNIQTKVAAQMVKRSCGTLVSNPAYRRPEVRKKMDQVKQVTQVDVPQTH
TX0645         SPRRTGQPTTRQNIQPKVAAQTVKRSGGTLGSNPAYRRPEVRKKMDQVKQVTQVDVPQTH
TX0635         SPRRTGQPTTRQNIQPKVAAQMVKRSGGTLGSNPAYRRPEVRKKMDQVKQVTQVDVPQTH
TX0860         SPRRTGQPTTRQNIQPKVAAQMVKRSGGTLGSNPAYRRPEVRKKMDQVKQVTQVDVPQTH
TX0855         SPRRTGQPTTRQNIQPKVAAQMVKRSGGTLGSNPAYRRPEVRKKMDQVKQVTQVDVPQTH
TX4248         SPRRTGQPTTRQNIQPKVAAQTVKRSGGTLGSNPAYRRPEVRKKMDQVKQVTQVDVPQTH
```

```
TX2134          SPRRTGQPTTRQNIQPKVAAQTVKRSGGTLGSNPAYRRPEVRKKMDQVKQVTQVDVPQTH
V583            SPRRTGQPTTRQNIQPKVAAQTVKRSGGTLGSNPAYRRPEVRKKMDQVKQVTQVDVPQTH
TX2137          SPRRTGQPTTRQNIQPKVAAQTVKRSGGTLGSNPAYRRPEVRKKMDQVKQVTQVDVPQTH
HH22            ------------------------------------------------------------
R712            ------------------------------------------------------------
DAPTO_512       ------------------------------------------------------------
S613            ------------------------------------------------------------


DAPTO_516       STPQRVQPKVNEPITRMNRHNTPIIKKEKKTKQPPVVRRERKKPGTQKITNKPKGVRQPR
TX1467          STPQRVQSKVNEPITRMNRHNTPIIKKEKKTKQPPVVRRERKKPGTQKITNKPKGVRQPR
TX0104          ------------------------------------------------------------
TX0027          STPQRVQPKVNEPITRMNRHNTPIIKKEKRTKQSPVVRRERKKPGTQKITNLKE------
TX0630          ------------------------------------------------------------
TX1341          STPQRVQPKVNEPITRMNRHNTPIIKKEKRTKQSPVVRRERKKPGTQKITNKPKGVRQPR
TX0017          STPQRVQPKVNEPITRMNKHNTPIIKKEKRTKQSPVVRRERKKPGTQKITNKPKGVRQHR
TX0645          STPQRVQPKVNEPITRMNRHNTPIIKKEKRTKQSPVVRRERKKPGTQKITNKPKGVRQPR
TX0635          STPQRVQPKVNEPITRMNRHNTPIIKKEKRTKQSPVVRRERKKPGTQKITNKPKGVRQPR
TX0860          STPQRVQPKVNEPITRMNRHNTPIIKKEKRTKQSPVVRRERKKPGTQKITNKPKGVRQPR
TX0855          STPQRVQPKVNEPITRMNRHNTPIIKKEKRTKQSPVVRRERKKPGTQKITNKPKGVRQPR
TX4248          STPQRVQSKVNEPITRMNRHNTPIIKKEKKTKQPPVVRRERKKPGTQKITNKPKGVRQPR
TX2134          STPQRVQSKVNEPITRMNRHNTPIIKKEKKTKQPPVVRRERKKPGTQKITNKPKGVRQPR
V583            STPQRVQSKVNEPITRMNRHNTPIIKKEKKTKQPPVVRRERKKPGTQKITNKPKGVRQPR
TX2137          STPQRVQSKVNEPITRMNRHNTPIIKKEKKTKQPPVVRRERKKPGTQKITNKPKGVRQPR
HH22            ------------------------------------------------------------
R712            ------------------------------------------------------------
DAPTO_512       ------------------------------------------------------------
S613            ------------------------------------------------------------


DAPTO_516       ARK
TX1467          ARK
TX0104          ---
TX0027          ---
TX0630          ---
TX1341          ARK
TX0017          ARK
TX0645          ARK
TX0635          ARK
TX0860          ARK
TX0855          ARK
TX4248          ARK
TX2134          ARK
V583            ARK
TX2137          ARK
HH22            ---
R712            ---
DAPTO_512       ---
S613            ---
```

2) <u>Lipoprotein (EF501) of *E.faecalis* V583:</u>

```
TX0017          MKLLKWRWQWNKDHKKGEVSMKKVLPFIALVGLLLLSGCGTDMKKILTSDGGKWEGTGFG
TX0860          MKLLKWRWQWNKEHKKGEVSMKKVLPFIALVGLLLLSGCGTDMKKILTSDGGKWEGTGFG
TX1467          MKLPKWRWKWNKDHKKGEVSMKKVLPFIALVGLLLLSGCGTDMKKILTADGGKWEGTGFG
TX0645          MKLLKWRWQWNKDHKKGEVSMKKVLPFIALVGLLLLSGCGSDMKKILTADGGKWEGTGFG
TX2137          -------------------------------------------KKILTADGGKWEGTGFG
TX0635          -----------------------------LVGLLLLSGCGSDMKKILTADGGKWEGTGFG
HH22            MKLLKWRWQWNKDHKKGEVSMKKVLPFIALVGLLLLSGCGSDMKKILTADGGKWEGTGFG
TX4248          MKLLKWRWQWNKDHKKGEVSMKKVLPFIALVGLLLLSGCGSDMKKILTADGGKWEGTGFG
TX1341          MKLLKWRWQWNKDHKKGEVSMKKVLPFIALVGLLLLSGCGTDMKKILTADGGKWEVDEGS
```

```
TX0027          --------------------MKKVLPFIALVGLLLLSGCGTDMKKILTADGGKWEVDEGS
TX0630          ---------------------------------------------ILTADGGKWEVDEGS
TX0104          MKLLKWRWQWNKDHKKGEVSMKKVLPFIALVGLLLLSGCGTDMKKILTADGGKWELENKS
DAPTO_512       MKLLKWRWQWNKDHKKGEVSMKKVLPFIALVGLLLLSGCGTDMKKILTADGGKWELENKS
DAPTO_516       MKLLKWRWQWNKDHKKGEVSMKKVLPFIALVGLLLLSGCGTDMKKILTADGGKWELENKS
R712            MKLLKWRWQWNKDHKKGEVSMKKVLPFIALVGLLLLSGCGTDMKKILTADGGKWELENKS
S613            MKLLKWRWQWNKDHKKGEVSMKKVLPFIALVGLLLLSGCGTDMKKILTADGGKWELENKS
TX2134          MKLLKWRWQWNKDHKKGEVSMKKVLPFIALVGLLLLSGCGTDMKKILTADGGKWKVEET-
V583            ------------------MSMKKVLPFIALVGLLLLSGCGTDMKKILTADGGKWKVEET-
TX0855          ---------------------------------------GTDMKKILTADGGKWKVEET-
                                                             ***:*****:

TX0017          VSTQFTFYKDGEVSLLESGSSYSGTYSYNDKEEKLTIDISNIGKNIFSDVKEKNGKITAK
TX0860          VSTQFTFYKDGEVSLLESGSSYSGTYSYNDKEEKLTIDISNIGKNIFSDVKEKNGKITAK
TX1467          VSTQFTFYKDGEVSLLESGSSYSGTYSYNDKEEKLTIDISNIGKNIFSDVKEKNGKITAK
TX0645          VSTQFTFYKDGEVSLLESGSSYSGTYSYNDKEEKLTIDISNIGKNIFSDVKEKNGKITAK
TX2137          VSTQFTFYKDGEVSLLESGSSYSGTYSYNDKEEKLTIDISNIGKNIFSDVKEKNGKITAK
TX0635          VSTQFTFYKDGEVSLLESGSSYSGTYSYNDKEEKLTIDISNIGKNIFSDVKEKNGKITAK
HH22            VSTQFTFYKDGEVSLLESGSSYSGTYSYNDKEEKLTIDISNIGKNIFSDVKEKNGKITAN
TX4248          VSTQFTFYKDGEVSLLESGSSYSGTYSYNDKEEKLTIDISNIGKNIFSDVKEKNGKITAN
TX1341          SKSTYTFFDDGKFSVYDSKDNAAGKYAYDEKNKKITFDVSGRGTFIMEKVEYKDGKINGE
TX0027          SKSTYTFFDDGKFSVYDSKDNAAGKYSYDEKNKKITFDVSGRGTFIMEKVEYKDGKINGE
TX0630          SKSTYTFFDDGKFSVYDSKDNAAGKYSYDEKNKKITFDVSGRGTFIMEKVEYKDGKINGE
TX0104          PTTTYTFFDDETFSRYNSKISDSGTYSYDENNKKLTLDIKNKEQLIMENVEYKDGKLKGE
DAPTO_512       PTTTYTFFDDETFSRYNSKISDSGTYSYDENNKKLTLDIKNKEQLIMENVEYKDGKLKGE
DAPTO_516       PTTTYTFFDDETFSRYNSKISDSGTYSYDENNKKLTLDIKNKEQLIMENVEYKDGKLKGE
R712            PTTTYTFFDDETFSRYNSKISDSGTYSYDENNKKLTLDIKNKEQLIMENVEYKDGKLKGE
S613            PTTTYTFFDDETFSRYNSKISDSGTYSYDENNKKLTLDIKNKEQLIMENVEYKDGKLKGE
TX2134          -RATYTFFDDGKFSANDSEDSVSGTYTYDEKNKKITFDITSRNSFIMEKVEYKDNKITGE
V583            -RATYTFFDDGKFSANDSEDSVSGTYTYDEKNKKITFDITSRNSFIMEKVEYKDNKITGE
TX0855          -RATYTFFDDGKFSANDSEDSVSGTYTYDEKNKKITFDITSRNSFIMEKVEYKDNKITGE
                  : :**:.*  .*  :*  . :*.*:*:::::*:*:*:.     *:..*: *: *:..:

TX0017          YGKNKISLEKVKEK
TX0860          YGKNKISLEKVKEK
TX1467          YGKNTISLEKVKEK
TX0645          YGKNTISLEKVKEK
TX2137          YGKNTISLEKVKEK
TX0635          YGKNTISLEKVKEK
HH22            YGKNTISLEKVKEK
TX4248          YGKNTISLEKVKEK
TX1341          INDRETVFVKAK--
TX0027          INDRETVFVKEK--
TX0630          INDRETVFVKEK--
TX0104          IGGVEGTLIKK---
DAPTO_512       IGGVEGTLIKK---
DAPTO_516       IGGVEGTLIKK---
R712            IGGVEGTLIKK---
S613            IGGVEGTLIKK---
TX2134          IGEKQRTLIKQKTE
V583            IGEKQRTLIKQKTE
TX0855          IGEKQRTLIKQKTE
```

3) Hypothetical protein (EF500) of *E.faecalis* V583:

```
TX1467          ------------------------------------------------------------
TX0027          MKKTGIKKKGVDKVPLKSKEPRLDFPIEAITNNFLFTTSNDVWVGYKLAHQVFPLNNLDF
TX0635          ----MIAKKGVDKVPLKSKEPRLDFPIEAITNNFLFTTSNDVWVGYKLAHQVFPLNNLDF
TX0860          -------------MPLKSKEPRLDFPIEAITDNFLFTTSNDVWVGYKLAHQVFPLNNLDF
TX1341          MKKIGIKKKGVDKVPLKSKEPRLDFPIEAITDNFLFTTSNDVWVGYKLEHQVFPLNNLDF
TX2134          MKKIGIKKKGVDKVPLKSKEPRLDFPIEAITDNFLFTTSNDVWVGYKLAHQVFPLNNLDF
```

```
TX0630          ---------------------RLDFPIEAITDNFLFTTSNDVWVGYKLAHQVFPLNNLDF
TX0017          -------------MPLKSKEPRLDFPIEAITDNFLFTTSNDVWVGYKLAHQVFPLNNLDF
TX2137          ---------------------------------------DVWVGYKLAHQVFPLNNLDF
V583            -------------MPLKSKEPRLDFPIEAITNNFLFTTSNDVWVGYKLAHQVFPLNNLDF
HH22            ----MIAKKGVDKVPLKSKEPRLDFPIEAITNNFLFTTSNDVWVGYKLAHQVFPLNNLDF
TX4248          ----MIAKKGVDKVPLKSKEPRLDFPIEAITNNFLFTTSNDVWVGYKLAHQVFPLNNLDF
TX0104          ----MIAKKGVDKVPLKSKEPRLDFPIEAITNNFLFTTSNDVWVGYKLAHQVFPLNNLDF
DAPTO_512       ----MIAKKGVDKVPLKSKEPRLDFPIEAITNNFLFTTSNDVWVGYKLAHQVFPLNNLDF
DAPTO_516       ----MIAKKGVDKVPLKSKEPRLDFPIEAITNNFLFTTSNDVWVGYKLAHQVFPLNNLDF
R712            ----MIAKKGVDKVPLKSKEPRLDFPIEAITNNFLFTTSNDVWVGYKLAHQVFPLNNLDF
S613            ----MIAKKGVDKVPLKSKEPRLDFPIEAITNNFLFTTSNDVWVGYKLAHQVFPLNNLDF
TX0645          ----MIAKKGVDKVPLKSKEPRLDFPIEAITNNFLFTTSNDVWVGYKLAHQVFPLNNLDF
TX0855          ----MIAKKGVDKVPLKSKEPRLDFPIEAITNNFLFTTSNDVWVGYKLAHQVFPLNNLDF

TX1467          ------------------------------------------------------------
TX0027          FKEYIEDGKGVFEQDMFDYHFMNIPEYFDLDEQIEATIDKLVKGSFADLGEIYFRQAGDI
TX0635          FKEYIEDGKGVFEQDTFDYHFMNIPEYFDLDEQIEATIDNLVKGSFADLGETYFRQAGDI
TX0860          FKEYIEDGKGVFEQDTFDYHFMNIPEYFDLDEQIEATIDNLVKGSFADLGETYFRQAGDI
TX1341          FKEYIEDGKGVFEQDTFDYHFMNIPEYFDLDEQIEATIDNLVKGSFADLGETYFRQAGDI
TX2134          FKEYIEDGKGVFEQDTFDYHFMNIPEYFDLDEQIEATIDNLVKGSFADLGETYFRQAGDI
TX0630          FKEYIEDGKGVFEQDTFDYHFMNIPEYFDLDEQIEATIDNLVKGSFADLGETYFRQAGDI
TX0017          FKEYIEDGKGVFEQDTFDYHFMNIPEYFDLDEQIEATIDNLVKGSFADLGETYFRQAGDI
TX2137          FKEYIEDGKGVFEQDTFEYHFMNIPEYFDLDEQIEATIDNLVKGSFADLGETYFRQAGDI
V583            FKEYIEDGKGVFEQDTFDYHFMNIPEYFDLDEQIEATIDNLVKGSFADLGETYFRQAGDI
HH22            FKEYIEDGKGVFEQDTFDYHFMNIPEYFDLDEQIEATIDNLVKGSFADLGETYFRQAGDI
TX4248          FKEYIEDGKGVFEQDTFDYHFMNIPEYFDLDEQIEATIDNLVKGSFADLGETYFRQAGDI
TX0104          FKEYIEDGKGVFEQDTFDYHFMNIPEYFDLDEQIEATIDNLVKGSFADLGETYFRQAGDI
DAPTO_512       FKEYIEDGKGVFEQDTFDYHFMNIPEYFDLDEQIEATIDNLVKGSFADLGETYFRQAGDI
DAPTO_516       FKEYIEDGKGVFEQDTFDYHFMNIPEYFDLDEQIEATIDNLVKGSFADLGETYFRQAGDI
R712            FKEYIEDGKGVFEQDTFDYHFMNIPEYFDLDEQIEATIDNLVKGSFADLGETYFRQAGDI
S613            FKEYIEDGKGVFEQDTFDYHFMNIPEYFDLDEQIEATIDNLVKGSFADLGETYFRQAGDI
TX0645          FKEYIEDGKGVFEQDTFDYHFMNIPEYFDLDEQIEATIDNLVKGSFADLGETYFRQAGDI
TX0855          FKEYIEDGKGVFEQDTFDYHFMNIPEYFDLDEQIEATIDNLVKGSFADLGETYFRQAGDI

TX1467          ---------------------------MEYMELFKEVGRKAINALTGVHVPVSQLYRAYQ
TX0027          LKDEVQMNKYSTYLFIRFTAPIQVANPMEYMELFKEVGRKAINALTGVHVPVSQLYRAYQ
TX0635          LKDEVQMNKYSTYLFIRFTAPIQVANPMEYIELFKEVGRKAINALTGVHVPVSQLYRAYQ
TX0860          LKDEVQMNKYSTYLFIRFTAPIQVANPMEYMELFKEVGRKAINALTGVHVPASQLYRAYQ
TX1341          LKDEVQMNKYSTYLFIRFTAPIQVANPMEYMELFKEVGRKAINALTGVHVPVSQLYRAYQ
TX2134          LKDEVQMNKYSTYLFIRFTAPIQVANPMEYMELFKEVGRKAINALTGVHVPVSQLYRAYQ
TX0630          LKDEVQMNKYSTYLFIRFTAPIQVANPMEYIELFKEVGRKAINTLTGVHVPVSQLYRAYQ
TX0017          LKDEVQMNKYSTYLFIRFTAPIQVANPMEYIELFKEVGRKAINALTGVHVPVSQLYRAYQ
TX2137          LKDEVQMNKYSTYLFIRFTAPIQVANPMEYIELFKEVGRKAINALTGVHVPVSQLYRAYQ
V583            LKDEVQMNKYSTYLFIRFTAPIQVANPMEYIELFKEVGRKAINALTGVHVPVSQLYRAYQ
HH22            LKDEVQMNKYSTYLFIRFTAPIQVANPMEYIELFKEVGRKAINALTGVHVPVSQLYRAYQ
TX4248          LKDEVQMNKYSTYLFIRFTAPIQVANPMEYIELFKEVGRKAINALTGVHVPVSQLYRAYQ
TX0104          LKDEVQMNKYSTYLFIRFTAPIQVANPMEYIELFKEVGRKAINALTGVHVPVSQLYRAYQ
DAPTO_512       LKDEVQMNKYSTYLFIRFTAPIQVANPMEYIELFKEVGRKAINALTGVHVPVSQLYRAYQ
DAPTO_516       LKDEVQMNKYSTYLFIRFTAPIQVANPMEYIELFKEVGRKAINALTGVHVPVSQLYRAYQ
R712            LKDEVQMNKYSTYLFIRFTAPIQVANPMEYIELFKEVGRKAINALTGVHVPVSQLYRAYQ
S613            LKDEVQMNKYSTYLFIRFTAPIQVANPMEYIELFKEVGRKAINALTGVHVPVSQLYRAYQ
TX0645          LKDEVQMNKYSTYLFIRFTAPIQVANPMEYIELFKEVGRKAINALTGVHVPVSQLYRAYQ
TX0855          LKDEVQMNKYSTYLFIRFTAPIQVANPMEYIELFKEVGRKAINALTGVHVPVSQLYRAYQ
                                           ***:************:*******.********

TX1467          GLENKIYKDLSNFKNVERLDPRTIGRLFYYFFHRANTRLPERTLLPEEMTEGIIENDTGY
TX0027          GLENKIYKDLSNFKNVERLDPRTIGRLFYYFFHRANTRLPERTLLPEEMTEGIIENNTGY
TX0635          GLENKIYKDLSNFKNVERMDPRTIGRLFYYFFHRANTRLPERTLLPEEMTEGIIENNTGY
TX0860          GLENKIYKDLSNFKNVERMDPRTIGRLFYYFFHRANTRLPERTLLPEEMTEGIIENNTGY
TX1341          GLENKIYKDLSNFKNVERMDPRTIGRLFYYFFHRANTRLPERTLLPEEMTEGIIENNTGY
TX2134          GLENKIYKDLSNFKNVERMDPRTIGRLFYYFFHRANTRLPERTLLPEEMTEGIIENNTGY
```

```
TX0630         GLENKIYKDLSNFKNVERMDPRTIGRLFYYFFHRANTRLPERTLLLEEMTEGIIENNTGY
TX0017         GLENKIYKDLSNFKNVERMDPRTIGRLFYYFFHRVNTRLPERTLLPEEMTEGIIENNTGY
TX2137         GLENKIYKDLSNFKNVERMDPRTIGRLFYYFFHRANTRLPERTLLPEEMTEGIIENNTGY
V583           GLENKIYKDLSNFKNVERMDPRTIGRLFYYFFHRANTRLPERTLLPEEMTEGIIENNTGY
HH22           GLENKIYKDLSNFKNVERMDPRTIGRLFYYFFHRANTRLPERTLLPEEMTEGIIENNTGY
TX4248         GLENKIYKDLSNFKNVERMDPRTIGRLFYYFFHRANTRLPERTLLPEEMTEGIIENNTGY
TX0104         GLENKIYKDLSNFKNVERMDPRTIGRLFYYFFHRANTRLPERTLLPEEMTEGIIENNTGY
DAPTO_512      GLENKIYKDLSNFKNVERMDPRTIGRLFYYFFHRANTRLPERTLLPEEMTEGIIENNTGY
DAPTO_516      GLENKIYKDLSNFKNVERMDPRTIGRLFYYFFHRANTRLPERTLLPEEMTEGIIENNTGY
R712           GLENKIYKDLSNFKNVERMDPRTIGRLFYYFFHRANTRLPERTLLPEEMTEGIIENNTGY
S613           GLENKIYKDLSNFKNVERMDPRTIGRLFYYFFHRANTRLPERTLLPEEMTEGIIENNTGY
TX0645         GLENKIYKDLSNFKNVERMDPRTIGRLFYYFFHRANTRLPERTLLPEEMTEGIIENNTGY
TX0855         GLENKIYKDLSNFKNVERMDPRTIGRLFYYFFHRANTRLPERTLLPEEMTEGIIENNTGY
               ******************:***************.********** **********:***

TX1467         MTIEQLDKTHYLAFLPLISLPTSMFGSAIIQNLQDSLSTTIETHVKVTFRHPDKDKRDVH
TX0027         MTIEQLDKTHYLAFLPLISLPTSMFGSAIIQNLQDSLSTTIETHVKVTFRHPDKDKRDVH
TX0635         MTIEQLDKTHYLAFLPLISLPTSMFGSAIIQNLQDSLSTTIETHVKVTFRHPDKDKRDVH
TX0860         MTIEQLNKTHYLAFLPLISLPTSMFGSAIIQNLQDSLSTTIETHVKVTFRHPDKDKRDVH
TX1341         MTIEQLDKTHYLAFLPLISLPTSMFGSAIIQNLQDSLSTTIETHVKVTFRHPDKDKRDVH
TX2134         MTIEQLDKTHYLAFLPLISLPTSMFGSAIIQNLQDSLSTTIETHVKVTFRHPDKDKRDVH
TX0630         MTIEQLDKTHYLAFLPLISLPTSMFGSAIIQNLQDSLSTTIETHVKVTFRHPDKDKRDVH
TX0017         MTIEQLDKTHYLAFLPLISLPTSMFGSAIIQNLQDSLSTTIETHVKVTFRHPDKDKRDVH
TX2137         MTIEQLDKTHYLAFLPLISLPTSMFGSAIIQNLQDSLSTTIETHVKVTFRHPDKDKRDVH
V583           MTIEQLDKTHYLAFLPLISLPTSMFGSAIIQNLQDSLSTTIETHVKVTFRHPDKDKRDVH
HH22           MTIEQLDKTHYLAFLPLISLPTSMFGSAIIQNLQDSLSTTIETHVKVTFRHPDKDKRDVH
TX4248         MTIEQLDKTHYLAFLPLISLPTSMFGSAIIQNLQDSLSTTIETHVKVTFRHPDKDKRDVH
TX0104         MTIEQLDKTHYLAFLPLISLPTSMFGSAIIQNLQDSLSTTIETHVKVTFRHPDKDKRDVH
DAPTO_512      MTIEQLDKTHYLAFLPLISLPTSMFGSAIIQNLQDSLSTTIETHVKVTFRHPDKDKRDVH
DAPTO_516      MTIEQLDKTHYLAFLPLISLPTSMFGSAIIQNLQDSLSTTIETHVKVTFRHPDKDKRDVH
R712           MTIEQLDKTHYLAFLPLISLPTSMFGSAIIQNLQDSLSTTIETHVKVTFRHPDKDKRDVH
S613           MTIEQLDKTHYLAFLPLISLPTSMFGSAIIQNLQDSLSTTIETHVKVTFRHPDKDKRDVH
TX0645         MTIEQLDKTHYLAFLPLISLPTSMFGSAIIQNLQDSLSTTIETHVKVTFRHPDKDKRDVH
TX0855         MTIEQLDKTHYLAFLPLISLPTSMFGSAIIQNLQDSLSTTIETHVKVTFRHPDKDKRDVH
               ******:*****************************************************

TX1467         KRRKRIYEQDKEQTQVDGILDEDEVILFGEERLHELSKKIRSKERRLCRMTTTFVLSADS
TX0027         KRRKRIYEQDKEQTQVDGILDEDEVILFGEERLHELSKKIRSKERRLCRMTTTFVLSADS
TX0635         KRRKRIYEQDKEQTQVDGILDEDEVILFGEERLHELSKKIRSKERRLCRMTTTFVLSADS
TX0860         KRRKRIYEQDKEQTQVDGILDEDEVILFGEERLHELSKKIRRKERRLCRMTTTFVLSADS
TX1341         KRRKRIYEQDKEQTQVDGILDEDEVILFGEERLHELSKKIRSKERRLCRMTTTFVLSADS
TX2134         KRRKRIYEQDKEQTQVDGILDEDEVILFGEERLHELSKKIRSKERRLCRMTTTFVLSADS
TX0630         KRRKRIYEQDKEQTQVDGILDEDEVILFGEERLHELSKKIRSKERRLCRMTTTFVLSADS
TX0017         KRRKRIYEQDKEQTQVDGILDEDEVILFGEERLHELSKKIRSKERRLCRMTTTFVLSADS
TX2137         KRRKRIYEQDKEQTQVDGILDEDEVILFGEERLHELSKKIRSKERRLCRMTTTFVLSADS
V583           KRRKRIYEQDKEQTQVDGILDEDEVILFGEERLHELSKKIRSKERRLCRMTTTFVLSADS
HH22           KRRKRIYEQDKEQTQVDGILDEDEVILFGEERLHELSKKIRSKERRLCRMTTTFVLSADS
TX4248         KRRKRIYEQDKEQTQVDGILDEDEVILFGEERLHELSKKIRSKERRLCRMTTTFVLSADS
TX0104         KRRKRIYEQDKEQTQVDGILDEDEVILFGEERLHELSKKIRSKERRLCRMTTTFVLSADS
DAPTO_512      KRRKRIYEQDKEQTQVDGILDEDEVILFGEERLHELSKKIRSKERRLCRMTTTFVLSADS
DAPTO_516      KRRKRIYEQDKEQTQVDGILDEDEVILFGEERLHELSKKIRSKERRLCRMTTTFVLSADS
R712           KRRKRIYEQDKEQTQVDGILDEDEVILFGEERLHELSKKIRSKERRLCRMTTTFVLSADS
S613           KRRKRIYEQDKEQTQVDGILDEDEVILFGEERLHELSKKIRSKERRLCRMTTTFVLSADS
TX0645         KRRKRIYEQDKEQTQVDGILDEDEVILFGEERLHELSKKIRSKERRLCRMTTTFVLSADS
TX0855         KRRKRIYEQDKEQTQVDGILDEDEVILFGEERLHELSKKIRSKERRLCRMTTTFVLSADS
               ***************************************** ******************

TX1467         KQALEEKIKELEFVLDGTDYKLYRPLVDQLTLFNQCLIGAKSQFKSYEHVVSTGYVADLG
TX0027         KQALEEKIKELEFVLDGTDYKLYRPLVDQLTLFNQCLIGAKSQFKSYEHVVSTGYVADLG
TX0635         KQALEEKIKELEFVLDGTDYKLYRPLVDQLTLFNQCLIGAKNQFKSYEHVVSTGYVADLG
TX0860         KQALEEKIKELEFVLDGTDYKLYRPLVDQLTLFNQCLIGAKNQFKSYEHVVSTGYVADLG
TX1341         KQVLEEKIKELEFVLDGTDYKLYRPLVDQLTLFNQCLIGAKSQFKSYEHVVSTGYVADLG
TX2134         KQALEEKIKELEFVLDGTDYKLYRPLVDQLTLFNQCLIGAKSQFKSYEHVVSTGYVADLG
```

```
TX0630         KQALEEKIKELEFVLDGTDYKLYRPLVDQLTLFNQCLIGAKSQFKSYEHVVSTGYVADLG
TX0017         KQALEEKIKELEFVLDGTDYKLYRPLVDQLTLFNQCLIGAKSQFKSYEHVVSTGYVADLG
TX2137         KQALEEKIKELEFVLDGTDYKLYRPLVDQLTLFNQCLIGAKSQFKSYEHVVSTGYVADLG
V583           KQALEEKIKELEFVLDGTDYKLYRPLVDQLTLFNQCLIGAKSQFKSYEHVVSTGYVADLG
HH22           KQALEEKIKELEFVLDGTDYKLYRPLVDQLTLFNQCLIGAKSQFKSYEHVVSTGYVADLG
TX4248         KQALEEKIKELEFVLDGTDYKLYRPLVDQLTLFNQCLIGAKSQFKSYEHVVSTGYVADLG
TX0104         KQALEEKIKELEFVLDGTDYKLYRPLVDQLTLFNQCLIGAKSQFKSYEHVVSTGYVADLG
DAPTO_512      KQALEEKIKELEFVLDGTDYKLYRPLVDQLTLFNQCLIGAKSQFKSYEHVVSTGYVADLG
DAPTO_516      KQALEEKIKELEFVLDGTDYKLYRPLVDQLTLFNQCLIGAKSQFKSYEHVVSTGYVADLG
R712           KQALEEKIKELEFVLDGTDYKLYRPLVDQLTLFNQCLIGAKSQFKSYEHVVSTGYVADLG
S613           KQALEEKIKELEFVLDGTDYKLYRPLVDQLTLFNQCLIGAKSQFKSYEHVVSTGYVADLG
TX0645         KQALEEKIKELEFVLDGTDYKLYRPLVDQLTLFNQCLIGAKNQFKSYEHVVSTGYVADLG
TX0855         KQALEEKIKELEFVLDGTDYKLYRPLVDQLTLFNQCLIGAKSQFKSYEHVVSTGYVADLG
               **.**************************************.******************

TX1467         MDLEKEIGNRYGMPLGRVITAKKFKSVQQALSLSSKIVWFFPNLTKKAIEGAQYTNGNTL
TX0027         MDLEKEIGNRYGMPLGRVITAKKFKSVQQALSLSSKIVWFFPNLTKKAIEGAQYTNGNTL
TX0635         MDLEKEIGNRYGMPLGRVITAKKFKSVQQALSLSSKIVWFFPNLTKKAIEGAQYTNGNTL
TX0860         MDLEKEIGNRYGMPLGRVITAKKFKSVQQALSLSSKIVWFFPNLTKKAIEGAQYTNGNTL
TX1341         MDLEKEIGNRYGMPLGRVITAKKFKSVQQALSLSSKIVWFFPNLTKKAIEGAQYTNGNTL
TX2134         MDLEKEIGNRYGMPLGRVITAKKFKSVQQALSLSSKIVWFFPNLTKKAIEGAQYTNGNTL
TX0630         MDLEKEIGNRYGMPLGRVITAKKFKSVQQALSLSSKIVWFFPNLTKKAIEGAQYTNGNTL
TX0017         MDLEKEIGNRYGMPLGRVITAKKFKSVQQALSLSSKIVWFFPNLTKKAIEGAQYTNGNTL
TX2137         MDLEKEIGNRYGMPLGRVITAKKFKSVQQALSLSSKIVWFFPNLTKKAIEGAQYTNGNTL
V583           MDLEKEIGNRYGMPLGRVITAKKFKSVQQALSLSSKIVWFFPNLTKKAIEGAQYTNGNTL
HH22           MDLEKEIGNRYGMPLGRVITAKKFKSVQQALSLSSKIVWFFPNLTKKAIEGAQYTNGNTL
TX4248         MDLEKEIGNRYGMPLGRVITAKKFKSVQQALSLSSKIVWFFPNLTKKAIEGAQYTNGNTL
TX0104         MDLEKEIGNRYGMPLGRVITAKKFKSVQQALSLSSKIVWFFPNLTKKAIEGAQYTNGNTL
DAPTO_512      MDLEKEIGNRYGMPLGRVITAKKFKSVQQALSLSSKIVWFFPNLTKKAIEGAQYTNGNTL
DAPTO_516      MDLEKEIGNRYGMPLGRVITAKKFKSVQQALSLSSKIVWFFPNLTKKAIEGAQYTNGNTL
R712           MDLEKEIGNRYGMPLGRVITAKKFKSVQQALSLSSKIVWFFPNLTKKAIEGAQYTNGNTL
S613           MDLEKEIGNRYGMPLGRVITAKKFKSVQQALSLSSKIVWFFPNLTKKAIEGAQYTNGNTL
TX0645         MDLEKEIGNRYGMPLGRVITAKKFKSVQQALSLSSKIVWFFPNLTKKAIEGAQYTNGNTL
TX0855         MDLEKEIGNRYGMPLGRVITAKKFKSVQQALSLSSKIVWFFPNLTKKAIEGAQYTNGNTL
               ************************************************************

TX1467         IIGPPGQGKSVLVKYIFLWLTFLGQKILYIDPKNETVLFFKRALDKFGHIPEFKALYQRI
TX0027         IIGPPGQGKSVLVKYIFLWLTFLGQKILYIDPKNETVLFFRRALDKFGHIPEFKALYQRI
TX0635         IIGPPGQGKSVLVKYIFLWLTFLGQKILYIDPKNETVLFFRRALDKFGHIPEFKALYQRI
TX0860         IIGPPGQGKSVLVKYIFLWLTFLGQKILYIDPKNETVLFFRRALDKFGHIPEFKALYQRI
TX1341         IIGPPGQGKSVLVKYIFLWLTFLGQKILYIDPKNETVLFFRRALDKFGHIPEFKALYQRI
TX2134         IIGPPGQGKSVLVKYIFLWLTFLGQKILYIDPKNETVLFFRRALDKFGHIPEFKALYQRI
TX0630         IIGPPGQGKSVLVKYIFLWLTFLGQKILYIDPKNETVLFFKRALDKFGHIPEFKALYQRI
TX0017         IIGPPGQGKSVLVKYIFLWLTFLGQKILYIDPKNETVLFFKRALDKFGHIPEFKALYQRI
TX2137         IIGPPGQGKSVLVKYIFLWLTFLGQKILYIDPKNETVLFFKRALDKFGHIPEFKALYQRI
V583           IIGPPGQGKSVLVKYIFLWLTFLGQKILYIDPKNETVLFFKRALDKFGHIPEFKALYQRI
HH22           IIGPPGQGKSVLVKYIFLWLTFLGQKILYIDPKNETVLFFKRALDKFGHIPEFKALYQRI
TX4248         IIGPPGQGKSVLVKYIFLWLTFLGQKILYIDPKNETVLFFKRALDKFGHIPEFKALYQRI
TX0104         IIGPPGQGKSVLVKYIFLWLTFLGQKILYIDPKNETVLFFKRALDKFGHIPEFKALYQRI
DAPTO_512      IIGPPGQGKSVLVKYIFLWLTFLGQKILYIDPKNETVLFFKRALDKFGHIPEFKALYQRI
DAPTO_516      IIGPPGQGKSVLVKYIFLWLTFLGQKILYIDPKNETVLFFKRALDKFGHIPEFKALYQRI
R712           IIGPPGQGKSVLVKYIFLWLTFLGQKILYIDPKNETVLFFKRALDKFGHIPEFKALYQRI
S613           IIGPPGQGKSVLVKYIFLWLTFLGQKILYIDPKNETVLFFKRALDKFGHIPEFKALYQRI
TX0645         IIGPPGQGKSVLVKYIFLWLTFLGQKILYIDPKNETVLFFKRALDKFGHIPEFKALYQRI
TX0855         IIGPPGQGKSVLVKYIFLWLTFLGQKILYIDPKNETVLFFKRALDKFGHIPEFKALYQRI
               ****************************************:*******************

TX1467         NFVSLSSEERYRGMLDPLLFLPREEAIQTARNVLENFGEVTTDSHTASDKKTLILDSVNA
TX0027         NFVSLSSEERYRGMLDPLLFLPREEAIQTARNVLENFGEVTTDSHTASDKKTLILDSVNA
TX0635         NFVSLSSEERYRGMLDPLLFLPREEAIQTARNVLENFGEVTTDSHTASDKKTLILDSVNA
TX0860         NFVSLSSEERYRGMLDPLLFLPREEAIQTARNVLENFGEVTTDSHTASDKKTLILDSVNA
TX1341         NFVSLSSEERYRGMLDPLLFLPREEAIQTARNVLENFGEVTTDSHTASDKKTLILDSVNA
TX2134         NFVSLSSEERYRGMLDPLLFLPREEAIQTARNVLENFGEVTTDSHTASDKKTLILDSVNA
```

```
TX0630        NFVSLSSEERYRGMLDPLLFLPREEAIQTARNVLENFGEVTTDSHTASDKKTLILDSVNA
TX0017        NFVSLSSEEKYRGMLDPLLFLPREEAIQTARNVLENFGEVTTDSHTASDKKTLILDSVNA
TX2137        NFVSLSSEERYRGMLDPLLFLPREEAIQTARNVLENFGEVTTDSHTASDKKTLILDSVNA
V583          NFVSLSSEERYRGMLDPLLFLPREEAIQTARNVLENFGEVTTDSHTASDKKTLILDSVNA
HH22          NFVSLSSEEKYRGMLDPLLFLPREEAIQTARNVLENFGEVTTDSHTASDKKTLILDSVNA
TX4248        NFVSLSSEEKYRGMLDPLLFLPREEAIQTARNVLENFGEVTTDSHTASDKKTLILDSVNA
TX0104        NFVSLSSEERYRGMLDPLLFLPREEAIQTARNVLENFGEVTTDSHTASDKKTLILDSVNA
DAPTO_512     NFVSLSSEERYRGMLDPLLFLPREEAIQTARNVLENFGEVTTDSHTASDKKTLILDSVNA
DAPTO_516     NFVSLSSEERYRGMLDPLLFLPREEAIQTARNVLENFGEVTTDSHTASDKKTLILDSVNA
R712          NFVSLSSEERYRGMLDPLLFLPREEAIQTARNVLENFGEVTTDSHTASDKKTLILDSVNA
S613          NFVSLSSEERYRGMLDPLLFLPREEAIQTARNVLENFGEVTTDSHTASDKKTLILDSVNA
TX0645        NFVSLSSEERYRGMLDPLLFLPREEAIQTARNVLENFGEVTTDSHTASDKKTLILDSVNA
TX0855        NFVSLSSEERYRGMLDPLLFLPREEAIQTARNVLENFGEVTTDSHTASDKKTLILDSVNA
              *********:**************************************************

TX1467        VMKGKGKKHLTKVIEVIREKDPKLANLISGHNVGLGKILLGNDYSEPIRFENQINVLGTQ
TX0027        VMKGKGKKHLTKVIEVIREKDPKLANLISGHNVGLGKILLGNDYSEPIRFENQINVLGTQ
TX0635        VMKGKGKKHLTKVIEVIREKDPKLANLISGHNVGLGKILLGNDYSEPIRFENQINVLGTQ
TX0860        VMKGKGKKHLTKVIEVIREKDPKLANLISGHNVGLGKILLGNDYSEPIRFENQINVLGTQ
TX1341        VMKGKGKKHLTKVIEVIREKDPKLANLISGHNVGLGKILLGNDYSEPIRFENQINVLGTQ
TX2134        VMKGKGKKHLTKVIEVIREKDPKLANLISGHNVGLGKILLGNDYSEPIRFENQINVLGTQ
TX0630        VMKGKGKKHLTKV-----------------------------------------------
TX0017        VMKGKGKKHLTKVIEVIREKDPKLANLISGHNVGLGKILLGNDYSEPIRFENQINVLGTQ
TX2137        VMKGKGKKHLTKVIEVIREKDPKLANLISGHNVGLGKILLGNDYSEPIRFENQINVLGTQ
V583          VMKGKGKKHLTKVIEVIREKDPKLANLISGHNVGLGKILLGNDYSEPIRFENQINVLGTQ
HH22          VMKGKGKKHLTKVIEVIREKDPKLANLISGHNVGLGKILLGNDYSEPIRFENQINVLGTQ
TX4248        VMKGKGKKHLTKVIEVIREKDPKLANLISGHNVGLGKILLGNDYSEPIRFENQINVLGTQ
TX0104        VMKGKGKKHLTKVIEVIREKDPKLANLISGHNVGLGKILLGNDYSEPIRFENQINVLGTQ
DAPTO_512     VMKGKGKKHLTKVIEVIREKDPKLANLISGHNVGLGKILLGNDYSEPIRFENQINVLGTQ
DAPTO_516     VMKGKGKKHLTKVIEVIREKDPKLANLISGHNVGLGKILLGNDYSEPIRFENQINVLGTQ
R712          VMKGKGKKHLTKVIEVIREKDPKLANLISGHNVGLGKILLGNDYSEPIRFENQINVLGTQ
S613          VMKGKGKKHLTKVIEVIREKDPKLANLISGHNVGLGKILLGNDYSEPIRFENQINVLGTQ
TX0645        VMKGKGKKHLTKVIEVIREKDPKLANLISGHNVGLGKILLGNDYSEPIRFENQINVLGTQ
TX0855        VMKGKGKKHLTKVIEVIREKDPKLANLISGHNVGLGKILLGNDYSEPIRFENQINVLGTQ
              *************

TX1467        GLKIPTQAEIDSGRLNNEQIAGMSIMEVIMKMTTIFSTDKTEDAAIIFDEAKGFEDTAQG
TX0027        GLKIPTQAEIDSGRLNNEQIAGMSIMEVIMKMTTIFSTDKTEDAAIIFDEAKGFEDTAQG
TX0635        GLKIPTQAEIDSGRLNNEQIAGMSIMEVIMKMTTIFSTDKTEDAAIIFDEAKGFEDTAQG
TX0860        GLKIPTQAEIDSGRLNNEQIAGMSIMEVIMKMTTIFSTDKTEDAAIIFDEAKGFEDTAQG
TX1341        GLKIPTQAEIDSGRLNNEQIAGMSIMEVIMKMTTIFSTDKTEDAAIIFDEAKGFEDTAQG
TX2134        GLKIPTQAEIDSGRLNNEQIAGMSIMEVIMKMTTIFSTDKTEDAAIIFDEAKGFEDTAQG
TX0630        ------------------------------------------------------------
TX0017        GLKIPTQAEIDSGRLNNEQIAGMSIMEVIMKMTTIFSTDKTEDAAIIFDEAKGFEDTAQG
TX2137        GLKIPTQAEIDSGRLNNEQIAGMSIMEVIMKMTTIFSTDKTEDAAIIFDEAKGFEDTAQG
V583          GLKIPTQAEIDSGRLNNEQIAGMSIMEVIMKMTTIFSTDKTEDAAIIFDEAKGFEDTAQG
HH22          GLKIPTQAEIDSGRLNNEQIAGMSIMEVIMKMTTIFSTDKTEDAAIIFDEAKGFEDTAQG
TX4248        GLKIPTQAEIDSGRLNNEQIAGMSIMEVIMKMTTIFSTDKTEDAAIIFDEAKGFEDTAQG
TX0104        GLKIPTQAEIDSGRLNNEQIAGMSIMEVIMKMTTIFSTDKTEDAAIIFDEAKGFEDTAQG
DAPTO_512     GLKIPTQAEIDSGRLNNEQIAGMSIMEVIMKMTTIFSTDKTEDAAIIFDEAKGFEDTAQG
DAPTO_516     GLKIPTQAEIDSGRLNNEQIAGMSIMEVIMKMTTIFSTDKTEDAAIIFDEAKGFEDTAQG
R712          GLKIPTQAEIDSGRLNNEQIAGMSIMEVIMKMTTIFSTDKTEDAAIIFDEAKGFEDTAQG
S613          GLKIPTQAEIDSGRLNNEQIAGMSIMEVIMKMTTIFSTDKTEDAAIIFDEAKGFEDTAQG
TX0645        GLKIPTQAEIDSGRLNNEQIAGMSIMEVIMKMTTIFSTDKTEDAAIIFDEAKGFEDTAQG
TX0855        GLKIPTQAEIDSGRLNNEQIAGMSIMEVIMKMTTIFSTDKTEDAAIIFDEAKGFEDTAQG


TX1467        RFLIEGSLRQGRANLTDIYLVTQAFMDYDKEDKKELLSYKFAFRPNQKEAQKKVLEFFWY
TX0027        RFLIEGSLRQGRANLTDIYLVTQAFMDYDKEDKKELLSYKFAFRPNQKEAQKKVLEFFGM
TX0635        RFLIEGSLRQGRANLTDIYLVTQAFMDYDKEDKKELLSYKFAFRPNQKEAQKKVLEFFGM
TX0860        RFLIEGSLRQGRANMTDIYLVTQAFMDYDKEDKKELLSYKFAFRPNQKEAQKKVLEFFGM
TX1341        RFLIEGSLRQGRANMTDIYLVTQAFMDYDKEDKKELLSYKFAFRPNQKEAQKKVLEFFGM
TX2134        RFLIEGSLRQGRANMTDIYLVTQAFMDYDKEDKKELLSYKFAFRPNQKEAQKKVLEFFGM
```

```
TX0630         ------------------------------------------------------------
TX0017         RFLIEGSLRQGRANMTDIYLVTQAFMDYDKEDKKELLSYKFAFRPNQKEAQKKVLEFFGM
TX2137         RFLIEGSLRQGRANMTDIYLVTQAFMDYDKEDKKELLSYKFAFRPNQKEAQKKVLEFFGM
V583           RFLIEGSLRQGRANMTDIYLVTQAFMDYDKEDKKELLSYKFAFRPNQKEAQKKVLEFFGM
HH22           RFLIEGSLRQGRANMTDIYLVTQAFMDYDKEDKKELLSYKFAFRPNQKEAQKKVLEFFGM
TX4248         RFLIEGSLRQGRANMTDIYLVTQAFMDYDKEDKKELLSYKFAFRPNQKEAQKKVLEFFGM
TX0104         RFLIEGSLRQGRANMTDIYLVTQAFMDYDKEDKKELLSYKFAFRPNQKEAQKKVLEFFGM
DAPTO_512      RFLIEGSLRQGRANMTDIYLVTQAFMDYDKEDKKELLSYKFAFRPNQKEAQKKVLEFFGM
DAPTO_516      RFLIEGSLRQGRANMTDIYLVTQAFMDYDKEDKKELLSYKFAFRPNQKEAQKKVLEFFGM
R712           RFLIEGSLRQGRANMTDIYLVTQAFMDYDKEDKKELLSYKFAFRPNQKEAQKKVLEFFGM
S613           RFLIEGSLRQGRANMTDIYLVTQAFMDYDKEDKKELLSYKFAFRPNQKEAQKKVLEFFGM
TX0645         RFLIEGSLRQGRANMTDIYLVTQAFMDYDKEDKKELLSYKFAFRPNQKEAQKKVLEFFGM
TX0855         RFLIEGSLRQGRANMTDIYLVTQAFMDYDKEDKKELLSYKFAFRPNQKEAQKKVLEFFGM


TX1467         GYESSKYSVN--------------------------------------------------
TX0027         DTNPANIQLINGLKSGTCLFQDHLGRSQPIAIDVLFDSWLMAISSTNKEDEATKVALEME
TX0635         DTNPANIQLINGLKSGTCLFQDHLGRSQPIAIDVLFDSWLMAISSTNKEDEATKVALEME
TX0860         DTNPANLQLINELKSGTCLFQDHRGRSQPIAIDVLFDSWLMAVSSTNKEDEATQMALAME
TX1341         DTNPANIQLINELKSGTCLFQDHRGRSQPIAIDVLFDSWLMAVSSTNKEDEATQMALAME
TX2134         DTNPANIQLINELKSGTCLFQDHRGRSQPIAIDVLFDSWLMAVSSTNKEDEATQMALAME
TX0630         ------------------------------------------------------------
TX0017         DTNPANIQLINELKSGTCLFQDHRGRSQPIAIDVLFDSWLMAVSSTNKEDEATQMALAME
TX2137         DTNPANLQLINELKSGTCLFQDHRGRSQPIAIDVLFDSWLMAVSSTNKEDEATQMALAME
V583           DTNPANIQLINELKSGTCLFQDHRGRSQPIAIDVLFDSWLMAVSSTNKEDEATQMALAME
HH22           DTNPANIQLINELKSGTCLFQDHRGRSQPIAIDVLFDSWLMAVSSTNKEDEATQMALAME
TX4248         DTNPANIQLINELKSGTCLFQDHRGRSQPIAIDVLFDSWLMAVSSTNKEDEATQMALAME
TX0104         DTNPANLQLINELKSGTCLFQDHRGRSQPIAIDVLFDSWLMAVSSTNKEDEATQMALAME
DAPTO_512      DTNPANLQLINELKSGTCLFQDHRGRSQPIAIDVLFDSWLMAVSSTNKEDEATQMALAME
DAPTO_516      DTNPANLQLINELKSGTCLFQDHRGRSQPIAIDVLFDSWLMAVSSTNKEDEATQMALAME
R712           DTNPANLQLINELKSGTCLFQDHRGRSQPIAIDVLFDSWLMAVSSTNKEDEATQMALAME
S613           DTNPANLQLINELKSGTCLFQDHRGRSQPIAIDVLFDSWLMAVSSTNKEDEATQMALAME
TX0645         DTNPANIQLINELKSGTCLFQDHRGRSQPIAIDVLFDSWLMAVSSTNKEDEATQMALAME
TX0855         DTNPANIQLINELKSGTCLFQDHRGRSQPIAIDVLFDSWLMAVSSTNKEDEATQMALAME


TX1467         ---
TX0027         QGS
TX0635         QGS
TX0860         QGT
TX1341         QGS
TX2134         QGS
TX0630         ---
TX0017         QGS
TX2137         QGS
V583           QGS
HH22           QGS
TX4248         QGS
TX0104         QGS
DAPTO_512      QGS
DAPTO_516      QGS
R712           QGS
S613           QGS
TX0645         QGS
TX0855         QGS
```

4) Hypothetical protein (EF503) of *E.faecalis* V583:

```
DAPTO_512      DGDMRYNPVEHGNDISIRDKLVSLAETESVFYSGAAKALLQVTIQLLDEFKGAKVTLSGD
TX0017         DGDMRYNPVEHGNDISIRDKLVSLAETESVFYSGAAKALLQVTIQLLDEFKGAKVTLSGD
TX0630         DGDMRYNPVEHGNDISIRDKLVSLAETESVFYSGAAKALLQVTIQLLDEFKGAKVTLSGD
```

```
TX0860          DGDMRYNPVEHGNDISIRDKLVSLAETESVFYSGAAKALLQVTIQLLDEFKGAKVTLSGD
TX4248          DGDMRYNPVEHGNDISIRDKLVSLAETESVFYSGAAKALLQVTIQLLDEFKGAKVTLSGD
TX0855          ------------------------------------------------------------
TX2134          DGDMRYNPVEHGNDISIRDKLVSLAETESVFYSGAAKALLQVTIQLLDEFKGAKVTLSGD
TX1341          DGDMRYNPVEHGNDISIRDKLVSLAETESVFYSGAAKALLQVTIQLLDEFKGAKVTLSGD
TX0645          DGDMRYNPVEHGNDISIRDKLVSLAETESVFYSGAAKALLQVTIQLLDEFKGAKVTLSGD
V583            DGDMRYNPVEHGNDISIRDKLVSLAETESVFYSGAAKALLQVTIQLLDEFKGAKVTLSGD
TX0104          DGDMRYNPVEHGNDISIRDKLVSLAETESVFYSGAAKALLQVTIQLLDEFKGAKVTLSGD
TX1467          DGDMRYNPVEHGNDISIRDKLVSLAETESVFYSGAAKALLQVTIQLLDEFKGAKVTLSGD
TX2137          DGDMRYNPVEHGNDISIRDKLVSLAETESVFYSGAAKALLQVTIQLLDEFKGAKVTLSGD
TX0635          DGDMRYNPVEHGNDISIRDKLVSLAETESVFYSGAAKALLQVTIQLLDEFKGAKVTLSGD
DAPTO_516       DGDMRYNPVEHGNDISIRDKLVSLAETESVFYSGAAKALLQVTIQLLDEFKGAKVTLSGD
R712            DGDMRYNPVEHGNDISIRDKLVSLAETESVFYSGAAKALLQVTIQLLDEFKGAKVTLSGD
S613            DGDMRYNPVEHGNDISIRDKLVSLAETESVFYSGAAKALLQVTIQLLDEFKGAKVTLSGD
TX0027          DGDMRYNPVEHGNDVSIRDKLVALAETESVFYSSAAKLLLENTVQLIDLFST--------
HH22            DGDMRYNPVEHGNDVSIRDKLVSLAETESVFYSSAAKLLLENTVQLIDLFST--------


DAPTO_512       TRTTETVERSLPFVQRFLLPRNVLHLFADAILPNNPKLFEIEVEKKIQKPKKKSVKEGSE
TX0017          TRTTETVERSLPFVQRFLLPRNVLHLFADAILPNNPKLFEIEVEKKIQQPKKKSVKEGSE
TX0630          TRTTETVERSLPFVQRFLLPRNVLHLFADAILPNNPKLFEIEVEKKIQKPKKKSVKEGSE
TX0860          TRTTETVERSLPFVQRFLLPRNVLHLFADAILPNNPKLFEIEVEKKIQKPKKKSVKEGSE
TX4248          TRTTETVERSLPFVQRFLLPRNVLHLFADAILPNNPKLFEIEVEKKIQKPKKKSVKEGSE
TX0855          --TTETVERSLPFVQRFLLPRNVLHLFADAILPNNPKLFEIEVEKKIQKPKKKSVKEGSE
TX2134          TRTTETVERSLPFVQRFLLPRNVLHLFADAILPNNPKLFEIEVEKKIQKPKKKSVKEGSE
TX1341          TRTTETVERSLPFVQRFLLPRNVLHLFADAILPNNPKLFEIEVEKKIQKPKKKSVKEGSE
TX0645          TRTTETVERSLPFVQRFLLPRNVLHLFADAILPNNPKLFEIEVEKKIQKPKKKSVKEGSE
V583            TRTTETVERSLPFVQRFLLPRNVLHLFADAILPNNPKLFEIEVEKKIQKPKKKSVKEGSE
TX0104          TRTTETVERSLPFVQRFLLPRNVLHLFADAILPNNPKLFEIEVEKKIQKPKKKSVKEGSE
TX1467          TRTTETVERSLPFVQRFLLPRNVLHLFADAILPNNPKLFEIEVEKKIQKPKKKSVKEGSE
TX2137          TRTTETVERSLPFVQRFLLPRNVLHLFADAILPNNPKLFEIEVEKKIQKPKKKSVKEGSE
TX0635          TRTTETVERSLPFVQRFLLPRNVLHLFADAILPNNPKLFEIEVEKKIQKPKKKSVKEGSE
DAPTO_516       TRTTETVERSLPFVQRFLLPRNVLHLFADAILPNNPKLFEIEVEKKIQKPKKKSVKEGSE
R712            TRTTETVERSLPFVQRFLLPRNVLHLFADAILPNNPKLFEIEVEKKIQKPKKKSVKEGSE
S613            TRTTETVERSLPFVQRFLLPRNVLHLFADAILPNNPKLFEIEVEKKIQKPKKKSVKEGSE
TX0027          ---RDDVNRKLEDIQKYLLPRNVLRLFADKIEEKNPTLYEIEVEVKQAKPKKKSKKNET-
HH22            ---RDDVNRKLEDIQKYLLPRNVLRLFADKIEGKNPTLYEIEVEVKQAKPKKKSKKNET-
                    : *:*.*  :*::*******:**** *  :**.*:***** *  :***** *: :

DAPTO_512       ILPDSDLDKEEKEEDSQIKNSKF----RNISQLGIAQKETETIVLNPETLDLDSYYLLLK
TX0017          ILPDSDLDKEEKEEDSQIKNSKF----RNISQLGIAQKETETIVLNPETLDLDSYYLLLK
TX0630          ILPDSDLDKEEKEEDSQIKNSKF----RNISQLGIAQKETETIVLNPETLDLDSYYLLLK
TX0860          ILPDSDLDKEEKEEDSQIKNSKF----RNISQLGIAQKETETIVLNPETLDLDSYYLLLK
TX4248          ILPDSDLDKEEKEEDSQIKNSKF----RNISQLGIAQKETETIVLNPETLDLDSYYLLLK
TX0855          ILPDSDLDKEEKEEDSQIKNSKF----RNISQLGIAQKETETIVLNPETLDLDSYYLLLK
TX2134          ILPDSDLDKEEKEEDSQIKNSKF----RNISQLGIAQKETETIVLNPETLDLDSYYLLLK
TX1341          ILPDSDLDKEEKEEDSQIKNSKF----RNISQLGIAQKETETIVLNPETLDLDSYYLLLK
TX0645          ILPDSDLDKEEKEEDSQIKNSKF----RNISQLGIAQKETETIVLNPETLDLDSYYLLLK
V583            ILPDSDLDKEEKEEDSQIKNSKF----RNISQLGIAQKETETIVLNPETLDLDSYYLLLK
TX0104          ILPDSDLDKEEKEEDSQIKNSKF----RNISQLGIAQKETETIVLNPETLDLDSYYLLLK
TX1467          ILPDSDLDKEEKEEDSQIKNSKF----RNISQLGIAQKETETIVLNPETLDLDSYYLLLK
TX2137          ILPDSDLDKEEKEEDSQIKNSKF----RNISQLGIAQKETETIVLNPETLDLDSYYLLLK
TX0635          ILPDSDLDKEEKEEDSQIKNSKF----RNISQLGIAQKETETIVLNPETLDLDSYYLLLK
DAPTO_516       ILPDSDLDKEEKEEDSQIKNSKF----RNISQLGIAQKETETIVLNPETLDLDSYYLLLK
R712            ILPDSDLDKEEKEEDSQIKNSKF----RNISQLGIAQKETETIVLNPETLDLDSYYLLLK
S613            ILPDSDLDKEEKEEDSQIKNSKF----RNISQLGIAQKETETIVLNPETLDLDSYYLLLK
TX0027          KVPIIDEEMDEVSEATTPTNDEVTSEDLELEEGDIPPVETEIIVLNPNTLQLDDFYYLLK
HH22            KVPIIDEEMDEVSEATTPTNDEVTSEDLELEEGDIPPVETEIIVLNPNTLQLDDFYYLLK
                 :*  * : :* .* :  .*.:.     ::.:  *   *** *****:**:**.:* ***

DAPTO_512       RNLRYLPTDKETGENIKQKLFERLFIRYEHKDSSFYLYATSEALQTNINMLLDSELGKLF
TX0017          RNLRYLPTDKETGENIKQKLFERLFIRYEHKDSPFYLYATSEALQTNINMLLDSELGKLF
TX0630          RNLRYLPTDKETGENIKQKLFERLFIRYEHKDSPFYLYATSEALQTNINMLLDSELGKLF
```

```
TX0860        RNLRYLPTDKETGENIKQKLFERLFIRYEHKDSPFYLYATSEALQTNINMLLDSELGKLF
TX4248        RNLRYLPTDKETGENIKQKLFERLFIRYEHKDSPFYLYATSEALQTNINMLLDSELGKLF
TX0855        RNLRYLPTDKETGENIKQKLFERLFIRYEHKDSPFYLYATSEALQTNINMLLDSELGKLF
TX2134        RNLRYLPTDKETGENIKQKLFERLFIRYEHKDSPFYLYATSEALQTNINMLLDSELGKLF
TX1341        RNLRYLPTDKETGENIKQKLFERLFIRYEHKDSPFYLYATSEALQTNINMLLDSELGKLF
TX0645        RNLRYLPTDKETGENIKQKLFERLFIRYEHKDSPFYLYATSEALQTNINMLLDSELGKLF
V583          RNLRYLPTDKETGENIKQKLFERLFIRYEHKDSPFYLYATSEALQTNINMLLDSELGKLF
TX0104        RNLRYLPTDKETGENIKQKLFERLFIRYEHKDSPFYLYATSEALQTNINMLLDSELGKLF
TX1467        RNLRYLPTDKETGENIKQKLFERLFIRYEHKDSPFYLYATSEALQTNINMLLDSELGKLF
TX2137        RNLRYLPTDKETGENIKQKLFERLFIRYEHKDSPFYLYATSEALQTNINMLLDSELGKLF
TX0635        RNLRYLPTDKETGENIKQKLFERLFIRYEHKDSPFYLYATSEALQTNINMLLDSELGKLF
DAPTO_516     RNLRYLPTDKETGENIKQKLFERLFIRYEHKDSPFYLYATSEALQTNINMLLDSELGKLF
R712          RNLRYLPTDKETGENIKQKLFERLFIRYEHKDSPFYLYATSEALQTNINMLLDSELGKLF
S613          RNLRYLPTDKETGENIKQKLFERLFIRYEHKDSPFYLYATSEALQTNINMLLDSELGKLF
TX0027        RNLFYLSKKE-------KIMFERLFTRYEHKDSPFYLYATSESLQTNINMLLDSELGHLF
HH22          RNLFYLSKKE-------KIMFERLFTRYEHKDSPFYLYATSESLQTNINMLLDSELGHLF
              *** ** ..:       : :***** ******* ********:**************:**

DAPTO_512     DTKNAKNVLDVQEIVNQRKLVYYVSFNGLIYKEYIRTLAQMLVGDVNYFASEMYRKNVKRE
TX0017        DTKNAKNVLDVQEIVNQRKLVYYVSFNGLIYKEYIRTLAQMLVGDVNYFASEMYRKNVKRE
TX0630        DTKNAKNVLDVQEIVNQRKLVYYVSFNGLIYKEYIRTLAQMLVGDVNYFASEMYRKNVKRE
TX0860        DTKNAKNVLDVQEIVNQRKLVYYVSFNGLIYKEYIRTLAQMLVGDVNYFASEMYRKNVKRE
TX4248        DTKNAKNVLDVQEIVNQRKLVYYVSFNGLIYKEYIRTLAQMLVGDVNYFASEMYRKNVKRE
TX0855        DTKNAKNVLDVQEIVNQRKLVYYVSFNGLIYKEYIRTLAQMLVGDVNYFASEMYRKNVKRE
TX2134        DTKNAKNVLDVQEIVNQRKLVYYVSFNGLIYKEYIRTLAQMLVGDVNYFASEMYRKNVKRE
TX1341        DTKNAKNVLDVQEIVNQRKLVYYVSFNGLIYKEYIRTLAQMLVGDVNYFASEMYRKNVKRE
TX0645        DTKNAKNVLDVQEIVNQRKLVYYVSFNGLIYKEYIRTLAQMLVGDVNYFASEMYRKNVKRE
V583          DTKNAKNVLDVQEIVNQRKLVYYVSFNGLIYKEYIRTLAQMLVGDVNYFASEMYRKNVKRE
TX0104        DTKNAKNVLDVQEIVNQRKLVYYVSFNGLIYKEYIRTLAQMLVGDVNYFASEMYRKNVKRE
TX1467        DTKNAKNVLDVQEIVNQRKLVYYVSFNGLIYKEYIRTLAQMLVGDVNYFASEMYRKNVKRE
TX2137        DTKNAKNVLDVQEIVNQRKLVYYVSFNGLIYKEYIRTLAQMLVGDVNYFASEMYRKNVKRE
TX0635        DTKNAKNVLDVQEIVNQRKLVYYVSFNGLIYKEYIRTLAQMLVGDVNYFASEMYRKNVKRE
DAPTO_516     DTKNAKNVLDVQEIVNQRKLVYYVSFNGLIYKEYIRTLAQMLVGDVNYFASEMYRKNVKRE
R712          DTKNAKNVLDVQEIVNQRKLVYYVSFNGLIYKEYIRTLAQMLVGDVNYFASEMYRKNVKRE
S613          DTKNAKNVLDVQEIVNQRKLVYYVSFNGLIYKEYIRTLAQMLVGDVNYFASEMYRKNVKRE
TX0027        DTTNAKSVLDVQQIVRDRSLVYYVSFNGLIYKEYIRTLAQMLVGDVNYFASEMYRKNVKRE
HH22          DTTNAKSVLDVQQIVRDRSLVYYVSFNGLIYKEYIRTLAQMLVGDVNYFASEMYRKNVKRE
              **.***.*****:**.:*.******************************************

DAPTO_512     VLVIFDEPASYLNETFIDMVNKGRGAGVYGIFTPQTMADIAKLGDKLMEQLVGNVNTLFI
TX0017        VLVIFDEPASYLNETFIDMVNKGRGAGVYGIFTPQTMADIAKLGDKLMEQLVGNVNTLFI
TX0630        VLVIFDEPASYLNETFIDMVNKGRGAGVYGIFTPQTMADIAKLGDKLMEQLVGNVNTLFI
TX0860        VLVIFDEPASYLNETFIDMVNKGRGAGVYGIFTPQTMADIAKLGDKLMEQLVGNVNTLFI
TX4248        VLVIFDEPASYLNETFIDMVNKGRGAGVYGIFTPQTMADIAKLGDKLMEQLVGNVNTLFI
TX0855        VLVIFDEPASYLNETFIDMVNKGRGAGVYGIFTPQTMADIAKLGDKLMEQLVGNVNTLFI
TX2134        VLVIFDEPASYLNETFIDMVNKGRGAGVYGIFTPQTMADIAKLGDKLMEQLVGNVNTLFI
TX1341        VLVIFDEPASYLNETFIDMVNKGRGAGVYGIFTPQTMADIAKLGDKLMEQLVGNVNTLFI
TX0645        VLVIFDEPASYLNETFIDMVNKGRGAGVYGIFTPQTMADIAKLGDKLMEQLVGNVNTLFI
V583          VLVIFDEPASYLNETFIDMVNKGRGAGVYGIFTPQTMADIAKLGDKLMEQLVGNVNTLFI
TX0104        VLVIFDEPASYLNETFIDMVNKGRGAGVYGIFTPQTMADIAKLGDKLMEQLVGNVNTLFI
TX1467        VLVIFDEPASYLNETFIDMVNKGRGAGVYGIFTPQTMADIAKLGDKLMEQLVGNVNTLFI
TX2137        VLVIFDEPASYLNETFIDMVNKGRGAGVYGIFTPQTMADIAKLGDKLMEQLVGNVNTLFI
TX0635        VLVIFDEPASYLNETFIDMVNKGRGAGVYGIFTPQTMADIAKLGDKLMEQLVGNVNTLFI
DAPTO_516     VLVIFDEPASYLNETFIDMVNKGRGAGVYGIFTPQTMADIAKLGDKLMEQLVGNVNTLFI
R712          VLVIFDEPASYLNETFIDMVNKGRGAGVYGIFTPQTMADIAKLGDKLMEQLVGNVNTLFI
S613          VLVIFDEPASYLNETFIDMVNKGRGAGVYGIFTPQTMADIAKLGDKLMEQLVGNVNTLFI
TX0027        VIVIFDEPASYLNEQFIDMVNKGRGAGVHGIFTPQTMADIAKLGDKLMEQLVGNVNTVFI
HH22          VIVIFDEPASYLNEQFIDMVNKGRGAGVHGIFTPQTMADIAKLGDKLMEQLVGNVNTVFI
              *:************ *************:****************************:**

DAPTO_512     GKTNEKGEAEYWSETMGTYQDIDVTSVTEQEDGYSDVGKSDWSGDRGTKRNVDRFKISPN
TX0017        GKTNEKGEAEYWSETMGTYQDIDVTSVTEQEDGYSDVGKSDWSGDRGTKRNVDRFKISPN
TX0630        GKTNEKGEAEYWSETMGTYQDIDVTSVTEQEDGYSDVGKSDWSGDRGTKRNVDRFKISPN
```

```
TX0860         GKTNEKGEAEYWSETMGTYQDIDVTSVTEQEDGYSDVGKSDWSGDRGTKRNVDRFKISPN
TX4248         GKTNEKGEAEYWSETMGTYQDIDVTSVTEQEDGYSDVGKSDWSGDRGTKRNVDRFKISPN
TX0855         GKTNEKGEAEYWSETMGTYQDIDVTSVTEQEDGYSDVGKSDWSGDRGTKRNVDRFKISPN
TX2134         GKTNEKGEAEYWSETMGTYQDIDVTSVTEQEDGYSDVGKSDWSGDRGTKRNVDRFKISPN
TX1341         GKTNEKGEAEYWSETMGTYQDIDVTSVTEQEDGYSDVGKSDWSGDRGTKRNVDRFKISPN
TX0645         GKTNEKGEAEYWSETMGTYQDIDVTSVTEQEDGYSDVGKSDWSGDRGTKRNVDRFKISPN
V583           GKTNEKGEAEYWSETMGTYQDIDVTSVTEQEDGYSDVGKSDWSGDRGTKRNVDRFKISPN
TX0104         GKTNEKGEAEYWSETMGTYQDIDVTSVTEQEDGYSDVGKSDWSGDRGTKRNVDRFKISPN
TX1467         GKTNEKGEAEYWSETMGTYQDIDVTSVTEQEDGYSDVGKSDWSGDRGTKRNVDRFKISPN
TX2137         GKTNEKGEAEYWSETMGTYQDIDVTSVTEQEDGYSDVGKSDWSGDRGTKRNVDRFKISPN
TX0635         GKTNEKGEAEYWSETMGTYQDIDVTSVTEQEDGYSDVGKSDWSGDRGTKRNVDRFKISPN
DAPTO_516      GKTNEKGEAEYWSETMGTYQDIDVTSVTEQEDGYSDVGKSDWSGDRGTKRNVDRFKISPN
R712           GKTNEKGEAEYWSETMGTYQDIDVTSVTEQEDGYSDVGKSDWSGDRGTKRNVDRFKISPN
S613           GKTNEKGEAEYWSETMGTYQDIDVTSVTEQEDGYSDVGKSDWSGDRGTKRNVDRFKISPN
TX0027         GKTNEKGEAEYWSETMGTYQDIDVTSVTEQEDGYSDVGKSDWSGDRGTKRNVDRFKVNPN
HH22           GKTNEKGEAEYWSETMGTYQDIDVTSVTEQEDGYSDVGKSDWSGDRGTKRNVDRFKVNPN
               ********************************************************:.**

DAPTO_512      KIKELRTGEFIIYRTAENVNL--------------------
TX0017         KIKELRTGEFIIYRTAENVNLPPQKVYVRNALNWLKNNRKI
TX0630         KIKELRTGEFIIYRTAENVNLPPQKVYVRNALNWLKNNRKI
TX0860         KIKELRTGEFIIYRTAENVNLPPQKVYVRNALNWLKNNRKI
TX4248         KIKELRTGEFIIYRTAENVNLPPQKVYVRNALNWLKNNSKI
TX0855         KIKELRTGEFIIYRTAENVNLPPQKVYVRNALEWLQKSNSK
TX2134         KIKELRTGEFIIYRTAENVNLPPQKVYVRNALNWLKNNSKI
TX1341         KIKELRTGEFIIYRTAENVNLPPQKVYVRNALNWLKNNSKI
TX0645         KIKELRTGEFIIYRTAENVNLPPQKVYVRNALNWLKNNSKI
V583           KIKELRTGEFIIYRTAENVNLPPQKVYVRNALEWLQKSNSK
TX0104         KIKELRTGEFIIYRTAENVNL--------------------
TX1467         KIKELRTGEFIIYRTAENVNLPPQKVYVRNALEWLQKSNSK
TX2137         KIKELRTGEFIIYRTAENVNL--------------------
TX0635         KIKELRTGEFIIYRTAENVNL--------------------
DAPTO_516      KIKELRTGEFIIYRTAENVNL--------------------
R712           KIKELRTGEFIIYRTAENVNL--------------------
S613           KIKELRTGEFIIYRTAENVNL--------------------
TX0027         VIKSLRTGEFIIYRTAENVNVPPQKVYVRNALEWLRTHNGI
HH22           VIKSLRTGEFIIYRTAENVNVPPQKVYVRNALEWLRTHNGI
                **.****************:
```

Figure S5: Multiple sequence alignment of Pathogen and Putative Pathogen exclusive proteins of *E.faecalis* strains

Sequence : > EF0500
Length : 830 amino acid residues

■ Blast PDB **(3D structure)**
■ PFAM domain**(sequence motif)**
■ SCOP domain**(classified structure)**
■ SEG **(low complexity region)**

DICHOT
■ Known structural domain
■ Novel (cryptic) structural domain
■ Intrinsically disordered region

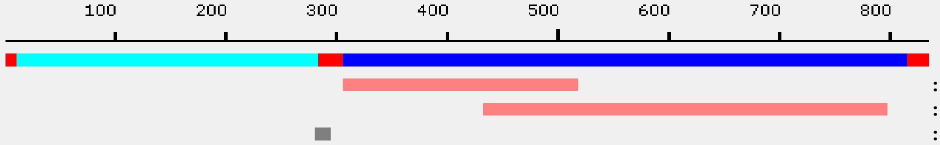

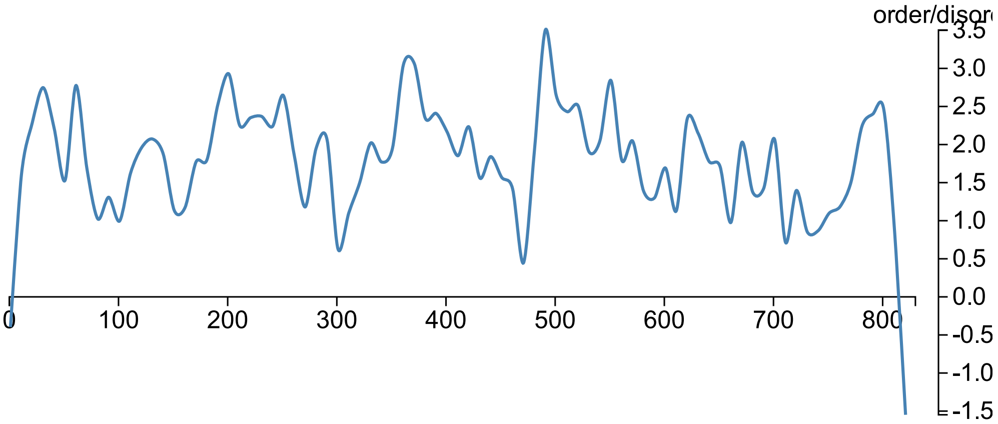

DICHOT
Known structural doamins: 305-812;
Cryptic structural domains: 11-282;
Intrinsically disordered regions: 1-10; 283-304; 813-830;

Details of HMMER-aligned SCOP domain

SCOP ID	c.37.1.19
PDB ID	1gm5A3
	P-loop containing nucleoside triphosphate hydrolases
Expectation value	0.00036
Sequence identity	20.0 %

Details of HMMER-aligned SCOP domain

SCOP ID	c.37.1.11
PDB ID	1e9rA_
	P-loop containing nucleoside triphosphate hydrolases
Expectation value	2.5e-25
Sequence identity	19.7 %

Copyright (C) 2011 TeamIDEAL All Rights Reserved.

Sequence : > EF0502

Length : 781 amino acid residues

■ Blast PDB **(3D structure)**
■ PFAM domain**(sequence motif)**
■ SCOP domain**(classified structure)**
■ SEG **(low complexity region)**

DICHOT
■ Known structural domain
■ Novel (cryptic) structural domain
■ Intrinsically disordered region

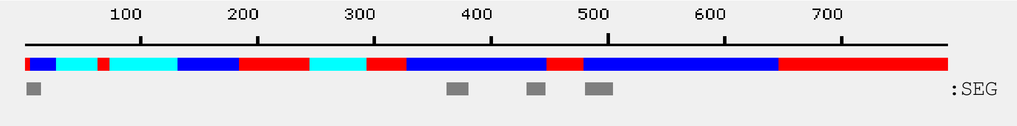

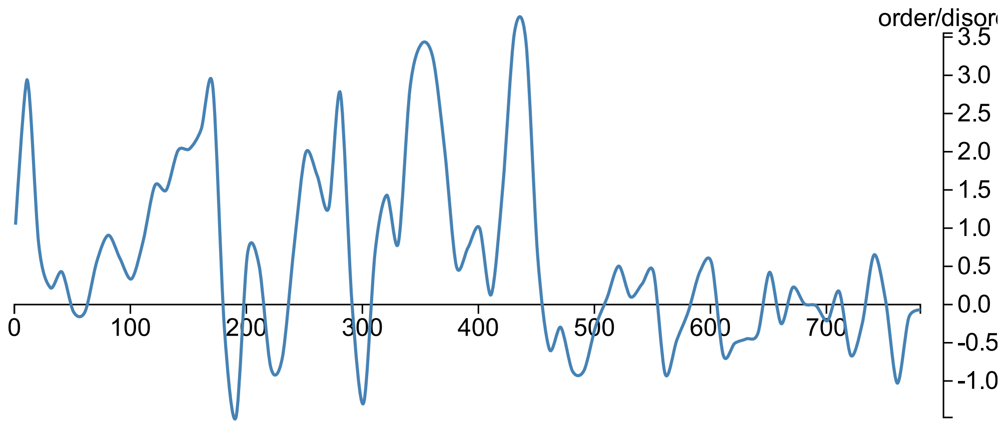

DICHOT

Known structural doamins: 5-27; 131-182; 325-442; 474-639;

Cryptic structural domains: 28-62; 73-130; 243-290;

Intrinsically disordered regions: 1-4; 63-72; 183-242; 291-324; 443-473; 640-781;

Copyright (C) 2011 Team IDEAL. All Rights Reserved.

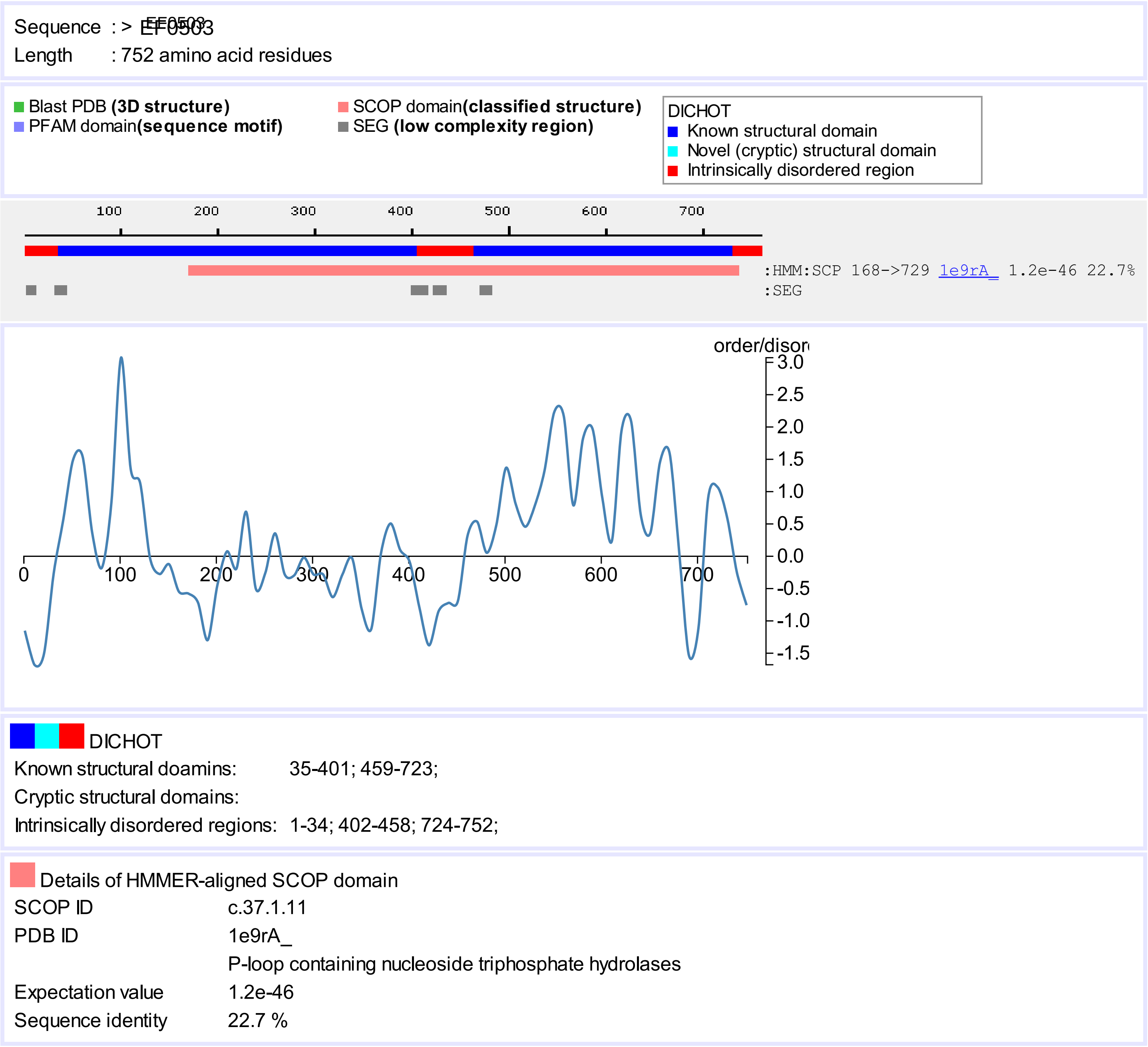

Figure S6 - Identification of Intrinsically Disordered Regions (IDRs) in three pathogen specific proteins. IDRs are identified by sequence comparison with various structural features viz. PDB Blast, PFAM and SCOP domain search and SEG analysis in DICHOT tool of IDEAL (Intrinsically Disordered proteins with Extensive Annotations and Literature) database. Final order-disorder regions are shown in line diagram along the length of protein.

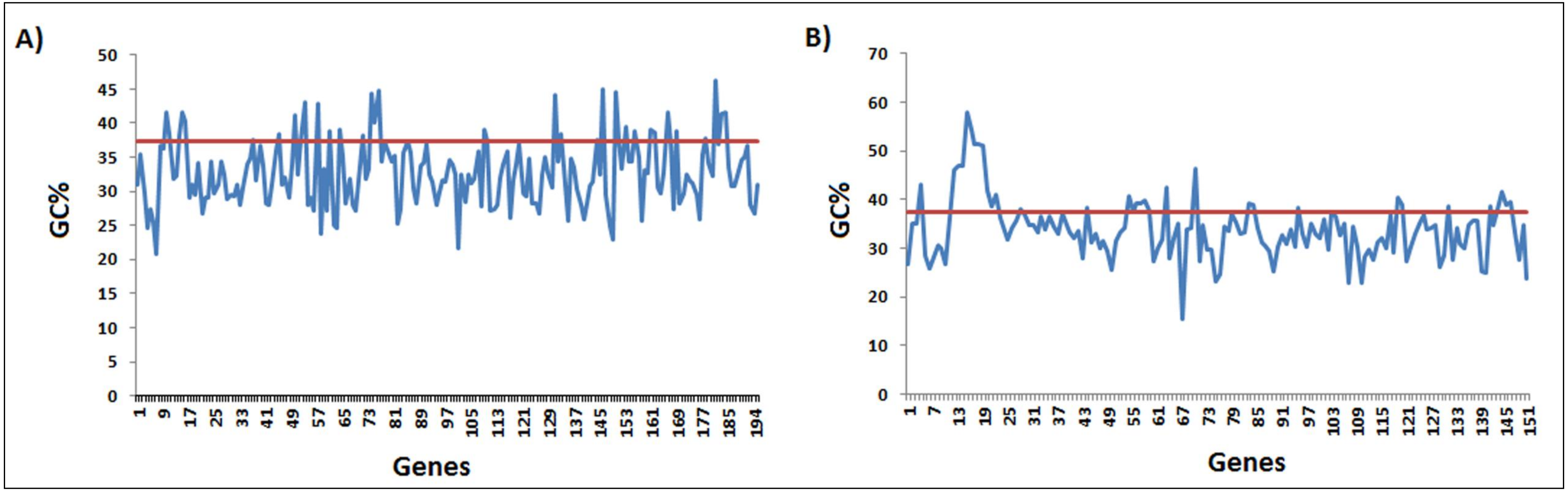

Figure S7 – Average G+C contents of A) Blood (N = 194) and B) GIT (N =150) specific genes. The average G+C content of individual genes are shown in blue lines, whereas the red lines indicate average genomic G+C of the *E.faecalis* strains.

Table S1: Details of the Pathogenic strains specific genes of *E.faecalis*.

Details of the genes present only in pathogenic strains of *Enterococcus faecalis*					
***Enterococcus faecalis* Strain**	**Gene ID**	**Protein_length**	**Locus_tag**	**COG**	**Protein product**
Enterococcus faecalis V583, DAPTO 512, DAPTO 516, R712, S613, TX0630, TX0645, TX0104, TX2137, HH22,TX0635	29374797	901	EF0146	-	Surface exclusion protein
	29375127	152	EF0499	COG0629L	Single-strand binding protein
	29375128	830	EF0500	COG3451U	Hypothetical protein
	29375129	114	EF0501	-	Lipoprotein
	29375130	781	EF0502	-	Hypothetical protein
	29375131	752	EF0503	COG3505U	Hypothetical protein
	29375135	88	EF0507	-	Hypothetical protein
	29375136	166	EF0508	COG0484O	Hypothetical protein
	29375139	194	EF0511	COG1525L	Thermonuclease
	29375141	91	EF0513	COG3041S	Hypothetical protein
	29375569	130	EF0987	-	Lipoprotein
	29376096	47	EF1532	-	Hypothetical protein
	29376367	1866	EF1824	COG1501G	Glycosyl hydrolase family protein
	29376387	81	EF1849	-	Hypothetical protein
	29376494	82	EF1972	-	Hypothetical protein
	29376540	86	EF2025	-	Hypothetical protein
	29377132	227	EF2648	COG0398S	Hypothetical protein
	29377357	84	EF2892	-	Hypothetical protein
	29377401	69	EF2937	-	Hypothetical protein

Table S2: Distribution of Virulence factors in *E.faecalis* plasmids

A) Major virulence factors in Enterococcus which are considered in this study [1]:

Class	Function	Remarks
**Adherence**		
AS	Aggregation substance	Contributes to pathogenicity by enhancing cell adhesion to cultured renal tubular cells and intestinal epithelial cells and may involved in internalization into enterocytes
Ace	Adhesin to collagen of *E. faecalis*	Mediating binding to immobilized collagen type I, collagen type IV, and mouse laminin
Acm	Adherence	Ace and Acm share sequence similarity with a large family of collagen-binding MSCRAMMs of Gram-positive pathogens, of which Cna of Staphylococcus aureus is the prototype
EfaA	Adherence	Might be functioning as an adhesin in endocarditis
Scm	second collagen adhesin	Binds to collagen type V and fibrinogen
**Antiphagocytosis**		
Capsule	Antiphagocytic cells	Contributes to host immune evasion
**Biofilm formation**		
BopD	Biofilm formation	The actual role is unknown, but the association of enhanced biofilm formation in the presence of glucose and the possible involvement of a sugar-binding transcriptional regulator suggest a linkage to increased biofilm production in *E. faecalis* in the presence of specific carbohydrates
Fsr	Biofilm formation , Quorum sensing	The Fsr quorum sensing system is an important regulator with both positive and negative effects, regulating *gelE*, *sprE* and *bopD* expression that are important for biofilm formation, along with genes implicated in several metabolic pathways
**Exoenzyme**		
Gelatinase	Exoenzyme	A member of the matrix metalloproteinase (MMP) family
Hyaluronidase	Exoenzyme, Spreading factor	An important pathogenic bacterial spreading factor, and cleave hyaluronan, which is a constituent of the extracellular matrix of connective tissues
SprE	Exoenzyme; Serine protease	Contributes to pathogenesis in several infection models including *C. elegans*, mouse peritonitis, and a rabbit endophthalmitis model

B) Presence - absence matrix of major virulence factors in *E.faecalis* plasmid: **(+)= Presence (-)= Absence**

Plasmids	AS	Ace	Acm	EfaA	Scm	Capsule	BopD	Fsr	Gelatinase	Hyaluronidase	SprE
EF62pA	-	-	-	-	-	-	-	-	-	-	-
EF62pB	+	-	-	-	-	-	-	-	-	-	-
EF62pC	+	-	-	-	-	-	-	-	-	-	-
EFD32pA	-	-	-	-	-	-	-	-	-	-	-
EFD32pB	-	-	-	-	-	-	-	-	-	-	-
pAM373	+	-	-	-	-	-	-	-	-	-	-
pAMalpha1	-	-	-	-	-	-	-	-	-	-	-
pAMbeta1	-	-	-	-	-	-	-	-	-	-	-
pBEE99	+	-	-	-	-	-	-	-	-	-	-
pCF10	+	-	-	-	-	-	-	-	-	-	-
pCPPF5	-	-	-	-	-	-	-	-	-	-	-
pEF-01	-	-	-	-	-	-	-	-	-	-	-
pEF10244	+	-	-	-	-	-	-	-	-	-	-
pEF1071	-	-	-	-	-	-	-	-	-	-	-
pEFPL_DORA_14	-	-	-	-	-	-	-	-	-	-	-
plasmid_1	-	-	-	-	-	-	-	-	-	-	-
plasmid_2	-	-	-	-	-	-	-	-	-	-	-
pMG2200	+	-	-	-	-	-	-	-	-	-	-
PML21	-	-	-	-	-	-	-	-	-	-	-
pRE25	-	-	-	-	-	-	-	-	-	-	-
pTEF1	+	-	-	-	-	-	-	-	-	-	-

**pTEF2**	**+**	**-**	**-**	**-**	**-**	**-**	**-**	**-**	**-**	**-**	**-**
**pTEF3**	**-**	**-**	**-**	**-**	**-**	**-**	**-**	**-**	**-**	**-**	**-**
**pTW9**	**+**	**-**	**-**	**-**	**-**	**-**	**-**	**-**	**-**	**-**	**-**
**pWZ1668**	**-**	**-**	**-**	**-**	**-**	**-**	**-**	**-**	**-**	**-**	**-**
**pWZ7140**	**-**	**-**	**-**	**-**	**-**	**-**	**-**	**-**	**-**	**-**	**-**
**pWZ909**	**-**	**-**	**-**	**-**	**-**	**-**	**-**	**-**	**-**	**-**	**-**

1. Chen, Lihong, et al. "VFDB: a reference database for bacterial virulence factors." *Nucleic acids research* 33.suppl 1 (2005): D325-D328.

Table S3: Details of Body-site specific genes of *E.faecalis* strains

Details of Blood specific genes of *E. faecalis* strains.

E. faecalis Strain	Gene ID	Protein_length	Locus_tag	Protein product
E. faecalis DAPTO 512	310625214	400	HMPREF9492_03081	Efflux ABC transporter, permease protein
E. faecalis DAPTO 512	310625215	228	HMPREF9492_03082	ABC transporter, ATP-binding protein
E. faecalis DAPTO 512	310625216	402	HMPREF9492_03083	Conserved hypothetical protein
E. faecalis DAPTO 512	310625217	50	HMPREF9492_03084	Conserved domain protein
E. faecalis DAPTO 512	310625219	209	HMPREF9492_03086	ABC transporter, ATP-binding protein
E. faecalis DAPTO 512	310625220	174	HMPREF9492_03087	Conserved hypothetical protein
E. faecalis DAPTO 512	310625221	396	HMPREF9492_03088	Hypothetical protein
E. faecalis DAPTO 512	310625222	96	HMPREF9492_03089	Circular bacteriocin, circularin A/uberolysin family protein
E. faecalis DAPTO 512	310625587	238	HMPREF9492_02757	Conserved hypothetical protein
E. faecalis DAPTO 512	310625588	101	HMPREF9492_02758	Conserved hypothetical protein
E. faecalis DAPTO 512	310625589	60	HMPREF9492_02759	Conserved hypothetical protein
E. faecalis DAPTO 512	310625590	90	HMPREF9492_02760	Toxin-antitoxin system, antitoxin component, PHD family
E. faecalis DAPTO 512	310625591	86	HMPREF9492_02761	Addiction module toxin, Txe/YoeB family
E. faecalis DAPTO 512	310625807	419	HMPREF9492_02521	Putative cytosine deaminase
E. faecalis DAPTO 512	310625808	406	HMPREF9492_02522	Amidohydrolase
E. faecalis DAPTO 512	310625809	101	HMPREF9492_02523	Hypothetical protein
E. faecalis DAPTO 512	310625822	276	HMPREF9492_02499	Hypothetical protein
E. faecalis DAPTO 512	310625823	261	HMPREF9492_02500	Conserved domain protein
E. faecalis DAPTO 512	310625824	162	HMPREF9492_02501	Conserved domain protein

E. faecalis DAPTO 512	310625825	440	HMPREF9492_02502	Transposase, IS4 family
E. faecalis DAPTO 512	310625872	55	HMPREF9492_02441	Hypothetical protein
E. faecalis DAPTO 512	310625889	56	HMPREF9492_02458	Conserved domain protein
E. faecalis DAPTO 512	310625910	425	HMPREF9492_02413	Hypothetical protein
E. faecalis DAPTO 512	310625911	692	HMPREF9492_02414	ATP-dependent DNA helicase, RecQ family
E. faecalis DAPTO 512	310625912	248	HMPREF9492_02415	Hydrolase, TatD family
E. faecalis DAPTO 512	310625913	425	HMPREF9492_02416	Conserved hypothetical protein
E. faecalis DAPTO 512	310625914	241	HMPREF9492_02417	Hypothetical protein
E. faecalis DAPTO 512	310625915	611	HMPREF9492_02418	Phage putative tail component protein
E. faecalis DAPTO 512	310625916	574	HMPREF9492_02419	UvrD/REP helicase
E. faecalis DAPTO 512	310625917	701	HMPREF9492_02420	Conserved hypothetical protein
E. faecalis DAPTO 512	310625918	321	HMPREF9492_02421	Helix-turn-helix protein
E. faecalis DAPTO 512	310625919	233	HMPREF9492_02422	Conserved domain protein
E. faecalis DAPTO 512	310625924	928	HMPREF9492_02427	Hypothetical protein
E. faecalis DAPTO 512	310625951	513	HMPREF9492_02378	Hypothetical protein
E. faecalis DAPTO 512	310625952	1007	HMPREF9492_02379	Hypothetical protein
E. faecalis DAPTO 512	310625953	156	HMPREF9492_02380	Hypothetical protein
E. faecalis DAPTO 512	310625954	225	HMPREF9492_02381	Hypothetical protein
E. faecalis DAPTO 512	310625955	96	HMPREF9492_02382	LPXTG-motif protein cell wall anchor domain protein
E. faecalis DAPTO 512	310625956	446	HMPREF9492_02383	Trypsin
E. faecalis DAPTO 512	310625957	148	HMPREF9492_02384	Hypothetical protein
E. faecalis DAPTO 512	310625958	151	HMPREF9492_02385	Hypothetical protein
E. faecalis DAPTO 512	310625959	51	HMPREF9492_02386	Hypothetical protein
E. faecalis DAPTO 512	310625960	41	HMPREF9492_02387	Hypothetical protein
E. faecalis DAPTO 512	310626143	202	HMPREF9492_02165	Conserved domain protein
E. faecalis DAPTO 512	310626158	77	HMPREF9492_02146	Helix-turn-helix protein

E. faecalis DAPTO 512	310626159	96	HMPREF9492_02147	Toxin-antitoxin system, toxin component, RelE family
E. faecalis DAPTO 512	310626160	110	HMPREF9492_02148	Toxin-antitoxin system, antitoxin component, AbrB domain protein
E. faecalis DAPTO 512	310626162	148	HMPREF9492_02150	VanZ like family protein
E. faecalis DAPTO 512	310626163	304	HMPREF9492_02151	Serine-type D-Ala-D-Ala carboxypeptidase
E. faecalis DAPTO 512	310626168	385	HMPREF9492_02156	ATPase, histidine kinase-, DNA gyrase B-, and HSP90-like domain protein
E. faecalis DAPTO 512	310626170	165	HMPREF9492_02158	Resolvase
E. faecalis DAPTO 512	310626171	530	HMPREF9492_02159	Transposase
E. faecalis DAPTO 512	310626186	124	HMPREF9492_01475	Semialdehyde dehydrogenase, dimerization domain protein
E. faecalis DAPTO 512	310626450	81	HMPREF9492_01743	Hypothetical protein
E. faecalis DAPTO 512	310626452	168	HMPREF9492_01745	Hypothetical protein
E. faecalis DAPTO 512	310626453	264	HMPREF9492_01746	Hypothetical protein
E. faecalis DAPTO 512	310626604	244	HMPREF9492_01898	PTS system, glucose subfamily, IIA component
E. faecalis DAPTO 512	310626900	98	HMPREF9492_01441	Hypothetical protein
E. faecalis DAPTO 512	310627002	147	HMPREF9492_01338	Conserved domain protein
E. faecalis DAPTO 512	310627231	43	HMPREF9492_00905	Hypothetical protein
E. faecalis DAPTO 512	310627316	157	HMPREF9492_00990	ABC transporter, ATP-binding protein
E. faecalis DAPTO 512	310627318	44	HMPREF9492_00992	Conserved domain protein
E. faecalis DAPTO 512	310627430	42	HMPREF9492_00884	Conserved domain protein
E. faecalis DAPTO 512	310627581	116	HMPREF9492_00755	M42 glutamyl aminopeptidase
E. faecalis DAPTO 512	310627673	313	HMPREF9492_00617	LPXTG-motif protein cell wall anchor domain protein
E. faecalis DAPTO 512	310627868	78	HMPREF9492_00541	Conserved hypothetical protein
E. faecalis DAPTO 512	310627869	164	HMPREF9492_00542	Hypothetical protein
E. faecalis DAPTO 512	310627883	305	HMPREF9492_00556	Hypothetical protein
E. faecalis DAPTO 512	310627884	208	HMPREF9492_00557	Conserved domain protein

E. faecalis DAPTO 512	310627885	188	HMPREF9492_00559	Hypothetical protein
E. faecalis DAPTO 512	310628043	62	HMPREF9492_00368	Conserved domain protein
E. faecalis DAPTO 512	310628082	89	HMPREF9492_00236	Hypothetical protein
E. faecalis R712	291078155	41	HMPREF9377_02380	Hypothetical protein
E. faecalis R712	291078160	60	HMPREF9377_02350	Holliday junction DNA helicase RuvA
E. faecalis R712	291079357	42	HMPREF9377_01054	ABC transporter, permease protein
E. faecalis R712	291079780	58	HMPREF9377_00992	Conserved hypothetical protein
E. faecalis TX0017	315031325	60	HMPREF9500_02848	Hypothetical protein
E. faecalis TX0017	315031684	82	HMPREF9500_02401	Hypothetical protein
E. faecalis TX0017	315031763	137	HMPREF9500_02379	Conserved domain protein
E. faecalis TX0017	315032239	128	HMPREF9500_01883	Site-specific recombinase, phage integrase family
E. faecalis TX0017	315032305	445	HMPREF9500_01855	Trypsin
E. faecalis TX0017	315032306	246	HMPREF9500_01856	Helix-turn-helix protein
E. faecalis TX0017	315032581	99	HMPREF9500_01524	Conserved domain protein
E. faecalis TX0017	315032582	251	HMPREF9500_01525	Hypothetical protein
E. faecalis TX0017	315032633	50	HMPREF9500_01485	Hypothetical protein
E. faecalis TX0017	315033081	169	HMPREF9500_01031	Conserved hypothetical protein
E. faecalis TX0017	315033082	108	HMPREF9500_01032	Hypothetical protein
E. faecalis TX0017	315033102	156	HMPREF9500_01052	Toxin-antitoxin system, toxin component domain protein
E. faecalis TX0017	315033384	83	HMPREF9500_00698	Hypothetical protein
E. faecalis TX0017	315033398	321	HMPREF9500_00712	Conserved hypothetical protein
E. faecalis TX0017	315033841	64	HMPREF9500_00196	Conserved domain protein
E. faecalis TX0027	315034706	84	HMPREF9501_02595	Helix-turn-helix protein
E. faecalis TX0027	315034707	126	HMPREF9501_02596	Toxin-antitoxin system, antitoxin component, Xre family
E. faecalis TX0027	315034708	153	HMPREF9501_02597	Toxin-antitoxin system, toxin component domain

				protein
E. faecalis TX0027	315035257	145	HMPREF9501_01991	Conserved domain protein
E. faecalis TX0027	315035360	58	HMPREF9501_01893	Conserved domain protein
E. faecalis TX0027	315035361	72	HMPREF9501_01894	Conserved hypothetical protein
E. faecalis TX0027	315035859	390	HMPREF9501_01385	Site-specific recombinase, phage integrase family
E. faecalis TX0027	315035864	94	HMPREF9501_01390	Conserved domain protein
E. faecalis TX0027	315035879	182	HMPREF9501_01405	Hypothetical protein
E. faecalis TX0027	315036243	40	HMPREF9501_01082	Hypothetical protein
E. faecalis TX0027	315037188	39	HMPREF9501_00043	Tetracycline resistance determinant leader peptide
E. faecalis TX0031	315152097	377	HMPREF9502_02515	ATPase, AAA family
E. faecalis TX0031	315152098	769	HMPREF9502_02516	Hypothetical protein
E. faecalis TX0031	315152099	91	HMPREF9502_02517	Hypothetical protein
E. faecalis TX0031	315152100	193	HMPREF9502_02518	Hypothetical protein
E. faecalis TX0031	315152106	109	HMPREF9502_02524	Hypothetical protein
E. faecalis TX0031	315152115	998	HMPREF9502_02533	Hypothetical protein
E. faecalis TX0031	315152116	485	HMPREF9502_02534	Hypothetical protein
E. faecalis TX0031	315152117	244	HMPREF9502_02535	IstB-like ATP binding protein
E. faecalis TX0031	315152118	140	HMPREF9502_02536	Hypothetical protein
E. faecalis TX0031	315152119	61	HMPREF9502_02537	Conserved domain protein
E. faecalis TX0031	315152120	517	HMPREF9502_02538	Hypothetical protein
E. faecalis TX0031	315152121	194	HMPREF9502_02539	Transcriptional regulator, TetR family
E. faecalis TX0031	315152122	96	HMPREF9502_02540	Conserved hypothetical protein
E. faecalis TX0031	315152123	230	HMPREF9502_02541	Metallo-beta-lactamase domain protein
E. faecalis TX0031	315152124	238	HMPREF9502_02542	Hypothetical protein
E. faecalis TX0031	315152125	326	HMPREF9502_02543	Conserved hypothetical protein
E. faecalis TX0031	315152126	1274	HMPREF9502_02544	Hypothetical protein

E. faecalis TX0031	315152127	243	HMPREF9502_02545	Hypothetical protein
E. faecalis TX0031	315152128	92	HMPREF9502_02546	Hypothetical protein
E. faecalis TX0031	315152129	539	HMPREF9502_02547	M protein trans-acting positive regulator
E. faecalis TX0031	315152130	207	HMPREF9502_02548	Helix-turn-helix protein
E. faecalis TX0031	315152131	154	HMPREF9502_02549	Conserved hypothetical protein
E. faecalis TX0031	315152132	136	HMPREF9502_02550	Conserved hypothetical protein
E. faecalis TX0031	315152134	298	HMPREF9502_02552	Conserved hypothetical protein
E. faecalis TX0031	315152135	144	HMPREF9502_02553	Transcriptional regulator, MarR family
E. faecalis TX0031	315152136	748	HMPREF9502_02554	Putative excinuclease ABC subunit A
E. faecalis TX0031	315152182	48	HMPREF9502_02051	Hypothetical protein
E. faecalis TX0031	315152198	74	HMPREF9502_02067	Hypothetical protein
E. faecalis TX0031	315152817	163	HMPREF9502_01781	Conserved domain protein
E. faecalis TX0031	315152831	64	HMPREF9502_01795	Conserved domain protein
E. faecalis TX0031	315152893	107	HMPREF9502_01676	Conserved domain protein
E. faecalis TX0031	315153423	265	HMPREF9502_01080	Ser/Thr protein phosphatase family protein
E. faecalis TX0031	315153424	119	HMPREF9502_01081	Hypothetical protein
E. faecalis TX0031	315153425	161	HMPREF9502_01082	Hypothetical protein
E. faecalis TX0031	315153429	438	HMPREF9502_01086	FtsK/SpoIIIE family protein
E. faecalis TX0031	315153430	130	HMPREF9502_01087	Conserved hypothetical protein
E. faecalis TX0031	315153431	165	HMPREF9502_01088	Conserved hypothetical protein
E. faecalis TX0031	315153432	301	HMPREF9502_01089	Conserved domain protein
E. faecalis TX0031	315153433	487	HMPREF9502_01090	RecF/RecN/SMC protein
E. faecalis TX0031	315153475	94	HMPREF9502_01132	Conserved domain protein
E. faecalis TX0031	315153813	99	HMPREF9502_00766	Conserved domain protein
E. faecalis TX0031	315153814	204	HMPREF9502_00767	Conserved domain protein
E. faecalis TX0031	315153930	40	HMPREF9502_00662	Hypothetical protein

E. faecalis TX0031	315154310	88	HMPREF9502_00295	Hypothetical protein
E. faecalis TX0031	315154356	231	HMPREF9502_00127	Conserved hypothetical protein
E. faecalis TX0031	315154361	55	HMPREF9502_00132	Hypothetical protein
E. faecalis TX0031	315154434	181	HMPREF9502_00206	Conserved hypothetical protein
E. faecalis TX0031	315154439	216	HMPREF9502_00211	Bacterial SH3 domain protein
E. faecalis TX0043	315154825	80	HMPREF9503_02643	Hypothetical protein
E. faecalis TX0043	315156032	280	HMPREF9503_01272	Helix-turn-helix protein
E. faecalis TX0043	315156033	192	HMPREF9503_01273	Conserved domain protein
E. faecalis TX0043	315156034	350	HMPREF9503_01274	Conserved hypothetical protein
E. faecalis TX0043	315156039	313	HMPREF9503_01279	CorA-like Mg2+ transporter protein
E. faecalis TX0043	315156040	277	HMPREF9503_01280	Conserved hypothetical protein
E. faecalis TX0043	315156492	376	HMPREF9503_00959	Phage lysozyme
E. faecalis TX0043	315157056	88	HMPREF9503_00137	Helix-turn-helix protein
E. faecalis TX0043	315157059	99	HMPREF9503_00140	Conserved domain protein
E. faecalis TX0043	315157063	101	HMPREF9503_00144	Conserved domain protein
E. faecalis TX0043	315157073	47	HMPREF9503_00154	Conserved hypothetical protein
E. faecalis TX0043	315157077	108	HMPREF9503_00158	Hypothetical protein
E. faecalis TX0043	315157150	489	HMPREF9503_00231	Hypothetical protein
E. faecalis TX0043	315157291	42	HMPREF9503_00376	Hypothetical protein
E. faecalis TX0102	310630537	44	HMPREF9504_00681	Hypothetical protein
E. faecalis TX0102	310629244	61	HMPREF9504_01844	Conserved domain protein
E. faecalis TX0109	306500093	81	HMPREF9505_02327	Hypothetical protein
E. faecalis TX0109	306500365	51	HMPREF9505_01990	Hypothetical protein
E. faecalis TX0109	306501411	165	HMPREF9505_01122	Hypothetical protein
E. faecalis TX0630	315576928	65	HMPREF9511_02889	Hypothetical protein
E. faecalis TX0630	315577562	104	HMPREF9511_02279	Helix-turn-helix protein

E. faecalis TX0630	315577563	103	HMPREF9511_02280	Toxin-antitoxin system, antitoxin component, Xre family
E. faecalis TX0630	315577565	199	HMPREF9511_02282	Conserved hypothetical protein
E. faecalis TX0630	315577568	46	HMPREF9511_02285	Hypothetical protein
E. faecalis TX0630	315577981	47	HMPREF9511_01844	Hypothetical protein
E. faecalis TX0630	315578238	127	HMPREF9511_01585	Transcriptional regulator, BlaI/MecI/CopY family
E. faecalis TX0630	315578943	77	HMPREF9511_00854	Conserved domain protein
E. faecalis TX0630	315578944	74	HMPREF9511_00855	Hypothetical protein
E. faecalis TX0630	315579421	147	HMPREF9511_00308	Conserved domain protein
E. faecalis TX0630	315579442	231	HMPREF9511_00329	DNA (cytosine-5-)-methyltransferase
E. faecalis TX0645	315160917	57	HMPREF9513_02532	Hypothetical protein
E. faecalis TX0645	315160990	100	HMPREF9513_02491	Hypothetical protein
E. faecalis TX0645	315161055	75	HMPREF9513_02408	Hypothetical protein
E. faecalis TX0645	315161797	121	HMPREF9513_01697	Conserved domain protein
E. faecalis TX0645	315162200	547	HMPREF9513_01304	Conserved hypothetical protein
E. faecalis TX0645	315162230	76	HMPREF9513_01335	Helix-turn-helix protein
E. faecalis TX0645	315162231	164	HMPREF9513_01336	Helix-turn-helix protein
E. faecalis TX0645	315162671	298	HMPREF9513_00793	Conserved hypothetical protein
E. faecalis TX0645	315162837	50	HMPREF9513_00633	Hypothetical protein
E. faecalis TX0645	315162973	82	HMPREF9513_00443	Hypothetical protein
E. faecalis TX0645	315162975	71	HMPREF9513_00445	Conserved domain protein
E. faecalis TX0860	306503557	56	HMPREF9515_02086	Toxin-antitoxin system, antitoxin component, ribbon-helix-helix fold domain protein
E. faecalis TX0860	306503998	81	HMPREF9515_01674	Hypothetical protein
E. faecalis TX0860	306503999	88	HMPREF9515_01675	Hypothetical protein

Details of GI Tract specific genes of *E. faecalis* strains

E. faecalis Strain	Gene ID	Protein_length	Locus_tag	Protein product
E. faecalis V583	29374965	79	EF0326	Hypothetical protein
E. faecalis V583	29374966	100	EF0327	Hypothetical protein
E. faecalis V583	29374985	162	EF0346	Hypothetical protein
E. faecalis V583	29375215	37	EF0610	Hypothetical protein
E. faecalis V583	29375225	54	EF0622	Hypothetical protein
E. faecalis V583	29375228	57	EF0626	Hypothetical protein
E. faecalis V583	29375397	31	EF0808	Hypothetical protein
E. faecalis V583	29376155	34	EF1594	Hypothetical protein
E. faecalis V583	29376628	105	EF2119	Hypothetical protein
E. faecalis V583	29376631	39	EF2122	Hypothetical protein
E. faecalis V583	29376635	116	EF2126	Hypothetical protein
E. faecalis V583	29376799	275	EF2296	Vancomycin B-type resistance protein VanW
E. faecalis V583	29376801	447	EF2298	Sensor histidine kinase VanSB
E. faecalis V583	29376802	220	EF2299	DNA-binding response regulator VanRB
E. faecalis V583	29376814	705	EF2312	DNA topoisomerase III
E. faecalis V583	29376822	825	EF2320	TraE protein
E. faecalis V583	29376825	289	EF2324	Hypothetical protein
E. faecalis V583	29376828	43	EF2327	Hypothetical protein
E. faecalis V583	29376829	591	EF2328	TraG family protein
E. faecalis V583	29376861	34	EF2363	Hypothetical protein
E. faecalis V583	29377063	37	EF2576	Hypothetical protein
E. faecalis PC1.1	294451417	47	CUI_1333	Hypothetical protein

E. faecalis PC1.1	294451462	79	CUI_1529	Conserved domain protein
E. faecalis PC1.1	294453083	263	CUI_2523	YhgE/Pip N-terminal domain protein
E. faecalis TX0104	227072478	86	HMPREF0348_3048	Conserved hypothetical protein
E. faecalis TX0104	227072737	221	HMPREF0348_2782	Conserved hypothetical protein
E. faecalis TX0104	227072860	512	HMPREF0348_2619	SPP1 family phage portal protein
E. faecalis TX0104	227072861	313	HMPREF0348_2620	Phage minor head protein
E. faecalis TX0104	227072862	205	HMPREF0348_2621	Phage scaffold protein
E. faecalis TX0104	227072863	312	HMPREF0348_2622	Phage protein
E. faecalis TX0104	227072864	124	HMPREF0348_2623	Conserved hypothetical protein
E. faecalis TX0104	227072865	94	HMPREF0348_2624	Conserved hypothetical protein
E. faecalis TX0104	227072866	115	HMPREF0348_2625	Phage protein
E. faecalis TX0104	227072867	113	HMPREF0348_2626	Conserved hypothetical protein
E. faecalis TX0104	227072868	214	HMPREF0348_2627	Conserved hypothetical protein
E. faecalis TX0104	227072870	158	HMPREF0348_2629	Conserved hypothetical protein
E. faecalis TX0104	227072871	90	HMPREF0348_2630	Hypothetical protein
E. faecalis TX0104	227072872	1298	HMPREF0348_2631	Tail protein
E. faecalis TX0104	227072873	280	HMPREF0348_2632	Conserved hypothetical protein
E. faecalis TX0104	227072874	975	HMPREF0348_2633	Possible reticulocyte binding protein
E. faecalis TX0104	227072875	118	HMPREF0348_2634	Hypothetical protein
E. faecalis TX0104	227072876	90	HMPREF0348_2635	Hypothetical protein
E. faecalis TX0104	227072878	80	HMPREF0348_2637	Phi11 family holin
E. faecalis TX0104	227072879	248	HMPREF0348_2638	Possible N-acetylmuramoyl-L-alanine amidase
E. faecalis TX0104	227072882	353	HMPREF0348_2641	Conserved hypothetical protein
E. faecalis TX0104	227072918	72	HMPREF0348_2595	Conserved hypothetical protein
E. faecalis TX0104	227072924	92	HMPREF0348_2601	Hypothetical protein
E. faecalis TX0104	227072954	377	HMPREF0348_2587	Integrase family protein

E. faecalis TX0104	227072955	139	HMPREF0348_2588	Hypothetical protein
E. faecalis TX0104	227072956	262	HMPREF0348_2589	Hypothetical protein
E. faecalis TX0104	227072959	73	HMPREF0348_2592	Hypothetical protein
E. faecalis TX0104	227072960	106	HMPREF0348_2593	Hypothetical protein
E. faecalis TX0104	227073230	228	HMPREF0348_2284	Possible dGTPase
E. faecalis TX0104	227073478	146	HMPREF0348_2041	Possible deaminase
E. faecalis TX0104	227073693	99	HMPREF0348_1848	ABC superfamily ATP binding cassette transporter, ABC protein
E. faecalis TX0104	227073715	81	HMPREF0348_1827	Conserved hypothetical protein
E. faecalis TX0104	227073833	68	HMPREF0348_1696	Conserved hypothetical protein
E. faecalis TX0104	227074037	39	HMPREF0348_1538	Conserved hypothetical protein
E. faecalis TX0104	227074200	68	HMPREF0348_1310	Conserved hypothetical protein
E. faecalis TX0104	227074642	323	HMPREF0348_0871	Radical SAM domain protein protein
E. faecalis TX0104	227074643	50	HMPREF0348_0872	Hypothetical protein
E. faecalis TX0104	227074950	41	HMPREF0348_0534	Hypothetical protein
E. faecalis TX0104	227074960	54	HMPREF0348_0544	Hypothetical protein
E. faecalis TX0104	227075050	105	HMPREF0348_0428	Hypothetical protein
E. faecalis TX0104	227075222	202	HMPREF0348_0335	ABC superfamily ATP binding cassette transporter, ABC/membrane protein
E. faecalis TX1302	315163601	303	HMPREF9516_02780	Transposase, IS4 family
E. faecalis TX1302	315164446	45	HMPREF9516_01968	Hypothetical protein
E. faecalis TX1302	315165051	108	HMPREF9516_01312	Conserved domain protein
E. faecalis TX1302	315165826	46	HMPREF9516_00525	Hypothetical protein
E. faecalis TX1322	229307210	102	HMPREF0349_2937	Conserved hypothetical protein
E. faecalis TX1322	229307631	456	HMPREF0349_2515	SEC-C motif domain protein
E. faecalis TX1322	229307647	301	HMPREF0349_2417	Conserved hypothetical protein
E. faecalis TX1322	229307648	328	HMPREF0349_2418	Hypothetical protein

E. faecalis TX1322	229307649	245	HMPREF0349_2419	Bifunctional S24 family peptidase/transcriptional regulator
E. faecalis TX1322	229307650	65	HMPREF0349_2420	Conserved hypothetical protein
E. faecalis TX1322	229307651	65	HMPREF0349_2421	Hypothetical protein
E. faecalis TX1322	229307653	120	HMPREF0349_2423	Conserved hypothetical protein
E. faecalis TX1322	229307656	300	HMPREF0349_2426	Conserved hypothetical protein
E. faecalis TX1322	229307657	294	HMPREF0349_2427	DNA replication protein
E. faecalis TX1322	229307658	64	HMPREF0349_2428	Hypothetical protein
E. faecalis TX1322	229307659	67	HMPREF0349_2429	Hypothetical protein
E. faecalis TX1322	229307660	90	HMPREF0349_2430	Hypothetical protein
E. faecalis TX1322	229307661	75	HMPREF0349_2431	Hypothetical protein
E. faecalis TX1322	229307662	54	HMPREF0349_2432	Hypothetical protein
E. faecalis TX1322	229307663	188	HMPREF0349_2433	Hypothetical protein
E. faecalis TX1322	229307664	337	HMPREF0349_2434	Hypothetical protein
E. faecalis TX1322	229307665	391	HMPREF0349_2435	Encapsidation protein
E. faecalis TX1322	229307666	192	HMPREF0349_2436	Hypothetical protein
E. faecalis TX1322	229307667	79	HMPREF0349_2437	Hypothetical protein
E. faecalis TX1322	229307670	142	HMPREF0349_2440	Holin
E. faecalis TX1322	229307672	233	HMPREF0349_2442	Possible lower collar protein
E. faecalis TX1322	229307673	57	HMPREF0349_2443	Hypothetical protein
E. faecalis TX1322	229307674	82	HMPREF0349_2444	Hypothetical protein
E. faecalis TX1322	229307675	100	HMPREF0349_2445	Hypothetical protein
E. faecalis TX1322	229307676	100	HMPREF0349_2446	Hypothetical protein
E. faecalis TX1322	229307677	108	HMPREF0349_2447	Hypothetical protein
E. faecalis TX1322	229307678	127	HMPREF0349_2448	Hypothetical protein
E. faecalis TX1322	229307679	470	HMPREF0349_2449	Conserved hypothetical protein
E. faecalis TX1322	229307680	334	HMPREF0349_2450	Upper collar protein

E. faecalis TX1322	229307681	825	HMPREF0349_2451	Hypothetical protein
E. faecalis TX1322	229307682	245	HMPREF0349_2452	Hypothetical protein
E. faecalis TX1322	229307683	618	HMPREF0349_2453	Tail protein
E. faecalis TX1322	229307684	460	HMPREF0349_2454	Hypothetical protein
E. faecalis TX1322	229307685	63	HMPREF0349_2455	Conserved hypothetical protein
E. faecalis TX1322	229307686	154	HMPREF0349_2456	Hypothetical phage protein
E. faecalis TX1322	229307845	62	HMPREF0349_2136	Hypothetical protein
E. faecalis TX1322	229307942	111	HMPREF0349_2233	Hypothetical protein
E. faecalis TX1322	229308049	56	HMPREF0349_2103	Conserved hypothetical protein
E. faecalis TX1322	229308158	107	HMPREF0349_2028	Conserved hypothetical protein
E. faecalis TX1322	229308851	181	HMPREF0349_1225	Hypothetical protein
E. faecalis TX1322	229308852	177	HMPREF0349_1226	Conserved hypothetical protein
E. faecalis TX1322	229308853	48	HMPREF0349_1227	Hypothetical protein
E. faecalis TX1322	229308855	226	HMPREF0349_1229	Possible transcriptional regulator PlcR
E. faecalis TX1322	229308874	156	HMPREF0349_1220	Possible ABC-2 type transporter
E. faecalis TX1322	229308875	219	HMPREF0349_1221	ABC superfamily ATP binding cassette transporter, ABC protein
E. faecalis TX1322	229308877	362	HMPREF0349_1223	Radical SAM domain protein
E. faecalis TX1322	229308913	47	HMPREF0349_1167	Conserved hypothetical protein
E. faecalis TX1322	229309504	235	HMPREF0349_0614	Hypothetical protein
E. faecalis TX1322	229309716	56	HMPREF0349_0388	Hypothetical protein
E. faecalis TX1322	229309754	176	HMPREF0349_0426	Conserved hypothetical protein
E. faecalis TX1341	315166415	66	HMPREF9517_02992	Conserved domain protein
E. faecalis TX1341	315166416	243	HMPREF9517_02993	Hypothetical protein
E. faecalis TX1341	315166417	262	HMPREF9517_02994	Conserved hypothetical protein
E. faecalis TX1341	315166418	236	HMPREF9517_02995	ABC transporter, ATP-binding protein
E. faecalis TX1341	315166419	764	HMPREF9517_02996	ABC transporter, ATP-binding protein

E. faecalis TX1341	315166420	83	HMPREF9517_02997	Hypothetical protein
E. faecalis TX1341	315166421	522	HMPREF9517_02998	Asparagine synthase
E. faecalis TX1341	315166422	52	HMPREF9517_02999	Hypothetical protein
E. faecalis TX1341	315166423	654	HMPREF9517_03000	ATPase, histidine kinase-, DNA gyrase B-, and HSP90-like domain protein
E. faecalis TX1341	315166424	198	HMPREF9517_03001	Transcriptional regulator, LuxR family
E. faecalis TX1341	315166885	211	HMPREF9517_02566	MATE domain protein
E. faecalis TX1341	315167317	546	HMPREF9517_01966	Hypothetical protein
E. faecalis TX1341	315168252	44	HMPREF9517_01115	Hypothetical protein
E. faecalis TX1341	315168535	387	HMPREF9517_00882	LPXTG-motif protein cell wall anchor domain protein
E. faecalis TX1341	315168536	199	HMPREF9517_00883	Acetyltransferase, GNAT family
E. faecalis TX1341	315168844	64	HMPREF9517_00294	Hypothetical protein
E. faecalis TX1341	315169229	254	HMPREF9517_00176	Conserved hypothetical protein
E. faecalis TX1341	315169230	234	HMPREF9517_00177	Hypothetical protein
E. faecalis TX1341	315169297	75	HMPREF9517_00065	Conserved domain protein
E. faecalis TX1342	315170884	48	HMPREF9518_01349	Hypothetical protein
E. faecalis TX1342	315170944	180	HMPREF9518_01196	Hypothetical protein
E. faecalis TX1342	315171080	552	HMPREF9518_00966	Hypothetical protein
E. faecalis TX1342	315171081	243	HMPREF9518_00967	ABC transporter, ATP-binding protein
E. faecalis TX1342	315172139	56	HMPREF9518_00045	Conserved hypothetical protein
E. faecalis TX1342	315172146	83	HMPREF9518_00052	Conserved domain protein
E. faecalis TX1346	315172466	60	HMPREF9519_02554	Hypothetical protein
E. faecalis TX1346	315173385	143	HMPREF9519_01613	Toxin-antitoxin system, toxin component family protein
E. faecalis TX1467	329569637	486	HMPREF9520_03243	RecF/RecN/SMC protein
E. faecalis TX1467	329577287	47	HMPREF9520_00890	Hypothetical protein
E. faecalis TX1467	329578214	94	HMPREF9520_00009	Hypothetical protein

Details of UG Tract specific genes of *E. faecalis* strains

E. faecalis **Strain**	**Gene ID**	**Protein_length**	**Locus_tag**	**Protein product**
E. faecalis ATCC 29200	229306991	46	HMPREF0345_0038	Hypothetical protein
E. faecalis ATCC 29200	229305362	57	HMPREF0345_1708	Hypothetical protein
E. faecalis HH22	227174834	154	HMPREF0346_3198	Conserved hypothetical protein
E. faecalis HH22	227176840	54	HMPREF0346_1179	Conserved hypothetical protein

Details of Oral specific genes of *E. faecalis* strains

E. faecalis **Strain**	**Gene ID**	**Protein_length**	**Locus_tag**	**Protein product**
E. faecalis 62	384517240	341	EF62_0526	Hypothetical protein

Table S4 – References of Pathogenic and Commensal nature of *E. faecalis* strains

No.	Organism Name	Pathogenicity	Ref. No.
1	*E. faecalis V583*	Pathogenic	1
2	*E. faecalis 62*	Commensal	2
3	*E. faecalis str. Symbioflor 1*	Commensal	3
4	*E. faecalis DAPTO 512*	Pathogenic	4
5	*E. faecalis DAPTO 516*	Pathogenic	4
6	*E. faecalis R712*	Pathogenic	4
7	*E. faecalis S613*	Pathogenic	4
8	*E. faecalis TX0012*	Uncharacterized	-
9	*E. faecalis TX0017*	Uncharacterized	-
10	*E. faecalis TX0027*	Uncharacterized	-
11	*E. faecalis TX0031*	Uncharacterized	-
12	*E. faecalis TX0043*	Uncharacterized	-
13	*E. faecalis TX0102*	Uncharacterized	-
14	*E. faecalis TX0109*	Uncharacterized	-
15	*E. faecalis TX0630*	Pathogenic	5
16	*E. faecalis TX0645*	Pathogenic	6
17	*E. faecalis TX0860*	Uncharacterized	-
18	*E. faecalis TX2141*	Uncharacterized	-
19	*E. faecalis PC1.1*	Commensal	7
20	*E. faecalis TX0104*	Pathogenic	8
21	*E. faecalis TX1302*	Uncharacterized	-
22	*E. faecalis TX1322*	Uncharacterized	-
23	*E. faecalis TX1341*	Uncharacterized	-
24	*E. faecalis TX1342*	Uncharacterized	-
25	*E. faecalis TX1346*	Uncharacterized	-
26	*E. faecalis TX1467*	Uncharacterized	-
27	*E. faecalis TX2134*	Uncharacterized	-
28	*E. faecalis TX2137*	Pathogenic	9
29	*E. faecalis TX4244*	Uncharacterized	-
30	*E. faecalis ATCC 29200*	Uncharacterized	-
31	*E. faecalis HH22*	Pathogenic	10
32	*E. faecalis TX0312*	Uncharacterized	-
33	*E. faecalis TX0635*	Pathogenic	11
34	*E. faecalis TX0855*	Uncharacterized	-
35	*E. faecalis TX4248*	Uncharacterized	-
36	*E. faecalis TUSoD Ef11*	Uncharacterized	-

References:

1. McBride, Shonna M., et al. "Genetic diversity among Enterococcus faecalis." PloS one 2.7 (2007): e582.

2. Brede, Dag Anders, et al. "Complete genome sequence of the commensal *Enterococcus faecalis* 62, isolated from a healthy Norwegian infant." Journal of Bacteriology 193.9 (2011): 2377-2378.

3. Fritzenwanker, Moritz, et al. "Complete genome sequence of the probiotic *Enterococcus faecalis* Symbioflor 1 clone DSM 16431." Genome announcements 1.1 (2013): e00165-12.

4. Munoz-Price, L. Silvia, Karen Lolans, and John P. Quinn. "Emergence of resistance to daptomycin during treatment of vancomycin-resistant *Enterococcus faecalis* infection." Clinical infectious diseases 41.4 (2005): 565-566.

5. Coque, Teresa M., and Barbara E. Murray. "Identification of *Enterococcus faecalis* strains by DNA hybridization and pulsed-field gel electrophoresis."Journal of clinical microbiology 33.12 (1995): 3368.

6. Ruiz-Garbajosa, Patricia, et al. "Multilocus sequence typing scheme for *Enterococcus faecalis* reveals hospital-adapted genetic complexes in a background of high rates of recombination." Journal of Clinical Microbiology44.6 (2006): 2220-2228.

7. Cuív, Páraic Ó., et al. "Draft genome sequence of *Enterococcus faecalis* PC1. 1, a candidate probiotic strain isolated from human feces." Genome announcements 1.1 (2013): e00160-12.

8. Solheim, Margrete, et al. "Comparative genomic analysis reveals significant enrichment of mobile genetic elements and genes encoding surface structure-proteins in hospital-associated clonal complex 2 *Enterococcus faecalis*." BMC microbiology 11.1 (2011): 1.

9. Bourgogne, Agathe, et al. "Large scale variation in *Enterococcus faecalis* illustrated by the genome analysis of strain OG1RF." Genome biology 9.7 (2008): 1-16.

10. Murray, B. E., and B. Mederski-Samaroj. "Transferable beta-lactamase. A new mechanism for in vitro penicillin resistance in *Streptococcus faecalis*."Journal of Clinical Investigation 72.3 (1983): 1168.

11. Nallapareddy, Sreedhar R., et al. "Molecular characterization of a widespread, pathogenic, and antibiotic resistance-receptive *Enterococcus faecalis* lineage and dissemination of its putative pathogenicity island."Journal of bacteriology 187.16 (2005): 5709-5718.
